# Amino Acid–Viologen Hybrids: Synthesis, Cucurbituril Host–Guest Chemistry, and Implementation on the Production of Peptides

**DOI:** 10.1021/acs.joc.1c02040

**Published:** 2021-12-10

**Authors:** Liliana Barravecchia, Iago Neira, Elena Pazos, Carlos Peinador, Marcos D. García

**Affiliations:** † Universidade da Coruña, Centro de Investigacións Científicas Avanzadas (CICA), Elviña, 15071 A Coruña, Spain; ‡ Universidade da Coruña, Departamento de Química, Facultade de Ciencias, Zapateira, 15071 A Coruña, Spain

## Abstract

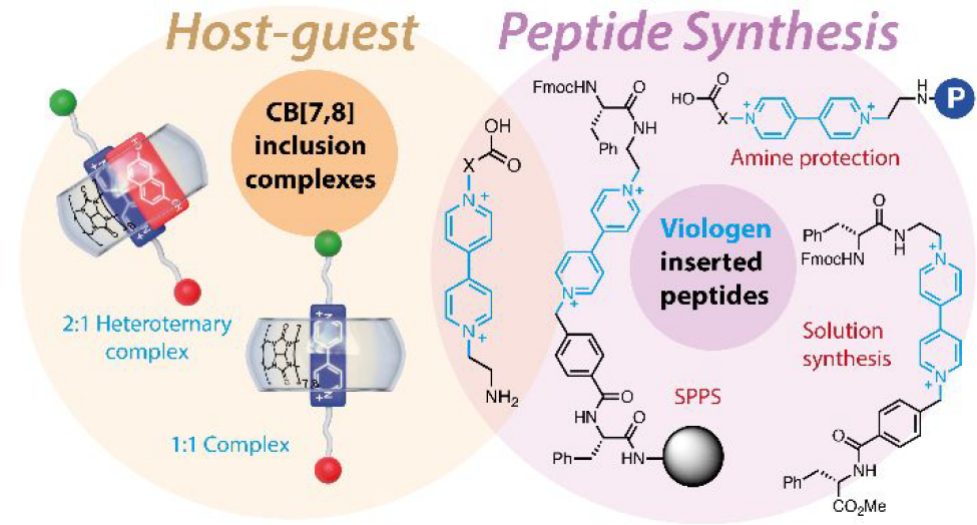

We present herein the development of a series of viologen–amino acid hybrids, obtained in good yields either by successive alkylations of 4,4′-bipyridine, or by Zincke reactions followed by a second alkylation step. The potential of the obtained amino acids has been exemplified, either as typical guests of the curcubituril family of hosts (particularly CB[7]/[8]) or as suitable building blocks for the solution/solid-phase synthesis of two model tripeptides with the viologen core inserted within their sequences.

Viologens (**V**s), compounds resulting from the diquaternization of 4,4′-bipyridine, are one of the most studied classes of stimuli-responsive moieties in chemistry, mainly due to their synthetic accessibility and adjustable properties, such as reversible redox behavior and π-acceptor character.^1^ Consequently, this broad family of organic salts has been extensively used in a variety of practical applications,^2^ including the development of **V**-based electrochromic materials^3^ or, more recently, aqueous redox flow batteries.^4,5^

Furthermore, their high tunability and stimuli-responsiveness have made **V**s prime components on the evolution of supramolecular chemistry. For instance, **V**s can be found as the key parts of the family of macrocyclic receptors known as ExBoxes developed by Stoddart et al.^6^ or as archetypical guests of relevant hosts^7^ such as aryl-containing coronands,^8^ pillarenes,^9^ or cucurbiturils^10,11^ (referred in this paper as CB[*n*]s, *n* = 7,8; Figure 1). In particular, the host–guest chemistry of viologens and CB[8] is currently highly significant,^12^ as heteroternary complexes can be prepared in a predictable fashion, typically by using a **V** as first guest and an electron donor as second (**D**). Furthermore, the very accessible and reversible first reduction potential of **V**s enables the controlled assembly/disassembly of the obtained CB[8]:**V**:**D** [3]pseudorotaxane in very convenient experimental conditions. Hence, this redox-controlled “handcuff strategy” has become the weapon of choice not only for the transient heteroligation of discrete small molecules by CB[8]^10−13^ but also for the creation of more complex assemblies.^10−14^

**Figure 1:**
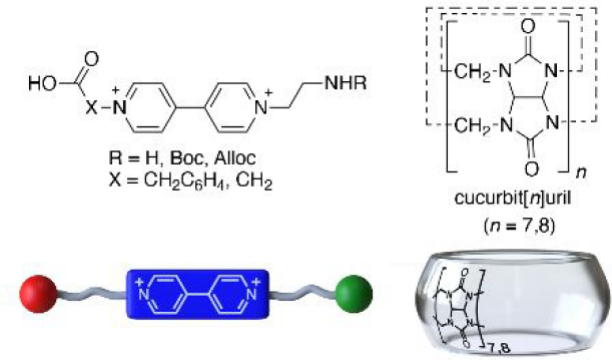
Schematic depiction of the **V**–amino acid hybrids discussed in this work and of the CB[*n*] (*n* = 7, 8) hosts.

Following our interest in the supramolecular chemistry of pyridinium salts,^15^ we initiated a research program focused on the CB[*n*]-based modification of peptides owning appropriate viologen moieties as binding motifs.^16^ In this context, we found the lack of a general methodology for the insertion of the **V** core within peptide sequences, with most of the procedures reported so far being focused on the end-capping or lateral chain modification of the oligomer with the electroactive motif.^16−18^ Consequently, we envisaged the synthesis of a series of viologen–amino acid hybrids (Figure 1) that would potentially allow for their implementation in liquid or solid phase peptide synthesis (L/SPPS) and could retain the characteristics required for the CB[7,8]-based molecular recognition.

Considering the well-established methodology for the synthesis of unsymmetrical **V**s,^2,19^ we first tackled the synthesis of a **V**–amino acid hybrid by direct introduction of the envisioned functional groups through successive alkylation reactions of the 4,4′-bipyridine (**B**_**P**_) core. As shown in Scheme 1, the unprotected amino acid **2**H·3Cl could be prepared by reaction of **B**_**P**_ with commercially available 2-bromoethan-1-amonium bromide, followed by a second alkylation of the intermediate **1**H·2Br with ethyl 2-bromoacetate, and a final step for the hydrolysis of the corresponding ester with aqueous HCl, which produces as well the complete metathesis of the bromide counterions, leading to the trichloride salt in a decent 36% overall yield.^20^

**Scheme 1:**
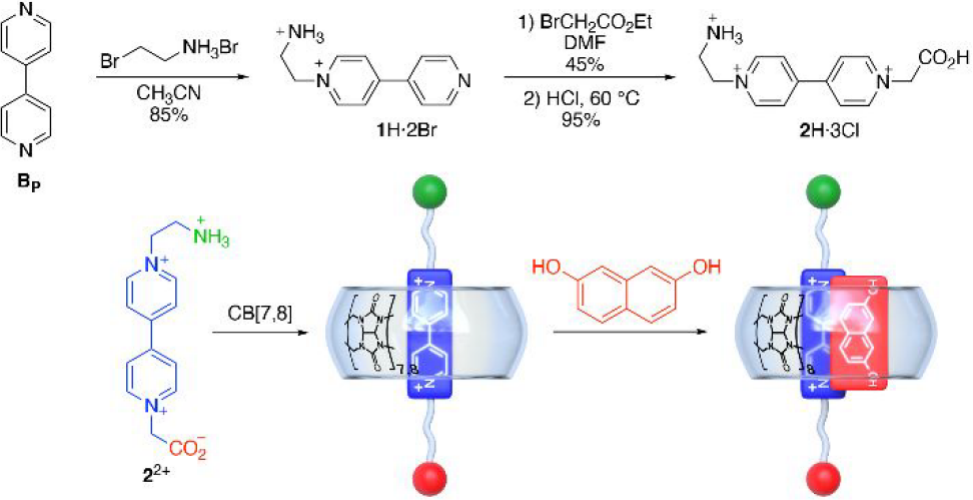
Synthesis of Amino Acid **2**H·3Cl and Representation of the Binary CB[7,8]:**2**^2+^ and Heteroternary CB[8]:**2**^2+^:2,7-DHN Complexes

To test the ability of the obtained amino acid as appropriate guest for CB[*n*]s, we proceeded to study the complexation of the zwitterionic form **2**^2+^ by CB[7]/[8] in buffered aqueous media at pD = 7; a fact that would simplify the assessing of the binding interactions by means of ^1^H NMR spectroscopy.^21^ Both in the case of CB[7] and CB[8], the changes observed in the NMR spectra of guest **2**^2+^ upon addition of increasing quantities of host^22^ are in agreement with the complexation of the **V** moiety following a fast, but near coalescence, exchange rate on the NMR time scale. In both cases, the complexation-induced shifts (CISs) for equimolar mixtures of host and guest (see, for instance, Figure 2a)^22^ are fully consistent with the inclusion process producing 1:1 symmetric pseudorotaxanes. In essence, the shielding of the signals attributable to the viologen core of **2**^2+^ as well as the slight deshielding of the methylene pendant groups of the guest suggest the expected binding mode with the **V** core inserted within the cavity of the hosts as in similar systems.^10^ ESI-MS spectrometry also verified the formation of the binary complexes, with intense peaks corresponding to the expected species being detected for [CB[7]:**2**]^+2^ at (*m*/*z*) calcd 711.7428, found 711.7434 and for [CB[8]:**2**]^+2^ at (*m*/*z*) calcd 794.7674, found 794.7679.^22^ Furthermore, UV–vis titration experiments allowed for the assessment of the association constants, with the obtained data fitting appropriately to 1:1 isotherms with *K*_a_ (CB[7]:**2**^2+^) = (5.7 ± 0.5)10^5^ M^–1^ and *K*_a_ (CB[8]:**2**) = (5.2 ± 0.3) 10^5^ M^–1^ (Figure 2b), values in good agreement with those previously reported for similar systems.^10^

**Figure 2:**
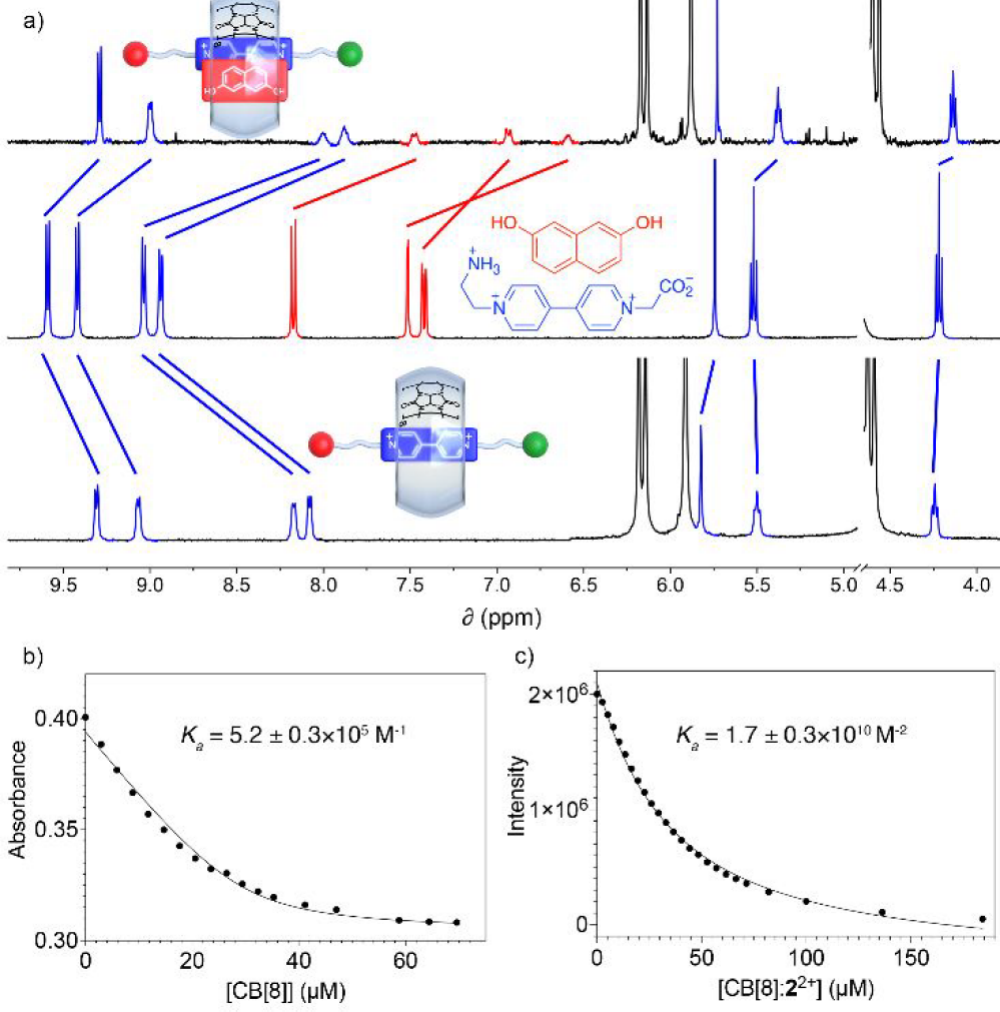
(a) Partial ^1^H NMR spectra (D_2_O, 500 MHz) for equimolar (1 mM) mixtures at 343.15 K of (middle) **2**^2+^ and 2,7-DHN, (top) **2**^2+^, 2,7-DHN and CB[8], and (bottom) **2**^2+^ and CB[8]. (b) Fitting of the observed variation in the absorption at λ = 272 nm of a 29.4 μM **2**^2+^ solution in phosphate buffer (50 mM, pH = 7.0), upon addition of increasing concentrations of CB[8]. (c) Fitting of the observed variation in the fluorescence emission at λ_em_= 345 nm of a 10 μM 2,7-DHN solution in phosphate buffer (50 mM, pH = 7.0), upon addition of increasing concentrations of CB[8]:**2**^**2+**^.

Finally, the obtention of the heteroternary complex between CB[8], **2**^2+^, and 2,7-DHN, as a typical second guest, was also tested by NMR (Figure 2a). Although some of the resonances for the two guests disappear because of a near-coalescence exchange regime on the technique at rt,^22^ the problem was surpassed by recording the experiment at 343.15 K for a 1:1:1 mixture of the compounds at 1 mM (Figure 2a), clearly showing the expected CISs for this type of complexes. Furthermore, the inclusion of 2,7-DHN as a second guest was also corroborated by fluorescence titrations, which allowed us to estimate the overall *K*_a_ (CB[8]:**2**^2+^:2,7-DHN) = (1.7 ± 0.3) 10^10^ M^–2^ (Figure 2c).^22^

Following the development of the **V**-containing amino acids for peptide synthesis, we envisaged two main modifications on the previously discussed synthetic route: (a) replacement of the 1-(carboxymethyl)pyridin-1-ium moiety for a more stable acid functionality (i.e., 1-(4-carboxybenzyl)pyridin-1-ium),^18^ able to surpass potential decomposition by decarboxylation (*vide supra*)^20^ and (b) the introduction of suitable amino protecting groups within our **V**–amino acid hybrids.^23^ Consequently, we decided to tackle first the obtention of compound Boc-**4·**2Cl, a *tert*-butylcarbonyl (Boc)-*N*-protected derivative suitably protected for LPPS. In this case, the **V**–amino acid hybrid was synthesized in a good 49% overall yield, first by the Zincke reaction between readily available *N*-Boc-ethylene diamine and the 2,4-dinitrobenzene-activated salt of **B**_**P**_,^19^ followed by a subsequent alkylation of intermediate **3·**Cl with 4-(chloromethyl)benzoic acid (Scheme 2).

**Scheme 2:**
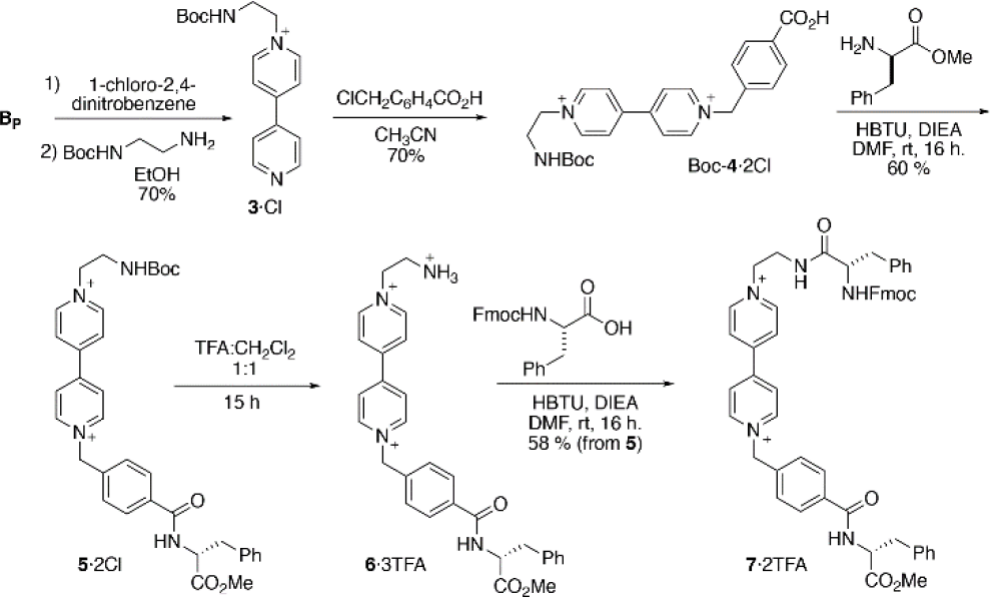
Synthesis of the *N*-Protected **V**–Amino Acid Hybrid Boc-**4·**2Cl and Its Implementation on the Synthesis of the Model Tripeptide **7·**2TFA

With the *N*-protected amino acid Boc-**4·**2Cl in our hands, we proceeded to assess its use on LPPS by addressing the preparation of the simple model tripeptide Fmoc-l-Phe**-4-**d**-**Phe-OMe·2TFA (**7**·2TFA). The synthesis of this compound was performed from the *C*- to *N*-terminus so, first, Boc-**4·**2Cl was coupled to the d-phenylalanine methyl ester to give the dipeptide **5·**2Cl. Next, cleavage of the Boc group with TFA resulted in the ammonium salt **6·**3TFA, which was subsequently used without further purification on the final coupling with Fmoc-l-Phe-OH leading to **7·**2TFA. The compound was obtained on a decent 35% overall yield, with an analytical sample being purified by HPLC, and extensively characterized by ESI-MS spectrometry and NMR spectroscopy (Figure 3).

**Figure 3:**
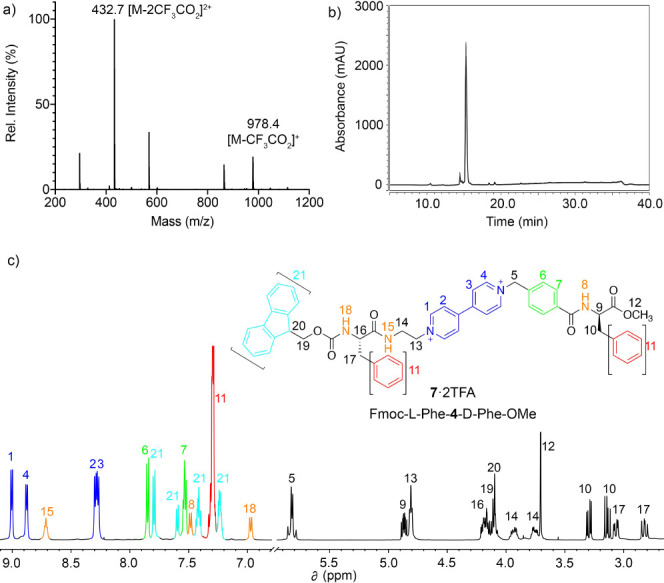
(a) MS spectrum corresponding to the peak at *t*_R_ = 16 min of the HPLC chromatogram at 220 nm (b) for **7·**2TFA. (c) Partial ^1^H NMR spectra (CD_3_CN, 500 MHz) for **7·**2TFA including the assignation based on 1D and 2D NMR.

Among other features, the assignation of the ^1^H NMR spectrum of **7·**2TFA allows us to identify in the aromatic region not only distinctive resonances attributable to the benzyl, phenylene, and Fmoc moieties but also the other four characteristic doublets of the **V** core. Regarding the ESI-MS, the spectrogram shows the signal attributable to the **7**^2+^ cation as the base peak at *m*/*z* calcd for [M – 2CF_3_CO_2_]^2+^ 432.6914, found 432.6915.

Finally, we tackled the implementation of our **V**–amino acid hybrids into SPPS. For this purpose, we devised first the synthesis of the appropriate *N*-protected amino acid Alloc-**4·**2Cl, having an Alloc group, orthogonal to Fmoc, and that would enable the classic Fmoc/*t*-Bu SPPS strategy.^24^ To obtain Alloc-**4·**2Cl, we used a slight modification of the synthetic protocol explained above for Boc-**4**·2Cl (Scheme 1), with the introduction of the protecting group on intermediate **1**H·2Br just before the second alkylation of **B**_**P**_ (Figure 4a). In that manner, the targeted amino acid was successfully synthesized in an excellent 51% overall yield, simply by reaction of the aminoethylbipyridium **1**H·2Br with allyl chloroformate followed by the introduction of the corresponding 4-(chloromethyl)benzoic acid moiety. Consequently, by using the Fmoc/*t-*Bu strategy on a Rink amide resin, we followed with the SPPS of the model tripeptide Fmoc-l-Phe**-4-**l**-**Phe-NH_2_ (**9·**2TFA). As shown in Figure 4b, that was achieved by coupling of Fmoc-l-Phe-OH to the resin and subsequent deprotection, followed by introduction of the **V** moiety by coupling of Alloc-**4·**2Cl using HBTU/DIEA in DMF. Alloc deprotection using a slight modification of the classic Pd(PPh_3_)_4_/PhSiH_3_ method,^25^ immediately followed by the coupling of Fmoc-l-Phe-OH and cleavage from the solid support, led to tripeptide **9·**2TFA on a 6% overall yield after semipreparative HPLC purification. The associated ESI-MS spectrogram for the main peak on the HPLC chromatogram showed peaks at *m*/*z* = 963.3699 and *m*/*z* = 425.1915 corresponding to the loss of the trifluoroacetate anions on the expected structure (Figure 4c,d). Furthermore, the 1D/2D NMR experiments recorded in CD_3_CN/D_2_O for the purified reaction product showed data fully consistent with that observed for **7·**2TFA and expected for **9·**2TFA and allowed for a full assignment of the ^1^H/^13^C nuclei in the molecule. As in the case of **7·**2TFA, the introduction of the **V** moiety within the analogous tripeptide **9** can be easily inferred from the diagnostic resonances of the electroactive unit on the ^1^H NMR (Figure 4e).

**Figure 4:**
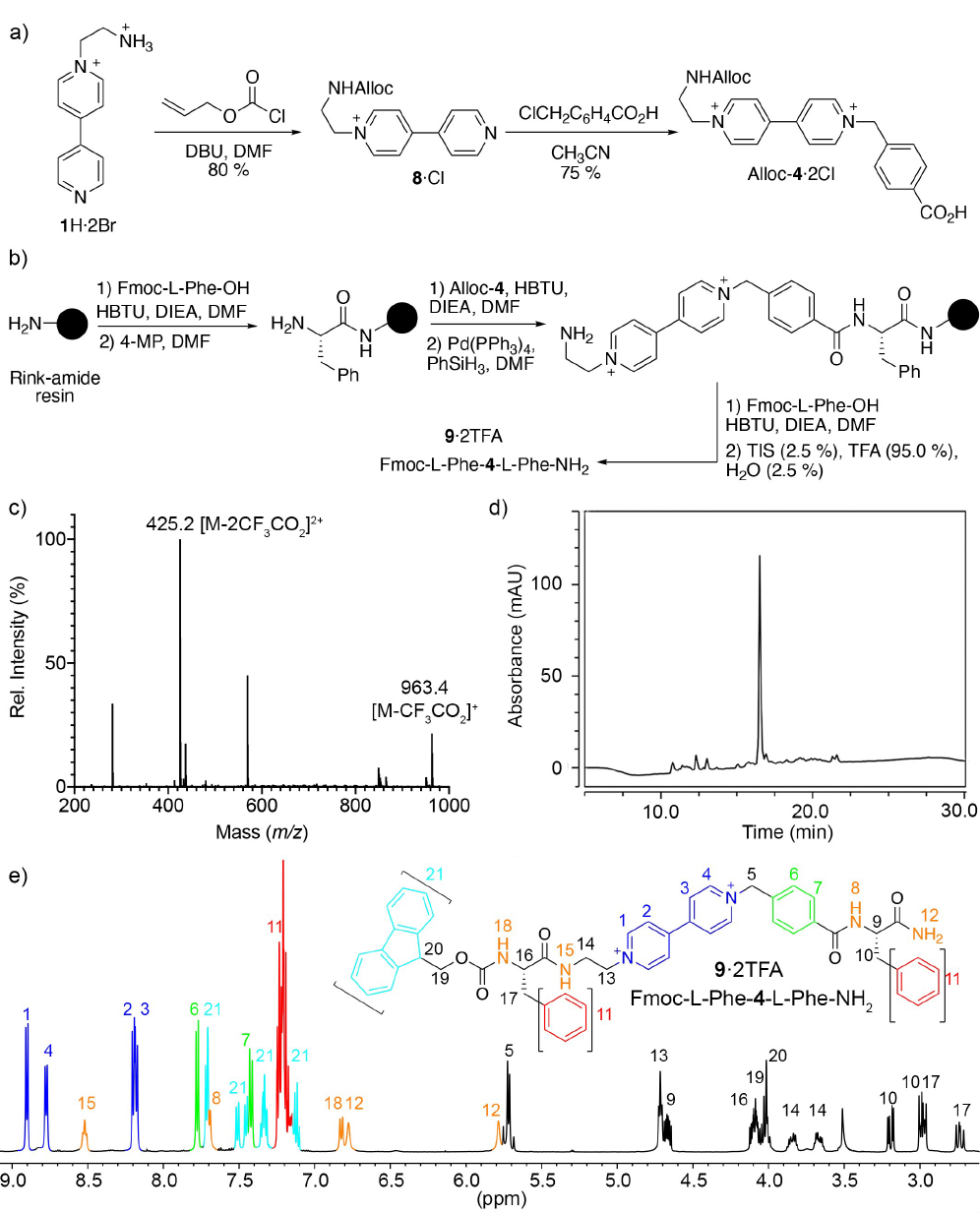
(a) Synthesis of the *N*-Alloc protected **V**–amino acid hybrid Alloc-**4**·2Cl. (b) SPPS of the model tripeptide **9·**2TFA. (c) MS spectrum corresponding to the peak at *t*_R_ = 16.6 min of the HPLC chromatogram at 220 nm (d) for **9·**2TFA. (e) Partial ^1^H NMR spectra (CD_3_CN, 500 MHz) for **9·**2TFA including the assignation based on 1D and 2D NMR data.

Finally, we tried to qualitatively assess the interaction between one of our model peptides (**7**·2TFA) and CB[8]. Hence, a 1 mM solution of **7·**2TFA, in D_2_O with 50 mM phosphate buffer solution at pD = 7, was saturated with CB[8] and the corresponding ^1^H NMR recorded after filtration of excess nondissolved macrocyle.^22^ Although the resulting complex pattern of broadened signals qualitatively imply an interaction between CB[8] and the amino acid–viologen hybrid, further extensive investigation would be needed in order to properly characterize this intricate system.^17^

In summary, we have described in this work the development of a series of viologen–amino acid hybrids that have been efficiently prepared both as “naked” or as *N*-protected derivatives suitable for L/SPPS. The ability of obtained amino acids as typical first guests of the cucurbituril family of hosts was explored for the unprotected derivative **2**H·3Cl, being found to behave in a similar fashion with both CB[7] and CB[8], as other simple viologen derivatives. Furthermore, we have corroborated the implementation of the *N*-protected derivatives Boc-**4·**2Cl and Alloc-**4·**2Cl on, respectively, the L and SPPS of a model tripeptide having the Phe-**V**-Phe sequence. Overall, these results expand considerably not only the toolbox for the synthesis of new (redox) stimuli-responsive peptide materials,^26^ but they open as well the door for the transient noncovalent modification of peptide structures by means of CB[8]-based heteroternary complexation,^27^ an area that we are currently exploring in our laboratories.

## Supporting Information Available

Experimental procedures, characterization data for new compounds, and titration experiments for CB[7,8]complexes (PDF)

## References

[ref1] Monk P. M. S. The Viologens: Physicochemical Properties, Synthesis, and Applications of the Salts of 4,4′-Bipyridine; John Wiley & Sons: Chichester, 1998.

[ref2] aStriepe L.; Baumgartner T. Viologens and Their Application as Functional Materials. Chem. - Eur. J. 2017, 23, 16924–16940. 10.1002/chem.201703348. 28815887

[ref3] aShah K. W.; Wang S.-X.; Soo D. X. Y.; Xu J. Viologen-Based Electrochromic Materials: From Small Molecules, Polymers and Composites to Their Applications. Polymers 2019, 11, 1839 10.3390/polym11111839. PMC6918392 31717323

[ref4] Liu W.; Lu W.; Zhang H.; Li X. Aqueous Flow Batteries: Research and Development. Chem. - Eur. J. 2019, 25, 1649–1664. 10.1002/chem.201802798. 30074285

[ref5] aHu B.; DeBruler C.; Rhodes Z.; Liu T. L. Long-Cycling Aqueous Organic Redox Flow Battery (AORFB) toward Sustainable and Safe Energy Storage. J. Am. Chem. Soc. 2017, 139, 1207–1214. 10.1021/jacs.6b10984. 27973765

[ref6] aDale E. J.; Vermeulen N. A.; Juricek M.; Barnes J. C.; Young R. M.; Wasielewski M. R.; Stoddart J. F. Supramolecular Explorations: Exhibiting the Extent of Extended Cationic Cyclophanes. Acc. Chem. Res. 2016, 49, 262–273. 10.1021/acs.accounts.5b00495. 26836816

[ref7] aAshwin B. C. M. A.; Shanmugavelan P.; Muthu Mareeswaran P. Electrochemical aspects of cyclodextrin, calixarene and cucurbituril inclusion complexes. J. Inclusion Phenom. Macrocyclic Chem. 2020, 98, 149–170. 10.1007/s10847-020-01028-4.

[ref8] Zhang M.; Yan X.; Huang F.; Niu Z.; Gibson H. W. Stimuli-Responsive Host–Guest Systems Based on the Recognition of Cryptands by Organic Guests. Acc. Chem. Res. 2014, 47, 1995–2005. 10.1021/ar500046r. 24804805

[ref9] Xue M.; Yang Y.; Chi X.; Zhang Z.; Huang F. Pillararenes, A New Class of Macrocycles for Supramolecular Chemistry. Acc. Chem. Res. 2012, 45, 1294–1308. 10.1021/ar2003418. 22551015

[ref10] Barrow S. J.; Kasera S.; Rowland M. J.; del Barrio J.; Scherman O. A. Chem. Rev. 2015, 115, 12320–12406. 10.1021/acs.chemrev.5b00341. 26566008

[ref11] Cucurbiturils and Related Macrocycles; Kim K., Ed.; The Royal Society of Chemistry, 2020.

[ref12] Pazos E.; Novo P.; Peinador C.; Kaifer A. E.; García M. D. Cucurbit[8]uril (CB[8])-Based Supramolecular Switches. Angew. Chem., Int. Ed. 2019, 58, 403–416. 10.1002/anie.201806575. 29978946

[ref13] Ko Y. H.; Kim E.; Hwang I.; Kim K. Supramolecular assemblies built with host-stabilized charge-transfer interactions. Chem. Commun. 2007, 13, 1305–1315. 10.1039/B615103E. 17377666

[ref14] aLiu Y.-H.; Zhang Y.-M.; Yu H.-J.; Liu Y. Cucurbituril-Based Biomacromolecular Assemblies. Angew. Chem., Int. Ed. 2021, 60, 3870–3880. 10.1002/anie.202009797. 32856749

[ref15] Neira I.; Blanco-Gómez A.; Quintela J. M.; García M. D.; Peinador C. Dissecting the “Blue Box”: Self-Assembly Strategies for the Construction of Multipurpose Polycationic Cyclophanes. Acc. Chem. Res. 2020, 53, 2336–2346. 10.1021/acs.accounts.0c00445. 32915539

[ref16] aSolid-phase Zincke reaction for the synthesis of peptide-4,4′-bipyridinium conjugates. Corton P.; Novo P.; López-Sobrado V.; García M. D.; Peinador C.; Pazos E. Synthesis 2020, 52, 537–543. 10.1055/s-0039-1690016.

[ref17] aOh K. J.; Cash K. J.; Hugenberg V.; Plaxco K. W. Peptide Beacons: A New Design for Polypeptide-Based Optical Biosensors. Bioconjugate Chem. 2007, 18, 607–609. 10.1021/bc060319u. PMC2528055 17461545

[ref18] Gavin J. A.; García M. E.; Benesi A. J.; Mallouk T. E. Chiral Molecular Recognition in a Tripeptide Benzylviologen Cyclophane Host.. J. Org. Chem. 1998, 63, 7663–7669. 10.1021/jo980352c.

[ref19] Domarco O.; Neira I.; Rama T.; Blanco-Gómez A.; García M. D.; Peinador C.; Quintela J. M. Synthesis of non-symmetric viologen-containing ditopic ligands and their Pd(ii)/Pt(ii)-directed self-assembly. Org. Biomol. Chem. 2017, 15, 3594–3602. 10.1039/C7OB00161D. 28271117

[ref20] aYang C.; Wang M.-S.; Cai L.-Z.; Jiang X.-M.; Wu M.; Guo G.-C.; Huang J.-S. Crystal structures and visible-light excited photoluminescence of *N*-methyl-4,4′-bipyridinium chloride and its Zn(II) and Cd(II) complexes. Inorg. Chem. Commun. 2010, 13, 1021–1024. 10.1016/j.inoche.2010.05.020.

[ref21] Neira I.; García M. D.; Peinador C.; Kaifer A. E. Terminal Carboxylate Effects on the Thermodynamics and Kinetics of Cucurbit[7]uril Binding to Guests Containing a Central Bis(Pyridinium)-Xylylene Site. J. Org. Chem. 2019, 84, 2325–2329. 10.1021/acs.joc.8b02993. 30652476

[ref22] See the Supporting Information.

[ref23] Ramalingam V.; Urbach A. R. Cucurbit[8]uril Rotaxanes. Org. Lett. 2011, 13, 4898–4901. 10.1021/ol201991e. 21846094

[ref24] Isidro-Llobet A.; Álvarez M.; Albericio F. Amino Acid-Protecting Groups. Chem. Rev. 2009, 109, 2455–2504. 10.1021/cr800323s. 19364121

[ref25] Thieriet N.; Alsina J.; Giralt E.; Guibe F.; Albericio F. Use of Alloc-amino acids in solid-phase peptide synthesis. Tandem deprotection-coupling reactions using neutral conditions. Tetrahedron Lett. 1997, 38, 7275–7278. 10.1016/S0040-4039(97)01690-0.

[ref26] aMart R. J.; Osborne R. D.; Stevens M. M.; Ulijn R. V. Peptide-based stimuli-responsive biomaterials. Soft Matter 2006, 2, 822–835. 10.1039/b607706d. 32680274

[ref27] Hou C.; Huang Z.; Fang Y.; Liu J. Construction of protein assemblies by host-guest interactions with cucurbiturils. Org. Biomol. Chem. 2017, 15, 4272–4281. 10.1039/C7OB00686A. 28443929

