# Supplementary material for: Amino Acid–Viologen Hybrids: Synthesis, Cucurbituril Host–Guest Chemistry, and Implementation on the Production of Peptides
Source: J Org Chem. 2021 Dec 10;87(1):760–4. doi: 10.1021/acs.joc.1c02040 (PMC8749954; doi:10.1021/acs.joc.1c02040)
Supplement: Supplementary file 1 — jo1c02040_si_001.pdf [file jo1c02040_si_001.pdf]

Supporting Information

Amino acid-viologen hybrids: synthesis, cucurbituril host-guest chemistry and implementation on the production of peptides.

Liliana Barravechia,[a] Iago Neira,[a] Elena Pazos,[a] Carlos Peinador,[a,*] and Marcos D. García,[a,*].

Departamento de Química and Centro de Investigaciones Científicas Avanzadas (CICA). Facultad de Ciencias, Universidade da Coruña, 15071, A Coruña, Spain.

*E-mail:carlos.peinador@udc.es.
*E-mail:marcos.garcia1@udc.es.

Index of Figures:

Figure S1: ^{1}H NMR (500 MHz, D_2O) spectrum of 1H·2Br. S13
Figure S2: $^{13}C\{^{1}H\}$ NMR (125 MHz, D_2O) spectrum of 1H·2Br. S13
Figure S3: $^{13}C\{^{1}H\}$ NMR and DEPT (125 MHz, D_2O) spectrum of 1H·2Br. S14
Figure S4: COSY (500 MHz, D_2O) spectrum of 1H·2Br. S14
Figure S5: HSQC (500 MHz, D_2O) spectrum of 1H·2Br. S15
Figure S6: HMBC (500 MHz, D_2O) spectrum of 1H·2Br. S15
Figure S7: ^{1}H NMR (500 MHz, D_2O) spectrum of E·3Br. S16
Figure S8: $^{13}C\{^{1}H\}$ NMR (125 MHz, D_2O) spectrum of E·3Br. S16
Figure S9: $^{13}C\{^{1}H\}$ NMR and DEPT (125 MHz, D_2O) spectrum of E·3Br. S17
Figure S10: COSY (500 MHz, D_2O) spectrum of E·3Br. S17
Figure S11: HSQC (500 MHz, D_2O) spectrum of E·3Br. S18
Figure S12: HMBC (500 MHz, D_2O) spectrum of E·3Br. S18
Figure S13: UV-Vis spectra of E·3Br in 50mM phosphate buffer solution, pH=7 from 7.4 μM to 43.0 μM. S19
Figure S14: Linear relationship between the absorbance at 272 nm and the concentration of E^{+3} where ε=23662 $Lmol^{-1}cm^{-1}$. S19
Figure S15: ^{1}H NMR (500 MHz, D_2O) spectrum of 2H·3Cl. S20
Figure S16: $^{13}C\{^{1}H\}$ NMR (125 MHz, D_2O) spectrum of 2H·3Cl. S20
Figure S17: $^{13}C\{^{1}H\}$ NMR and DEPT (125 MHz, D_2O) spectrum of 2H·3Cl. S21
Figure S18: COSY (500 MHz, D_2O) spectrum of 2H·3Cl. S21
Figure S19: HSQC (500 MHz, D_2O) spectrum of 2H·3Cl. S22
Figure S20: HMBC (500 MHz, D_2O) spectrum of 2H·3Cl. S22
Figure S21: UV-Vis spectra of 2H·3Br in 50 mM phosphate buffer solution, pH 7 from 9.9 μM to 56.6 μM. S23
Figure S22: Linear relationship between the absorbance at 272 nm and the concentration of $2H^{+3}$ where ε=13835 $Lmol^{-1}cm^{-1}$. S23
Figure S23: ^{1}H NMR (400 MHz, D_2O, pD=7) spectrum of a 1mM 2H·3Cl, CB8, and 2,7-DHN mixture. S24
Figure S24: T. Var. (400 MHz, D_2O, pD=7) spectrum of a 1mM 2H·3Cl, CB8, and 2,7-DHN mixture at: a) 348.15 K b) 345.15 K c) 338.15 K d) 328.15 K e) 318.15 K f) 308.15 K g) 298.15 K. S24
Figure S25: ^{1}H NMR (500 MHz, D_2O) spectrum of 3·Cl. S25
Figure S26: $^{13}C\{^{1}H\}$ NMR (125 MHz, D_2O) spectrum of 3·Cl. S25
Figure S27: $^{13}C\{^{1}H\}$ NMR and DEPT (125 MHz, D_2O) spectrum of 3·Cl. S26
Figure S28: COSY (500 MHz, D_2O) spectrum of 3·Cl. S26
Figure S29: HSQC (500 MHz, D_2O) spectrum of 3·Cl. S27
Figure S30: HMBC (500 MHz, D_2O) spectrum of 3·Cl. S27
Figure S31: ^{1}H NMR (500 MHz, D_2O) spectrum of Boc-4·2Cl. S28
Figure S32: $^{13}C\{^{1}H\}$ NMR (125 MHz, D_2O) spectrum of Boc-4·2Cl. S28
Figure S33: $^{13}C\{^{1}H\}$ NMR and DEPT (125 MHz, D_2O) spectrum of Boc-4·2Cl. S29
Figure S34: COSY (500 MHz, D_2O) spectrum of Boc-4·2Cl. S29
Figure S35: HSQC (500 MHz, D_2O) spectrum of Boc-4·2Cl. S30
Figure S36: HMBC (500 MHz, D_2O) spectrum of Boc-4·2Cl. S30
Figure S37: ^{1}H NMR (500 MHz, D_2O) spectrum of 5·2Cl. S31
Figure S38: $^{13}C\{^{1}H\}$ NMR (125 MHz, D_2O) spectrum of 5·2Cl. S31

Figure S39: $^{13}C\{^1H\}$ NMR and DEPT (125 MHz, D_2O) spectrum of 5·2Cl....................................S32
Figure S40: COSY (500 MHz, D_2O) spectrum of 5·2Cl. S32
Figure S41: HSQC (500 MHz, D_2O) spectrum of 5·2Cl...S33
Figure S42: HMBC (500 MHz, D_2O) spectrum of 5·2Cl..S33
Figure S43: ^1H-NMR (500 MHz, CD_3CN) assignment of 7·2TFA. S34
Figure S44: $^{13}C\{^1H\}$ NMR (125 MHz, CD_3CN) spectrum of 7·2TFA...S34
Figure S45: $^{13}C\{^1H\}$ NMR and DEPT (125 MHz, CD_3CN) spectrum of 7·2TFA.............................S35
Figure S46: COSY (500 MHz, CD_3CN) spectrum of 7·2TFA. S35
Figure S47: HSQC (500 MHz, CD_3CN) spectrum of 7·2TFA...S36
Figure S48: HMBC (500 MHz, CD_3CN) spectrum of 7·2TFA. S36
Figure S49: ^1H NMR (500 MHz, CD_3CN) spectrum of 8·Cl..S37
Figure S50: $^{13}C\{^1H\}$ NMR (125 MHz, CD_3CN) spectrum of 8·Cl..S37
Figure S51: $^{13}C\{^1H\}$ NMR and DEPT (125 MHz, CD_3CN) spectrum of 8·Cl.................................S38
Figure S52: COSY (500 MHz, CD_3CN) spectrum of 8·Cl. S38
Figure S53: HSQC (500 MHz, CD_3CN) spectrum of 8·Cl..S39
Figure S54: HMBC (500 MHz, CD_3CN) spectrum of 8·Cl...S39
Figure S55: ^1H NMR (500 MHz, D_2O) spectrum of Alloc-4·2TFA. S40
Figure S56: $^{13}C\{^1H\}$ NMR (125 MHz, D_2O) spectrum of Alloc-4·2TFA...S40
Figure S57: $^{13}C\{^1H\}$ NMR and DEPT (125 MHz, D_2O) spectrum of Alloc-4·2TFA.........................S41
Figure S58: COSY (500 MHz, D_2O) spectrum of Alloc-4·2TFA. S41
Figure S59: HSQC (500 MHz, D_2O) spectrum of Alloc-4·2TFA..S42
Figure S60: HMBC (500 MHz, D_2O) spectrum of Alloc-4·2TFA...S42
Figure S61: UV-Vis spectra of Alloc-4·2Cl in 50mM buffer solution, pH 7 from 7.4 µM to 43.0 µM. S43
Figure S62: Linear relationship between the absorbance at 259 nm and the concentration of Alloc-4^{+2} where ε=16174 $Lmol^{-1}cm^{-1}$. S43
Figure S63: ^1H NMR (500 MHz, CD_3CN) spectrum of 9·2TFA. S44
Figure S64: ^1H-NMR (500 MHz, CD_3CN) assignment of 9·2TFA. S44
Figure S65: $^{13}C\{^1H\}$ NMR (125 MHz, CD_3CN) spectrum of 9·2TFA...S45
Figure S66: $^{13}C\{^1H\}$ NMR and DEPT (125 MHz, CD_3CN) spectrum of 9·2TFA.............................S45
Figure S67: COSY (500 MHz, CD_3CN) spectrum of 9·2TFA. S46
Figure S68: HSQC (500 MHz, CD_3CN) spectrum of 9·2TFA...S46
Figure S69: HMBC (500 MHz, CD_3CN) spectrum of 9·2TFA. S47
Figure S70: ^1H NMR (500 MHz, D_2O) spectrum of 2H⊂CB[7]..S47
Figure S71: Partial NMR ^1H (500 MHZ, D_2O, pD=7) of: a) 2H·3Cl b) 2H·3Cl + 0.5 eq CB[7] c) 2H·3Cl + 1 eq CB[7] d) 2H·3Cl + 2 eq CB[7] e) 2H·3Cl + 3 eq CB[7]. S48
Figure S72: ^1H NMR (500 MHz, D_2O) spectrum of 2H⊂CB[8]..S48
Figure S73: Partial NMR ^1H (500 MHZ, D_2O, pD=7) of: a) 2H·3Cl b) 2H·3Cl + excess of CB[8]. .S49
Figure S74: T. Var. (400 MHz, D_2O, pD=7) spectrum of a mixture of 2H·3Cl, CB[8], and 2,7-DHN at: a) 343.15 K b) 333.15 K c) 323.15 K d) 313.15 K e) 303.15 K f) 298.15 K............................S49
Figure S75: COSY (400 MHz, D_2O, T=343.25 K) spectrum of a mixture of 2H·3Cl, CB[8], and 2,7-DHN. S50
Figure S76. Partial NMR ^1H (500 MHZ, D_2O, pD=7) of: a) 7·2TFA b) 7·2TFA + excess of CB[8]. .S51

Synthetic procedures:

SP1: Synthesis and characterization of 1-(2-ammonioethyl)-[4,4'-bipyridin]-1-ium bromide **1H**·2Br.

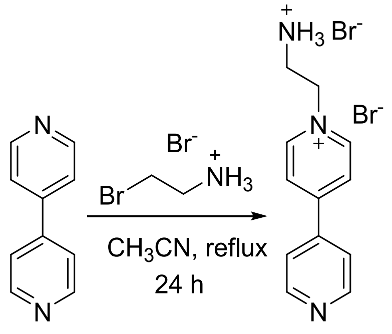

1H·2Br

To a solution of 4,4'-bipyridine (3.43 g, 21.9 mmol) in 30 mL of acetonitrile 2-bromoethan-1-aminium (3.00 g, 14.6 mmol) was added. The resulting mixture was refluxed with heating mantle under stirring for 24 hours. The yellow precipitate formed was filtered, washed with hot acetonitrile (3 × 30 mL), and dried under vacuum to afford compound **1H**·2Br as a yellowish solid (4.82 g, 92%).

1H·2Br: ^{1}H NMR (500 MHz, D_2O): δ 9.08 (d, J = 6.5 Hz, 2H), 8.80 (d, J = 6.2 Hz, 2H), 8.52 (d, J = 6.5 Hz, 2H), 7.95 (d, J = 6.1 Hz, 2H), 5.05 (t, J = 6.6 Hz, 2H), 3.77 (t, J = 6.6 Hz, 2H). ^{13}C{^{1}H} NMR: (125 MHz, D_2O): δ 155.1 (C), 149.8 (CH), 145.3 (CH), 142.4 (C), 126.6 (CH), 122.5 (CH), 57.6 (CH_2), 39.1 (CH_2) ppm. HRMS (ESI) (*m/z*): calcd for $[C_{12}H_{14}N_3]^+$ 200.1190, found 200.1182.

SP2: Synthesis and characterization of 1-(2-ammonioethyl)-1'-(2-ethoxy-2-oxoethyl)-[4,4'-bipyridine]-1,1'-diium **E**·3Br.

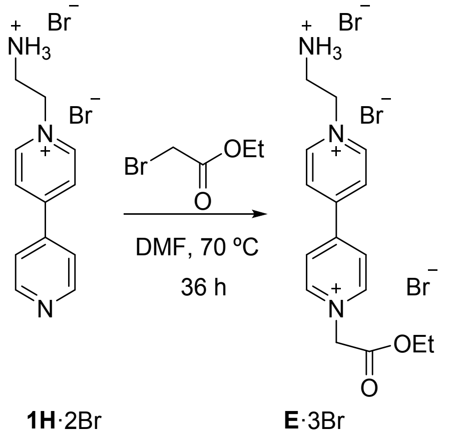

1H·2Br **E**·3Br

To a solution of **1H**·2Br (340 mg, 0.95 mmol) in 20 mL of DMF ethyl 2-bromoacetate (210 µL, 1.90 mmol) was added. The resulting mixture was refluxed with heating mantle under stirring for 36 hours. The yellow precipitate formed was filtered, washed with ether (2x 20 mL), and dried under vacuum to afford compound **E**·3Br as a yellowish solid (273 mg, 55%).

E·3Br: ^{1}H NMR (500 MHz, D_2O): δ 9.27 (d, J = 5.8 Hz, 2H), 9.15 (d, J = 6.0 Hz, 2H), 8.69 (d, J = 5.7 Hz, 2H), 8.66 (d, J = 5.9 Hz, 2H), 5.72 (s, 2H), 5.14 (t, J = 6.5 Hz, 2H), 4.37 (c, J = 7.2 Hz, 2H), 3.81 (t, J = 6.7 Hz, 2H), 1.32 (t, J = 7.2 Hz, 3H). $^{13}C\{^{1}H\}$ NMR: (125 MHz, D_2O): δ 166.8 (C), 151.1 (C), 150.9 (C), 147.0 (CH), 146.2 (CH), 127.7 (CH), 127.0 (CH), 64.2 (CH_2), 61.1 (CH_2), 58.1 (CH_2), 39.0 (CH_2), 13.1 (CH_3) ppm. HRMS (ESI) (m/z): calcd for $[C_{16}H_{20}N_3O_2]^+$ 286.1551, found 286.1551.

SP3: Synthesis and characterization of 1-(2-ammonioethyl)-1'-(carboxymethyl)-[4,4'-bipyridine]-1,1'-diium **2H**·3Cl.

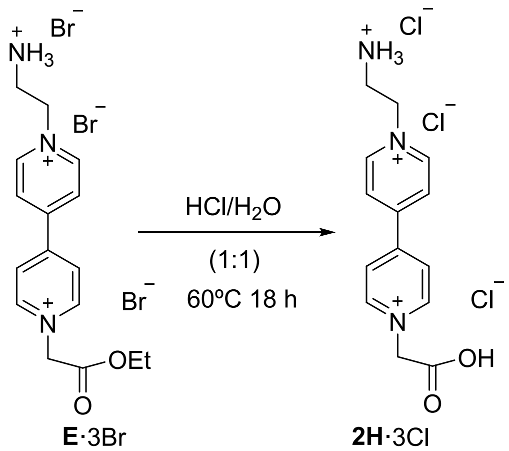

Compound **E**·3Br (200 mg, 0.38 mmol) was dissolved in 15 mL of a 1:1 mixture of HCl (37 %) and water. The resulting mixture was stirred at 60°C with heating mantle for 18 hours. The solvent was removed under reduced pressure to leave a solid residue that was dried under vacuum to afford compound **2H**·3Cl as a yellowish solid (132 mg, 95 %).

2H·3Cl: ^{1}H NMR (500 MHz, D_2O): δ 9.27 (d, J = 7.0 Hz, 2H), 9.10 (d, J = 7.0 Hz, 2H), 8.70 (d, J = 6.9 Hz, 2H), 8.62 (d, J = 7.0 Hz, 2H), 5.46 (s, 2H), 5.16 (t, J = 6.6 Hz, 2H), 3.84 (t, J = 6.6 Hz, 2H). $^{13}C\{^{1}H\}$ NMR: (125 MHz, D2O): δ 169.0 (C), 158.2 (C), 150.5 (C), 150.3 (CH), 146.7 (C), 145.9 (CH), 132.4 (CH), 126.9 (CH), 126.7 (CH), 117.1 (CH), 65.8 (CH_2), 61.9 (CH_2), 61.9 (CH_2), 40.9 (CH_2) ppm. HRMS (ESI) (m/z): calcd for $[C_{14}H_{17}N_3O_2]^{+3}$ 259.1304, found 259.1290.

SP4: Synthesis and characterization of 1-(2-((*tert*-butoxycarbonyl)amino)ethyl)-[4,4'-bipyridin]-1-ium chloride **3**·Cl:

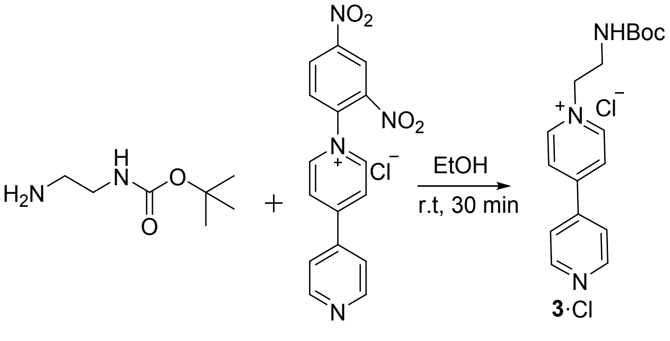

A solution of 1-(2,4-dinitrophenyl)-[4,4'-bipyridin]-1-ium (1.17 g, 3.26 mmol) and *tert*-butyl (2-aminoethyl)carbamate[1] (0.78 g, 4.88 mmol) in 30 mL of EtOH was stirred at room temperature for 30 minutes. Then, the solvent was removed under reduced pressure and the crude was dissolved in 80 mL of a (1:1) H_2O/EtOAc mixture. The organic phase was separated from the aqueous phase and this one was extracted again with EtOAc (3 × 40 mL). The product-containing aqueous fractions were combined, and the solvent was evaporated under vacuum to afford compound **3**·Cl as a brown solid (812 mg, 70 %).

3·Cl: ^1H NMR (500 MHz, D_2O): δ 8.98 (d, J = 6.4 Hz, 2H), 8.78 (d, J = 5.6 Hz, 2H), 8.44 (d, J = 6.2 Hz, 2H), 7.90 (d, J = 5.5 Hz, 2H), 4.73 (m, 2H), 3.72 (t, J = 5.3 Hz, 2H), 1.24 (s, 9H). $^{13}C\{^1H\}$ NMR: (125 MHz, D2O): δ 157.5 (C), 152.3 (C), 150.0 (CH), 145.3 (CH), 142.4 (C), 125.9 (CH), 122.4 (CH), 81.2 (C), 61.6 (CH_2), 40.3 (CH_2), 27.3 (CH_3) ppm. HRMS (ESI) (*m/z*): calcd for $[C_{17}H_{22}N_3O_2]^+$ 300.1706, found 300.1713.

SP5: Synthesis and characterization of 1-(2-((*tert*-butoxycarbonyl)amino)ethyl)-1'-(4-carboxybenzyl)-[4,4'-bipyridine]-1,1'-diium dichloride Boc-**4**·2Cl:

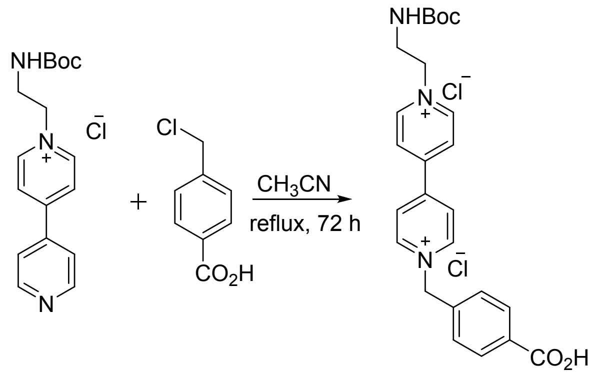

3·Cl Boc-**4**·2Cl

To a solution of **3**·Cl (300 mg, 0.90 mmol) in 30 mL of acetonitrile 4-(chloromethyl)benzoic acid (304 mg, 1.80 mmol) was added. The resulting mixture was refluxed with heating mantle under stirring for 72 hours. The brown precipitate formed was filtered, washed with hot acetonitrile (2 × 25 mL), and dried under vacuum to afford Boc-**4**·2Cl as a brown solid (312 mg, 70%).

Boc-**4**·2Cl: ^1H NMR (500 MHz, D_2O): δ 9.17 (d, J = 6.4 Hz, 2H), 9.12 (d, J = 6.3 Hz, 2H), 8.55 (d, J = 6.7 Hz, 2H), 8.52 (d, J = 6.5 Hz, 2H), 8.05 (d, J = 8.4 Hz, 2H), 7.57 (d, J = 8.3 Hz, 2H), 6.00 (s, 2H), 4.70 (overlap with solvent, 2H), 3.71 (t, J = 5.3 Hz, 2H), 1.22 (s, 9H). $^{13}C\{^1H\}$ NMR: (125 MHz, D_2O): δ 170.9 (C), 157.6 (C), 150.3 (C), 150.2 (C), 146.0 (CH), 145.7 (CH), 136.6 (C), 132.8 (C), 130.4 (CH), 129.1 (CH), 127.1 (CH), 126.8 (CH), 81.2 (C), 64.1 (CH_2), 62.2 (CH_2), 40.5 (CH_2), 27.2 (CH_3) ppm. HRMS (ESI) (*m/z*): calcd for $[C_{25}H_{28}N_3O_4]^+$ 434.2071, found 434.2069.

[1] *Tert*-butyl(2-aminoethyl)carbamate was prepared according to literature procedures with the spectroscopy data in good agreement with the previously reported by Wu, G.; Zeng, F.; Yu, C.; Wu, S.; Li, W. *J. Mater. Chem. B*. **2014**, *2*, 8528.

SP6: Synthesis and characterization of (*R*)-1-(2-((*tert*-butoxycarbonyl)amino)ethyl)-1'-(4-((1-methoxy-1-oxo-3-phenylpropan-2-yl)carbamoyl)benzyl)-[4,4'-bipyridine]-1,1'-diium **5**·2Cl:

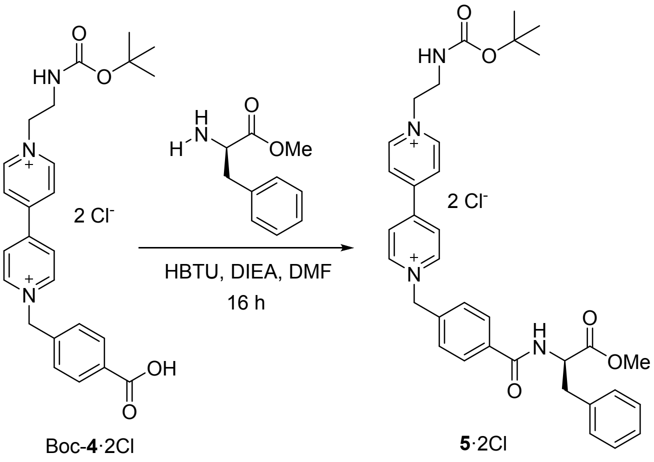

D-H-Phe-(OMe) (64.7 mg, 0.30 mmol) was dissolved in dry DMF (10 mL) in a 50 mL two-necked round-bottomed flask under argon. Then, compound Boc-**4**·2Cl (100 mg, 0.20 mmol), HBTU (114 mg, 0.30 mmol) and DIEA (69.0 µL, 0.40 mmol) were dissolved in dry DMF (5 mL). The resulting mixture was added dropwise to the D-H-Phe-(OMe) solution and stirred at room temperature overnight. The solvent was evaporated under reduced pressure to leave a solid residue. The solid residue was subjected to flash chromatography (SiO_2) using two different eluent phases: 1:1 DCM/MeOH to remove impurities and (4/1/1) CH_3CN/(0.6 M) NaCl/MeOH to elute the compound. The product-containing fractions were combined and the solvents evaporated. The residue was suspended in EtOH and filtered off to remove NaCl. The EtOH was removed under reduced pressure to afford **5**·Cl as a brown oil (78.0 mg, 60 %).

5·2Cl: ^{1}H NMR (500 MHz, D_2O): δ 9.16 (d, *J* = 6.6 Hz, 2H), 9.13 (d, *J* = 6.5 Hz, 2H), 8.56 (d, *J* = 6.4 Hz, 2H), 8.52 (d, *J* = 6.4 Hz, 2H), 7.69 (d, *J* = 8.1 Hz, 2H), 7.56 (d, *J* = 8.2 Hz, 2H), 7.25 (m, 5H), 5.97 (s, 2H), 4.85 (m, 2H), 3.72 (t, *J* = 5.2 Hz, 2H), 3.30 (dd, *J* = 13.9, 5.5 Hz, 1H), 3.07 (dd, *J* = 13.9, 5.7 Hz, 1H), 1.23 (s, 9H). ^{13}C{^{1}H} NMR: (125 MHz, D_2O): δ 173.4 (C), 169.7 (C), 157.6 (C), 150.2 (C), 150.1 (C), 146.0 (CH), 145.7 (CH), 136.5 (C), 136.1 (C), 134.3 (C), 129.3 (CH), 129.1 (CH), 128.6 (CH), 128.3 (CH), 127.1 (CH), 127.0 (CH), 126.9 (CH), 81.2 (C), 64.0 (CH_2), 62.2 (CH_2), 54.6 (CH), 52.9 (CH_3), 40.5 (CH_2), 36.4 (CH_2), 27.3 (CH_3) ppm. HRMS (ESI) (*m/z*): calcd for $[C_{35}H_{40}N_4O_5]^{+2}$ 298.1494, found 298.1493.

SP7: Synthesis and characterization of (*R*)-1-(2-aminoethyl)-1'-(4-((1-methoxy-1-oxo-3-phenylpropan-2-yl)carbamoyl)benzyl)-[4,4'-bipyridine]-1,1'-diium **6**·3TFA:

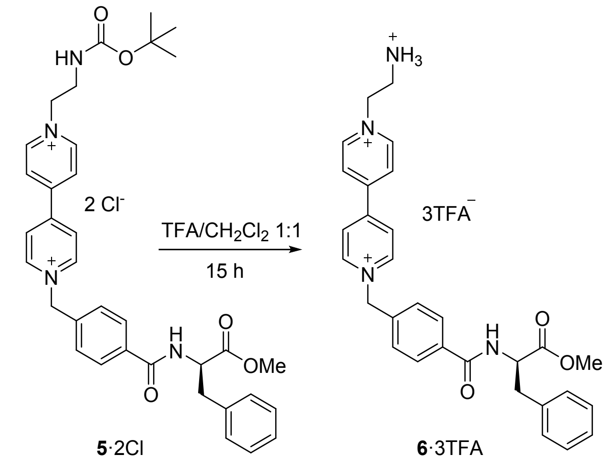

Compound **5**·2Cl was dissolved in 15 mL of a 1:1 TFA and CH_2Cl_2 mixture and stirred at room temperature for 15 hours. The solvent was removed under reduced pressure to leave a solid residue that was dried under vacuum to afford **6**·3TFA as a brown oil. The solid residue obtained was used in the next step without any further purification.

SP8: Synthesis and characterization of peptide **7**·2TFA (Fmoc-Phe-**4**-D-Phe-OMe·2TFA):

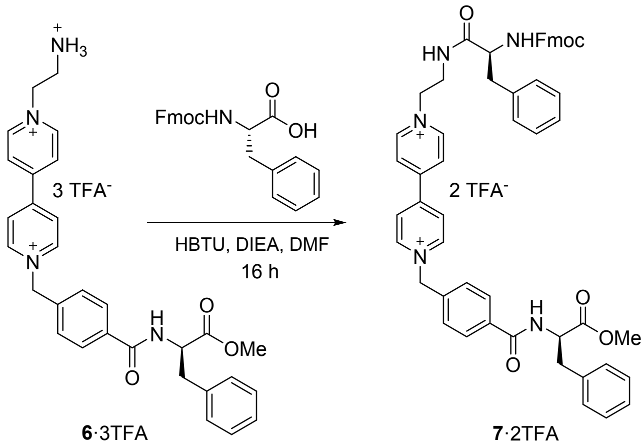

The solid residue **6**·3TFA (78.0 mg, 0.12 mmol) was dissolved in DMF (10 mL) in a 50 mL two-necked round-bottomed flask under argon. Then, Fmoc-Phe-OH (69.0 mg mg, 0.18 mmol), HBTU (68.2 mg, 0.18 mmol) and DIEA (41.5 µL, 0.24 mmol) were dissolved in dry DMF (10 mL). The resulting mixture was added dropwise to the solution of **6**·3TFA and stirred at room temperature overnight. The solvent was evaporated under reduced pressure to leave a solid residue. The solid residue was subjected to flash chromatography (SiO_2) using two different eluent phases: 1:1 DCM/MeOH to remove impurities and (4/1/1) CH_3CN/(0.6 M) NaCl/MeOH to elute the compound. The product-containing fractions were combined and the solvent evaporated. The residue was suspended in EtOH and filtered off to remove NaCl. The EtOH was removed under reduced pressure to afford **7**·2TFA as a yellowish solid (75.6 mg, 58 %).

7·2TFA: ^1H NMR (500 MHz, CD_3CN): δ 9.00 (d, J = 6.4 Hz, 2H), 8.88 (d, J = 6.3 Hz, 2H), 8.71 (s, 1H), 8.28 (dd, J = 11.6, 6.3 Hz, 4H), 7.85 (d, J = 8.2 Hz, 2H), 7.79 (d, J = 7.6 Hz, 2H), 7.60 (d,

J = 7.5 Hz, 2H), 7.53 (m, 3H), 7.49 (d, *J* = 8.0 Hz, 1H), 7.42 (m, 3H), 7.30 (m, 10H), 7.23 (m, 2H), 6.97 (d, 1H), 5.82 (d, *J* = 6.3 Hz, 2H), 4.87 (m, 1H), 4.81 (t, *J* = 5.4 Hz, 2H), 4.15 (m, 5H), 3.93 (m, 1H), 3.75 (m, 1H), 3.71 (s, 3H), 3.30 (dd, *J* = 13.9, 5.5 Hz, 1H), 3.14 (dd, *J* = 13.9, 9.1 Hz, 1H), 3.07 (dd, *J* = 13.9, 4.4 Hz. 1H), 2.82 (dd, *J* = 13.8, 10.7 Hz, 1H). $^{13}C\{^1H\}$ NMR: (125 MHz, CD_3CN): δ 173.9 (C), 172.8 (C), 166.8 (C), 157.0 (C), 151.1 (C), 150.2 (C), 147.2 (CH), 146.5 (CH), 144.9 (C), 144.7 (C), 141.8 (C), 138.8 (C), 138.2 (C), 136.7 (C), 130.3 (C), 130.1 (CH), 129.3 (CH), 129.2 (CH), 129.1 (CH), 128.7 (CH), 128.2 (CH), 128.1 (CH), 127.6 (CH), 127.5 (CH), 127.4 (CH), 126.4 (CH), 126.2 (CH), 120.9 (CH), 120.9 (CH), 67.2 (CH_2), 64.8 (CH_2), 62.0 (CH_2), 58.2 (CH), 55.1 (CH), 52.8 (CH_3), 47.7 (CH), 40.3 (CH_2), 38.3 (CH_2), 37.7 (CH_2) ppm. HRMS (ESI) (*m/z*): calcd for $[C_{54}H_{51}N_5O_6]^{+2}$ 432.6914, found 432.6915.

SP9: Synthesis and characterization of 1-(2-(((allyloxy)carbonyl)amino)ethyl)-[4,4'-bipyridin]-1-ium **8**·Cl.

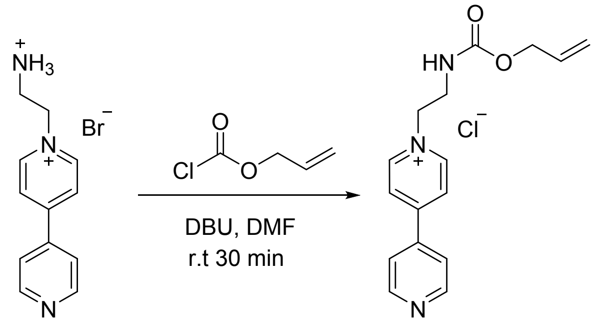

1H·2Br

8·Cl

Compound **1H**·2Br (2.00 g, 5.57 mmol) was dissolved in dry DMF (15 mL) in a 50 mL two-necked round-bottomed flask under argon. DBU (1,8-Diazabicyclo[5.4.0]undec-7-ene) (1.36 mL, 16.7 mmol) was added to the solution and the mixture was stirred at room temperature for 10 minutes. Then allyl chloroformate (1.78 mL, 16.7 mmol) was added dropwise. The brown precipitate formed was filtered and washed with dichloromethane (2 × 25 mL). The solid residue was subjected to flash chromatography (SiO_2, 4/1/1 CH_3CN/(0.6 M) NaCl/MeOH). The product-containing fractions were combined and the solvent evaporated. The residue was suspended in EtOH and filtered off to remove NaCl. The EtOH was removed under reduced pressure to afford **8**·Cl as an orange oil (1.60 g, 80 %).

8·Cl: ^1H NMR (500 MHz, CD_3CN): δ 8.84 (d, *J* = 6.2 Hz, 2H), 8.75 (d, *J* = 7.0 Hz, 2H), 8.31 (d, *J* = 6.9 Hz, 2H), 7.79 (d, *J* = 6.1 Hz, 2H), 5.83 (m, 1H), 5.60 (bs, 1H), 5.21 (dd, *J* = 17.5, 1.9 Hz, 2H), 5.14 (m, 2H), 4.62 (t, *J* = 5.4 Hz, 2H), 4.40 (d, *J* = 5.5 Hz, 2H), 3.67 (m, 2H). $^{13}C\{^1H\}$ NMR: (125 MHz, CD_3CN): δ 157.4 (C), 155.2 (C), 152.1 (CH), 146.3 (CH), 142.0 (C), 134.1 (CH), 126.7 (CH), 122.7 (CH), 117.7 (CH_2), 66.2 (CH_2), 62.2 (CH_2), 41.9 (CH_2) ppm. HRMS (ESI) (*m/z*): calcd for $[C_{16}H_{18}N_3O_2]^+$ 284.1394, found 284.1395.

SP10: Synthesis and characterization of 1-(2-(((allyloxy)carbonyl)amino)ethyl)-1'-(4-carboxybenzyl)-[4,4'-bipyridine]-1,1'-diium Alloc-**4**·2Cl.

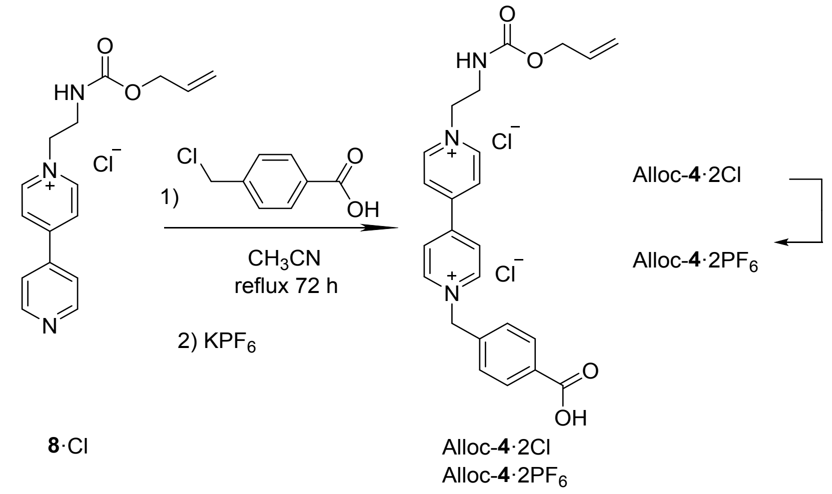

To a solution of **8**·Cl (1.00 g, 3.10 mmol) in 30 mL of acetonitrile 4-(chloromethyl)benzoic acid (1.06 g, 6.23 mmol) was added. The resulting mixture was refluxed with heating mantle under stirring for 72 hours. The brown precipitate formed was filtered, washed with hot acetonitrile (2 × 25 mL), and dried under vacuum to afford Alloc-**4**·2Cl as a brown solid (1.50 g, 75 %).

To a solution of Alloc-**4**·2Cl (257 mg, 0.52 mmol) in H_2O (30 mL), KPF_6 was added until no more precipitation was observed. The brown precipitate formed was filtered, washed with H_2O (20 mL) and dried under vacuum to afford Alloc-**4**·2PF_6 as a brown solid (334 mg, 90%).

Alloc-**4**·2TFA: ^{1}H NMR (500 MHz, D_2O): *δ* 9.15 (d, *J* = 6.4 Hz, 2H), 9.09 (d, *J* = 7.0 Hz, 2H), 8.51 (d, *J* = 6.7 Hz, 2H), 8.48 (d, *J* = 6.7 Hz, 2H), 8.04 (d, *J* = 8.3 Hz, 2H), 7.58 (d, *J* = 8.4 Hz, 2H), 6.92 (m, 1H), 5.96 (s, 2H), 5.85 (ddt, *J* = 16.2, 10.6, 5.4 Hz, 1H), 5.22 (dd, *J* = 17.4, 1.9 Hz, 1H), 5.15 (dd, *J* = 10.4, 1.7 Hz, 2H), 4.80 (t, *J* = 5.4 Hz, 2H), 4.41 (d, *J* = 5.5 Hz, 2H), 3.75 (q, *J* = 6.0 Hz, 2H). ^{13}C{^{1}H} NMR: (125 MHz, D_2O): *δ* 167.8 (C), 157.6 (C), 151.3 (C), 150.8 (C), 147.2 (C), 146.9 (C), 137.8 (C), 134.2 (CH), 134.0 (CH), 131.4 (CH), 130.1 (CH), 128.5 (CH), 127.9 (CH), 117.3 (CH_2), 66.0 (CH_2), 64.9 (CH_2), 62.6 (CH_2), 42.2 (CH_2) ppm. HRMS (ESI) (*m/z*): calcd for $[C_{24}H_{24}N_3O_4]^+$ 418.1762, found 418.1763.

SP11: Synthesis and characterization of peptide **9**·2TFA (Fmoc-Phe-**4**-Phe-NH_2) by SPPS:

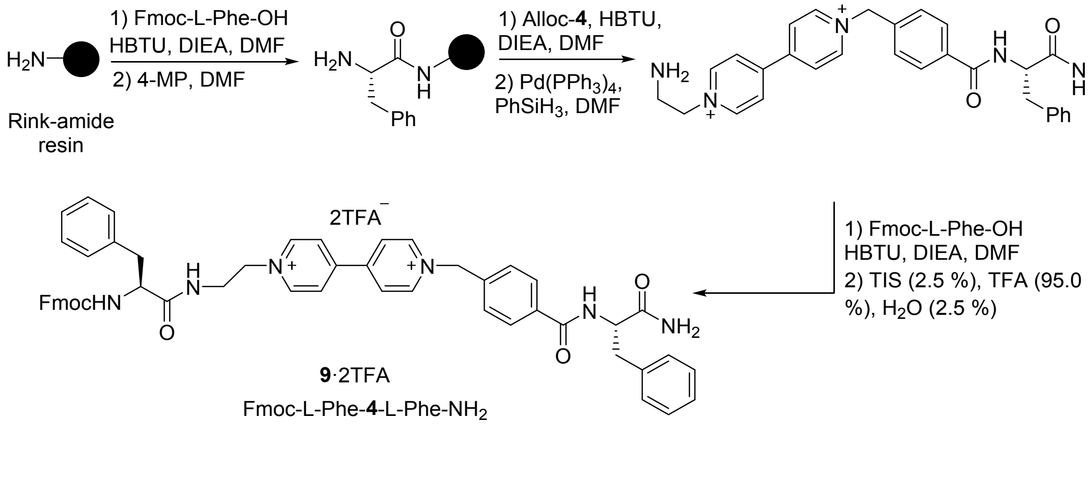

The peptide was synthesized using standard Fmoc solid phase peptide synthesis protocols using HBTU as activating agent. 0.1 mmol of a H-Rink amide ChemMatrix resin (0.47 mmol/g loading) were treated with a mixture (preactivated for 2 min) of Fmoc-Phe-OH (4 equiv.), HBTU (4.0 equiv.), and DIEA (6 equiv.) in DMF. The coupling was performed at room temperature for 30 min in a sintered funnel with continuous stirring. Then, the resin was filtered and washed with DMF (3 × 5 mL × 2 min) and DCM (3 × 5 mL × 2 min). Once the coupling was confirmed by the TNBS test, Fmoc deprotection was performed in a sintered funnel, with continuous stirring, with 20% 4-methylpiperidine (5 mL) in DMF for 15 minutes. After this time, the resin was filtered and washed with DMF (3 × 5 mL × 2 min) and DCM (3 × 5 mL × 2 min). Alloc-**4**·2Cl (2 equiv.) was activated for 2 min with HBTU (2 equiv.) and DIEA (6 equiv.) in DMF and the resulting mixture was added to the resin. The coupling was conducted at room temperature for 45 min and then, the resin was filtered and washed with DMF (3 × 5 mL × 2 min). The removal of the Alloc group was carried out by treatment of the resin with a solution of commercial $Pd(PPh_3)_4$ (0.1 equiv.) and $PhSiH_3$ (24 equiv.) in DMF (2 mL) for 10 minutes. After filtering the resin, this process was repeated twice (three times in total) and the resin was filtered and washed with DMF (3 × 5 mL × 2 min) and DCM (3 × 5 mL × 2 min). The final Fmoc-Phe-OH was coupled following the same protocol than for the first one. For the final cleavage-deprotection step, 3 mL of the cleavage cocktail (2.5% H_2O, 2.5% triisopropysilane (TIS) and 95% TFA) were added to the resin-bound peptide (0.1 mmol). After shaking the resulting mixture for 3 h, the resin was filtered and the TFA filtrate was then added to ice-cold Et_2O (40 mL). The resulting precipitate was then centrifuged, washed again with ice-cold Et_2O (10 mL) and centrifuged. The obtained solid was dried under argon, redissolved in MeCN/H_2O (1:1, 1.5 mL), and purified by semipreparative RP-HPLC. The collected fractions were lyophilized and stored at –20 °C. On a 0.1 mmol scale, **9**·2TFA was obtained in a 6% yield (6 mg).

9·2TFA: ^1H NMR (500 MHz, CD_3CN): δ 8.90 (d, J = 7.1 Hz, 2H), 8.77 (d, J = 6.5 Hz, 2H), 8.52 (s, 1H), 8.19 (m, 4H), 7.78 (d, J = 8.4 Hz, 2H), 7.71 (d, J = 7.4 Hz, 2H), 7.69 (s, 1H), 7.51 (d, J = 7.0 Hz, 1H), 7.45 (d, J = 7.6 Hz, 1H), 7.42 (d, J = 8.2 Hz), 7.33 (m, 2H), 7.22 (m, 10H), 7.13 (m, 2H), 6.82 (s, 1H), 6.78 (s, 1H), 5.78 (s, 1H), 5.72 (d, J = 6.2 Hz, 2H), 4.72 (t, J = 5.4 Hz, 2H), 4.67 (m, 1H), 4.10 (m, 2H), 4.02 (m, 2H), 3.84 (m, 1H), 3.67 (m, 1H), 3.19 (dd, J = 14.0, 4.9 Hz, 1H), 2.99 (m, 1H), 2.97 (m, 1H), 2.74 (dd, J = 13.9, 5.6 Hz, 1H). $^{13}C\{^1H\}$ NMR: (125 MHz, CD_3CN): δ 174.1 (C), 174.0 (C), 166.8 (C), 157.1 (C), 151.2 (C), 150.3 (C), 147.2 (CH), 146.5 (CH), 144.9 (C), 144.8 (C), 141.9 (C), 138.9 (C), 138.8 (C), 136.6 (C), 130.2 (CH), 130.2 (CH), 129.3 (CH), 129.2 (CH), 128.8 (CH), 128.7 (CH), 128.2 (CH), 128.2 (CH), 127.7 (CH), 127.6 (CH), 127.5 (CH), 126.4 (CH), 126.3 (CH), 121.0 (CH), 120.9 (CH), 70.9 (CH_2), 67.2 (CH_2), 64.9 (CH_2), 62.1 (CH_2), 58.2 (CH), 56.1 (CH), 47.8 (CH), 40.3 (CH_2), 38.3 (CH_2) ppm. HRMS (ESI) (m/z): calcd for $[C_{53}H_{50}N_6O_5]^{+2}$ 425.1916, found 425.1915.

SP12: Self-assembly of **2H**·3Cl with cucurbit[7]uril:

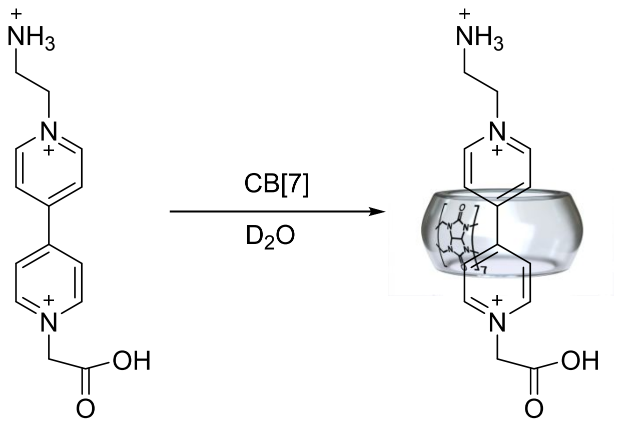

2H·3Cl **2H⊂** CB7

Firstly, guest **2H**·3Cl (1.83 mg, 0.005 mmol) was dissolved in 5 mL of D_2O with 50 mM phosphate buffer solution to reach pD=7. On the other hand, host CB[7] (10.5 mg) was dissolved in 1.5 mL of the previous solution in order to keep constant the concentration of the guest. Then, an NMR titration was performed using 0.5 mL of first solution and different amounts of second solution by recording the corresponding ^{1}H-NMR.

2H⊂CB[7]: ^{1}H NMR (500 MHz, D_2O): δ 9.10 (d, J = 6.3 Hz, 2H), 8.30 (s, 2H), 7.89 (s, 2H), 7.41 (s, 2H), 5.79 (d, J = 15.4 Hz, 14H), 5.58 (s, 2H), 5.46 (s, 14H), 4.69 (s, 2H), 4.26 (d, J = 15.6 Hz, 14H), 3.35 (s, 2H). HRMS (ESI) (*m/z*): calcd for $[C_{56}H_{60}N_{31}O_{16}]^{+2}$ 711.7428, found 711.7434.

SP13: Self-assembly of **2H**·3Cl with cucurbit[8]uril:

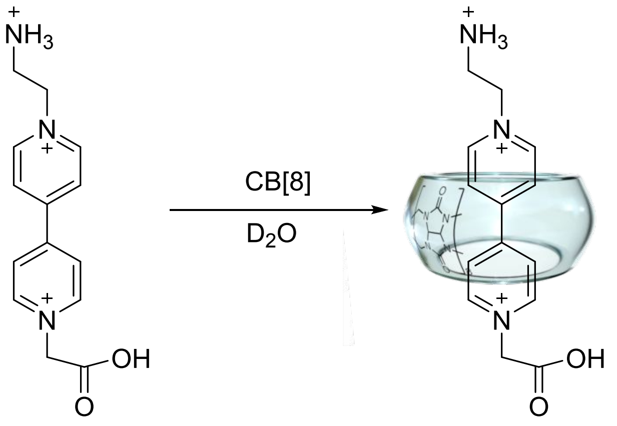

2H·3Cl **2H⊂** CB8

To a 1mM solution of guest **2H**·3Cl in D_2O with 50 mM phosphate buffer solution at pD=7 different amounts of CB[8] (as solid) were added until the solution reached saturation.

2H⊂CB[8]: ^{1}H NMR (500 MHz, D_2O): δ 8.90 (d, J = 6.3 Hz, 2H), 8.70 (s, 2H), 7.84 (s, 2H), 7.72 (s, 2H), 5.74 (d, J = 15.3 Hz, 16H), 5.56 (overlap, 2H), 5.51 (s, 16H), 5.00 (overlap with solvent, 2H), 4.20 (d, J = 15.3 Hz, 16H), 3.71 (s, 2H). HRMS (ESI) (*m/z*): $[C_{62}H_{66}N_{35}O_{18}]^{+2}$ 794.7674, found 794.7679..

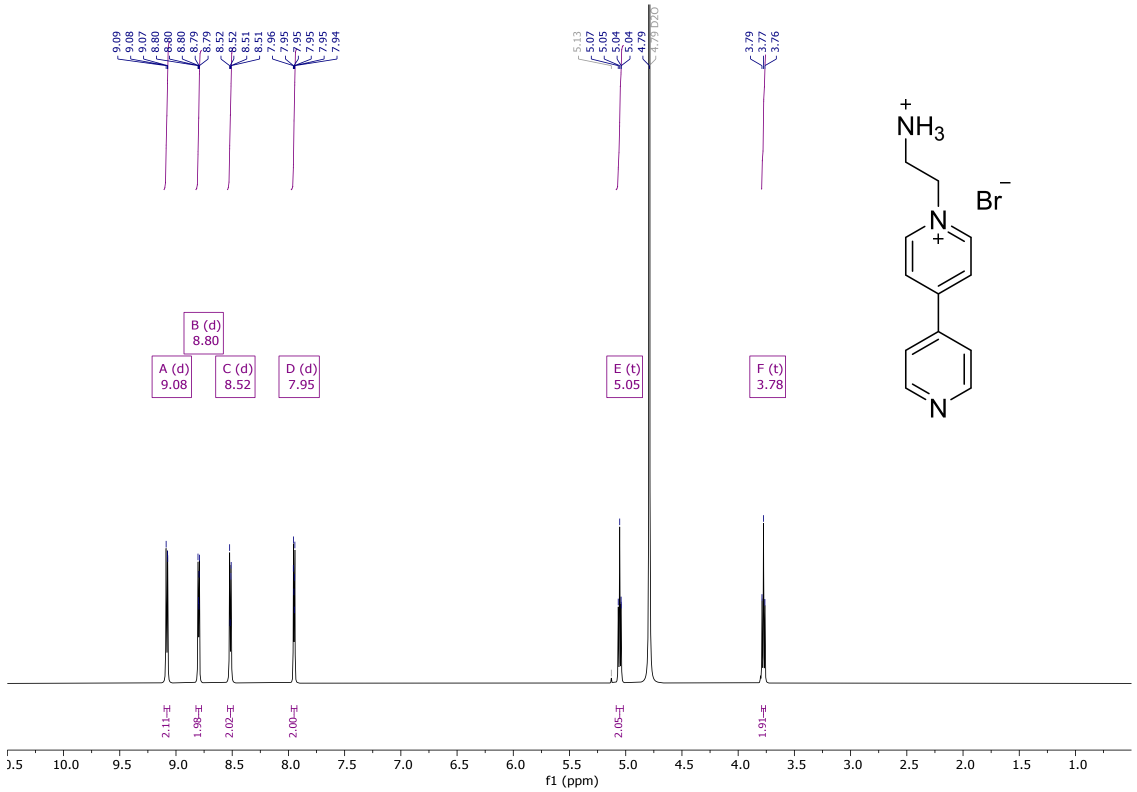

Figure S1: ^{1}H NMR (500 MHz, D_2O) spectrum of 1H·2Br.

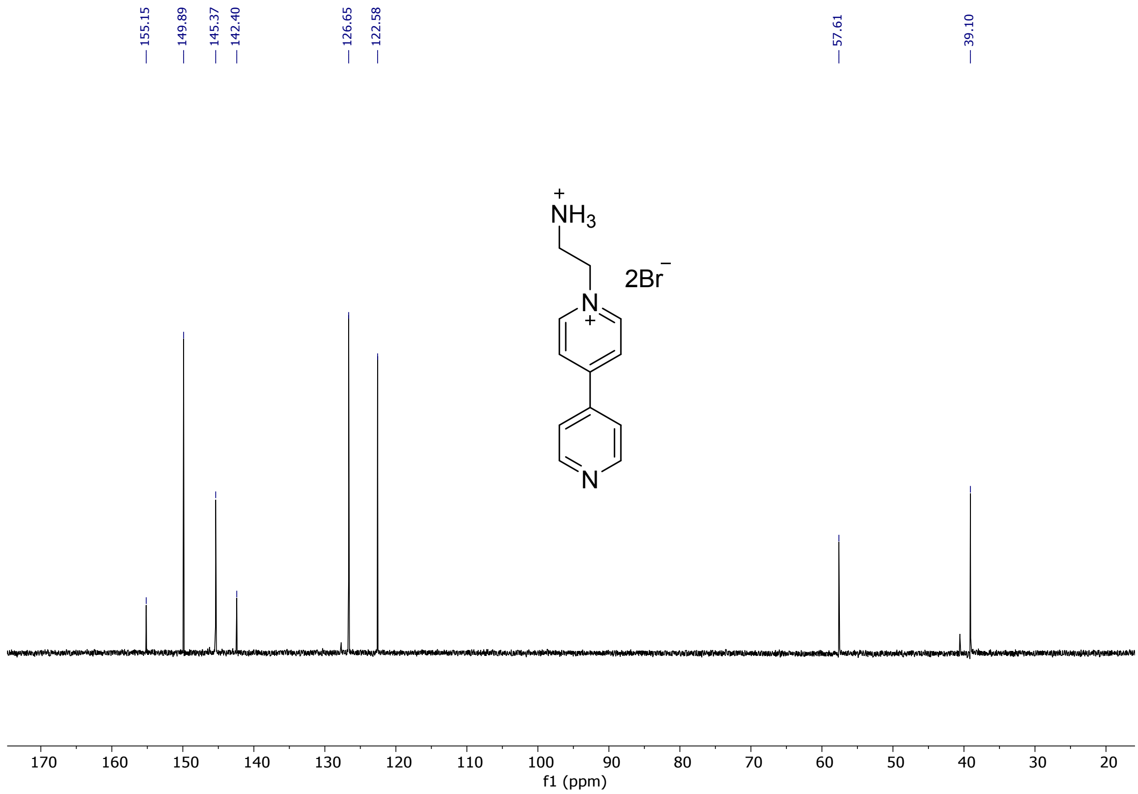

Figure S2: $^{13}C\{^1H\}$ NMR (125 MHz, D_2O) spectrum of 1H·2Br.

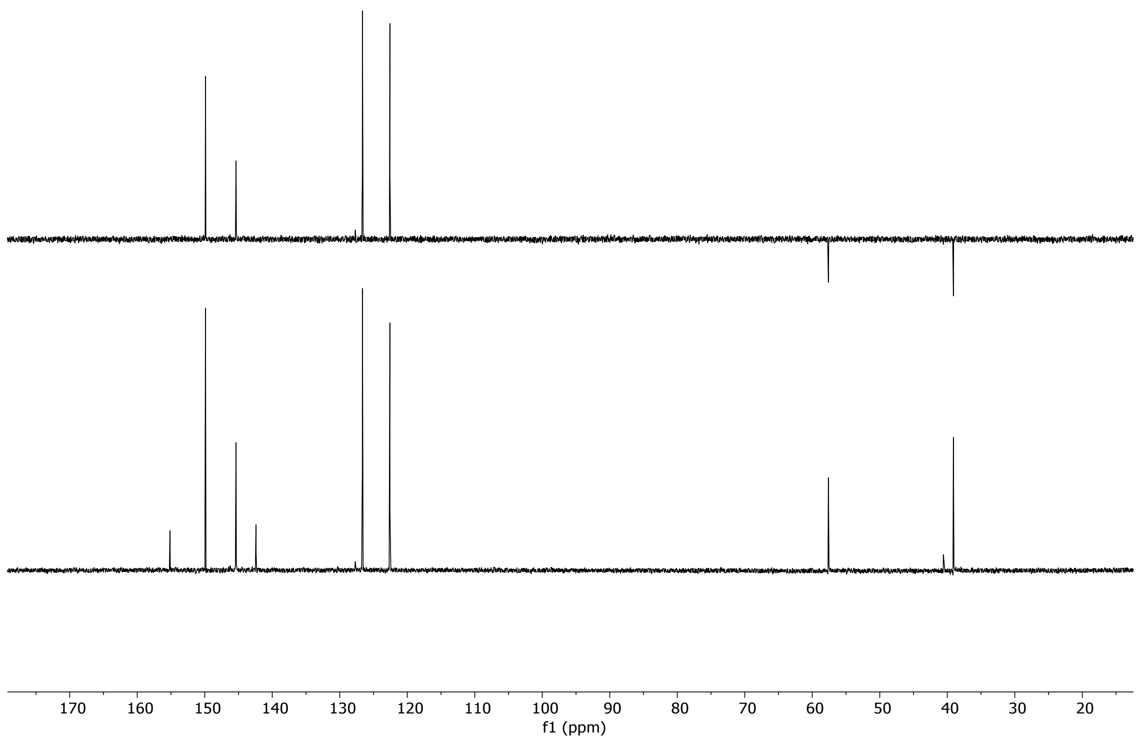

Figure S3: $^{13}C\{^1H\}$ NMR and DEPT (125 MHz, D_2O) spectrum of 1H·2Br.

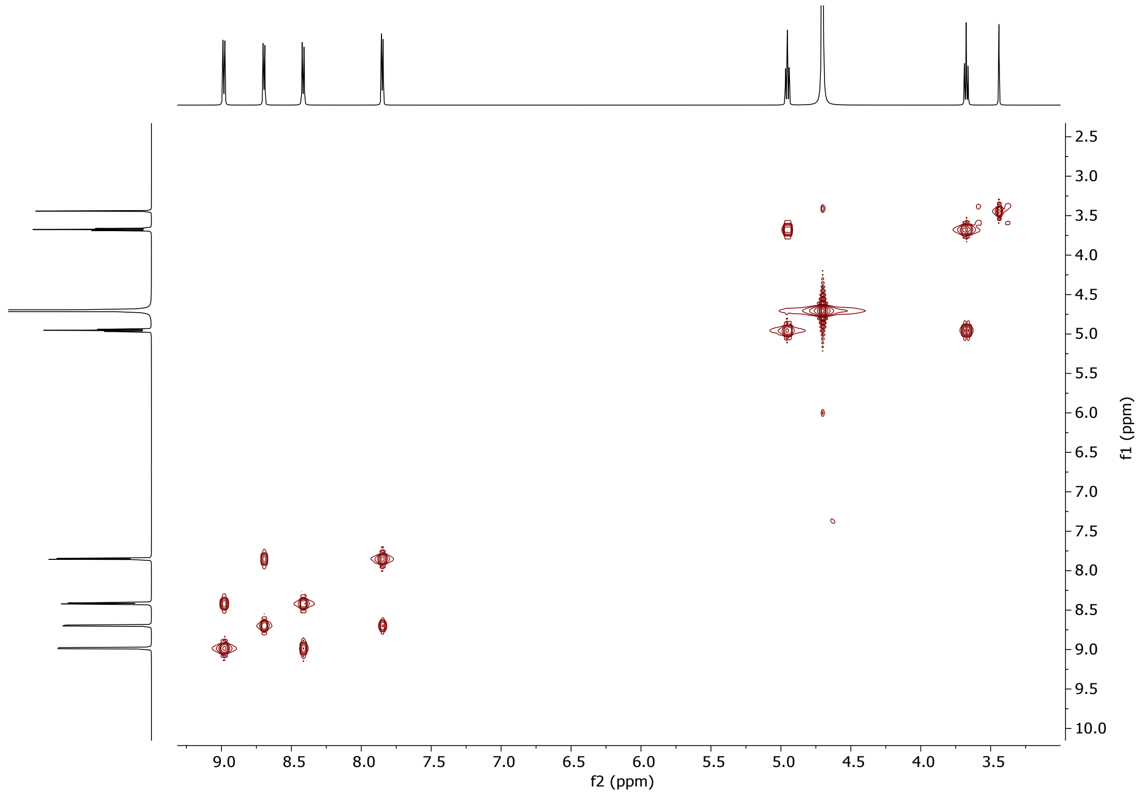

Figure S4: COSY (500 MHz, D_2O) spectrum of 1H·2Br.

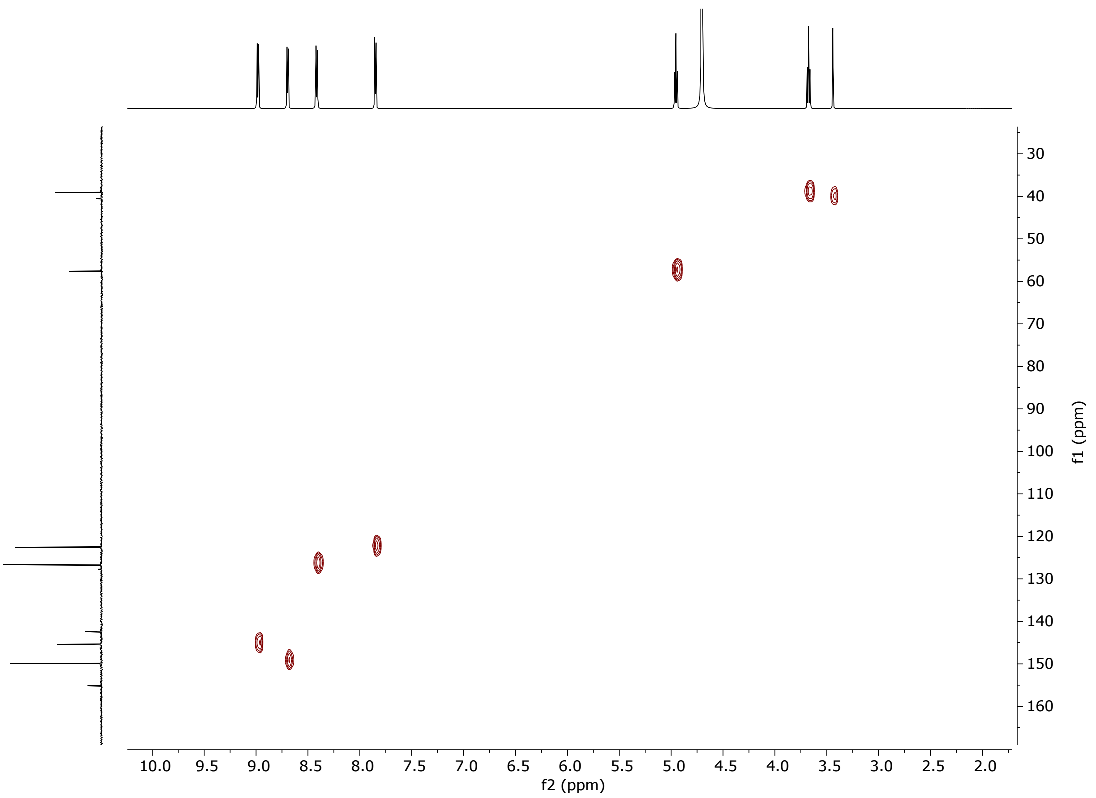

Figure S5: HSQC (500 MHz, D_2O) spectrum of 1H·2Br.

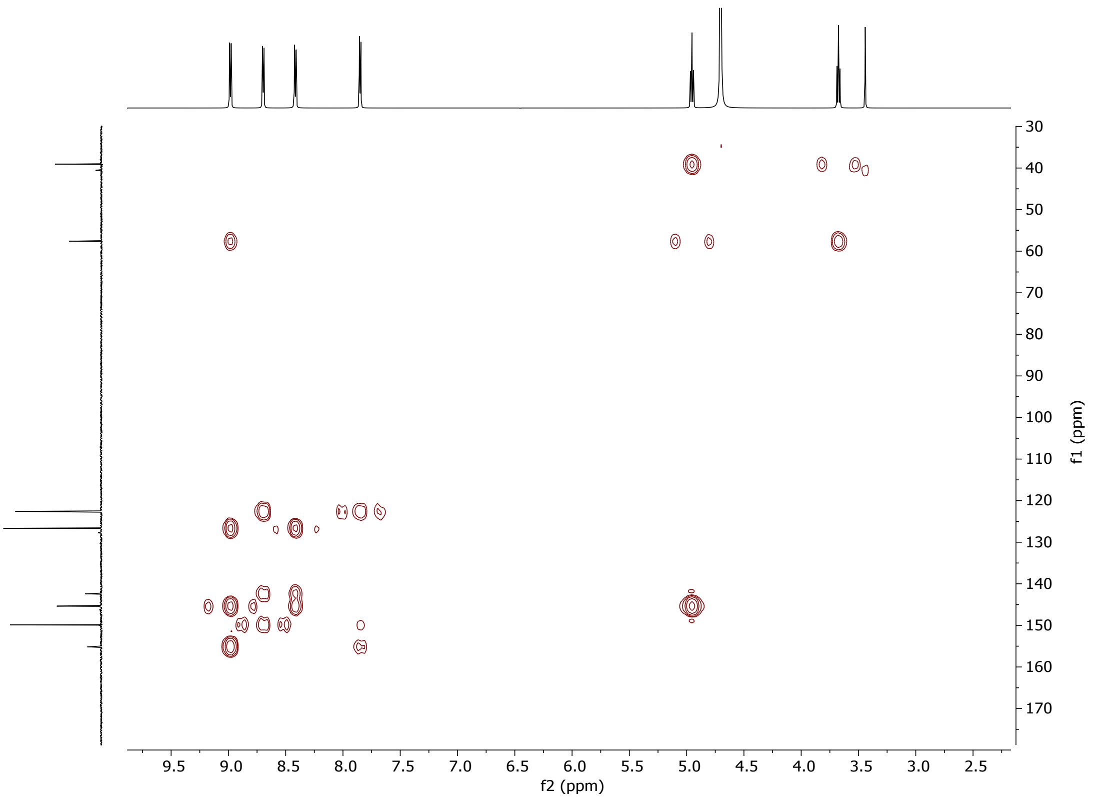

Figure S6: HMBC (500 MHz, D_2O) spectrum of 1H·2Br.

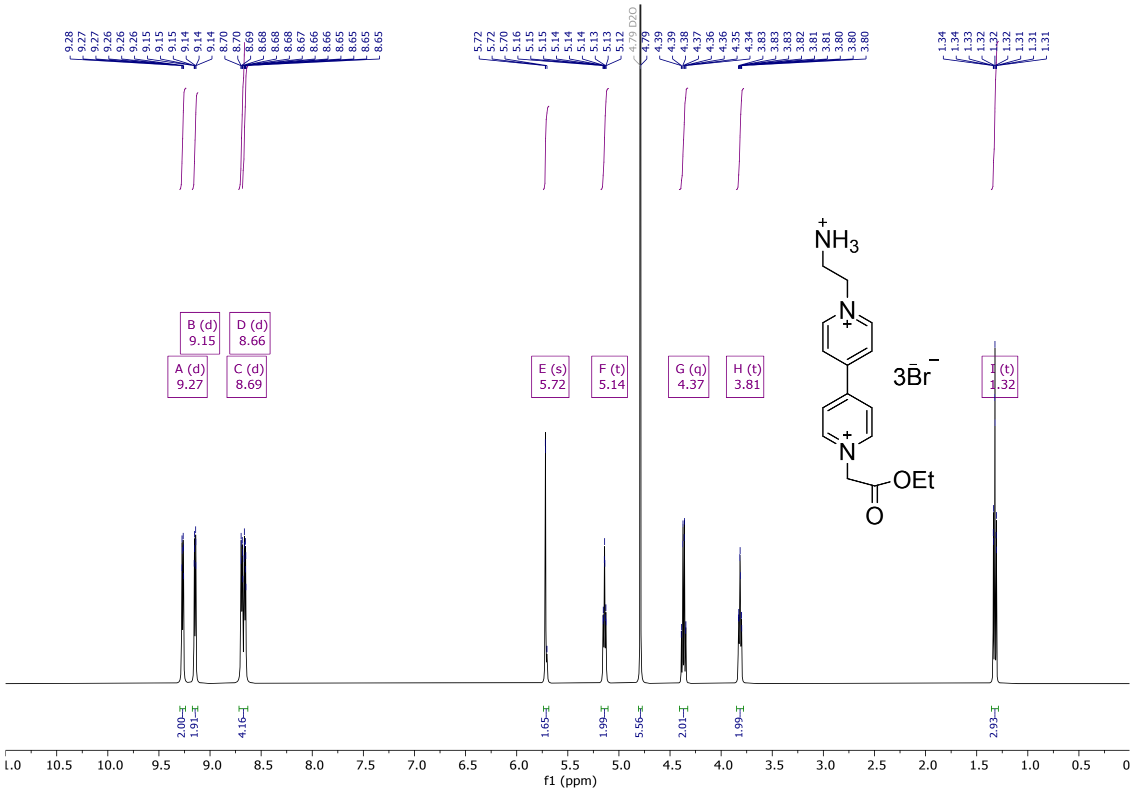

Figure S7: ^{1}H NMR (500 MHz, D_2O) spectrum of E·3Br.

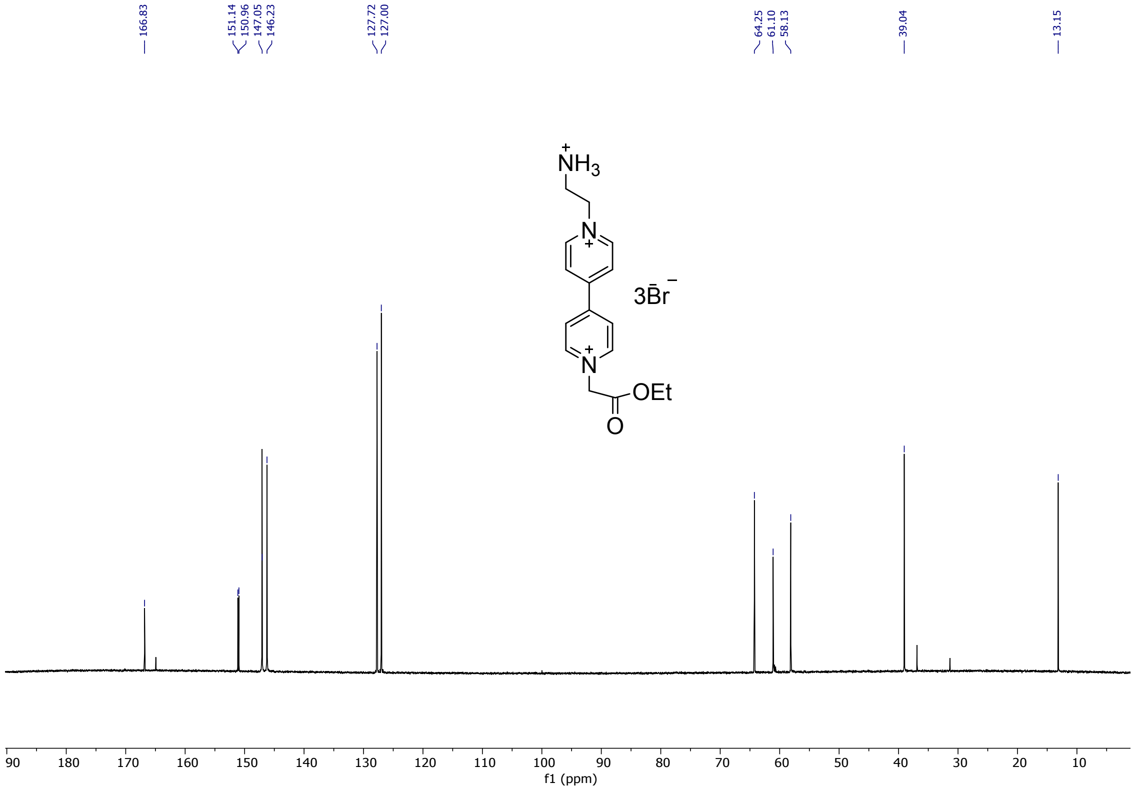

Figure S8: $^{13}C\{^{1}H\}$ NMR (125 MHz, D_2O) spectrum of E·3Br.

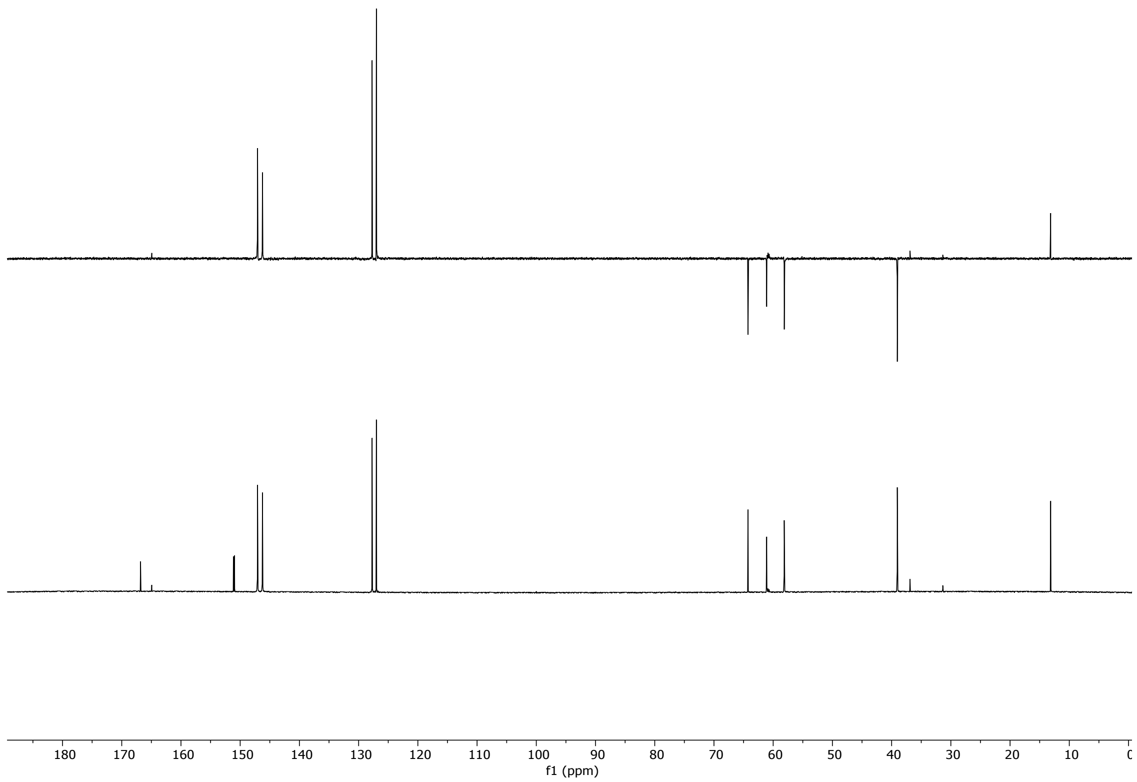

Figure S9: $^{13}C\{^1H\}$ NMR and DEPT (125 MHz, D_2O) spectrum of E·3Br.

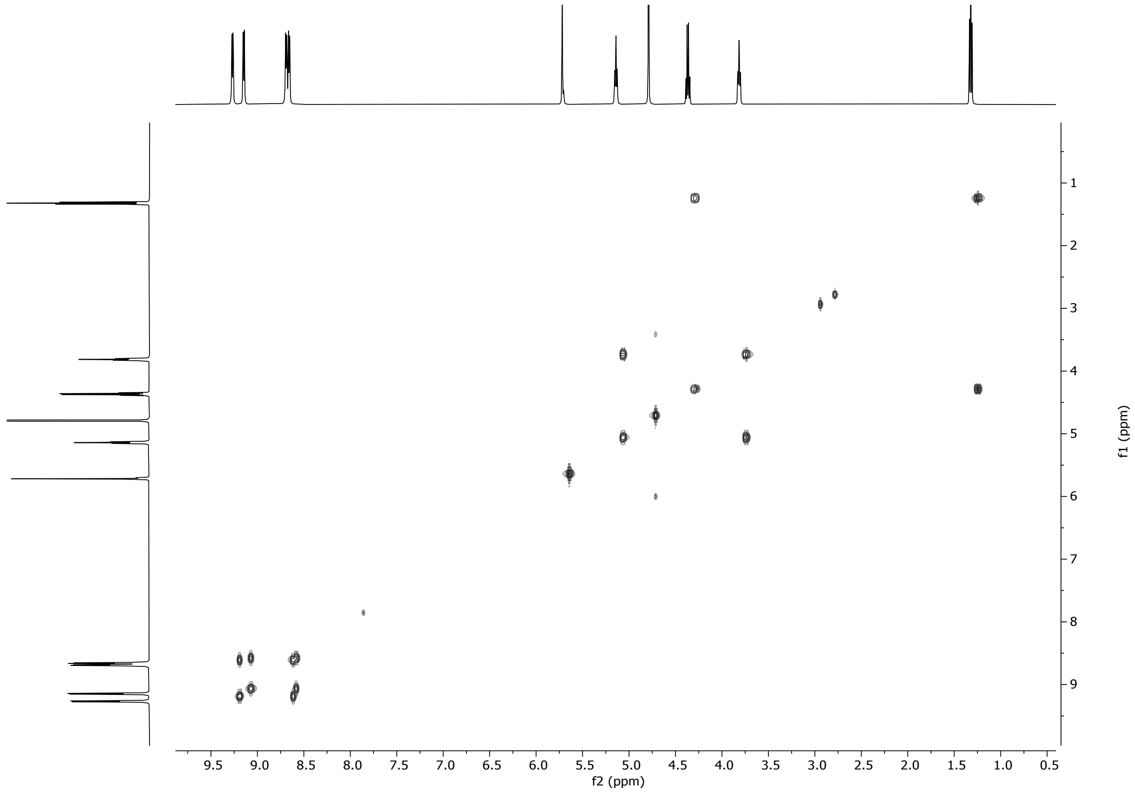

Figure S10: COSY (500 MHz, D_2O) spectrum of E·3Br.

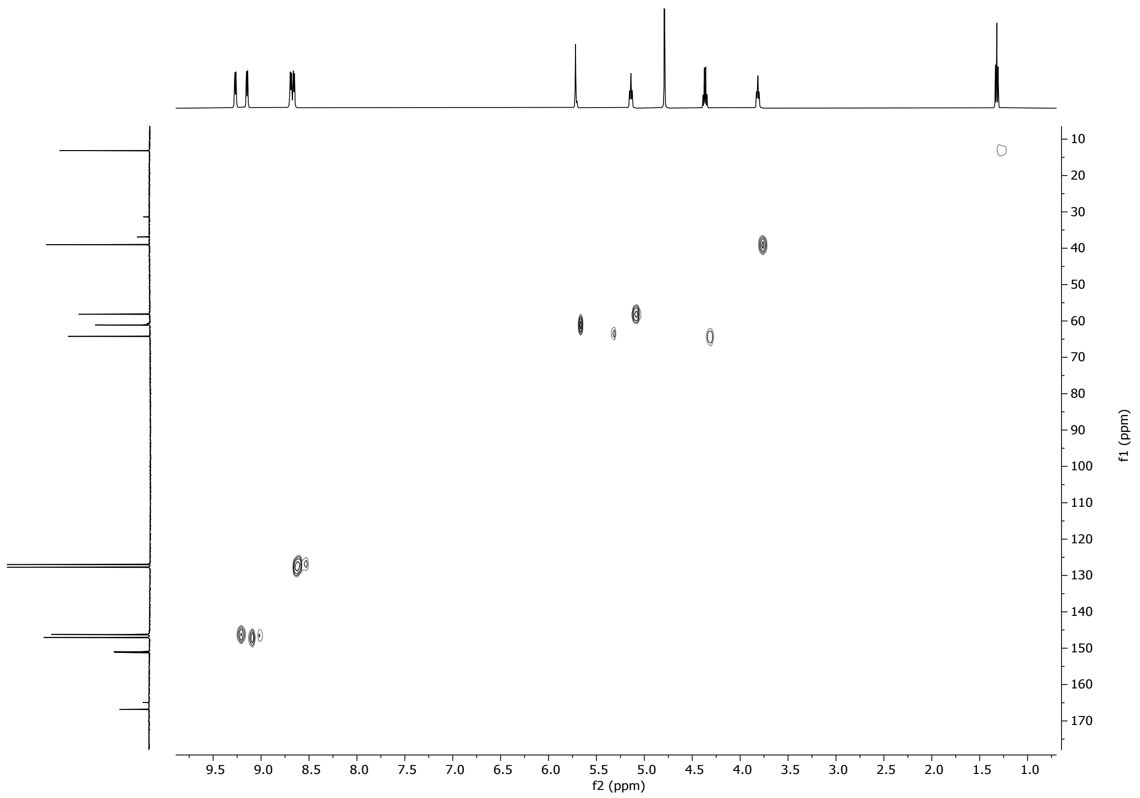

Figure S11: HSQC (500 MHz, D_2O) spectrum of E·3Br.

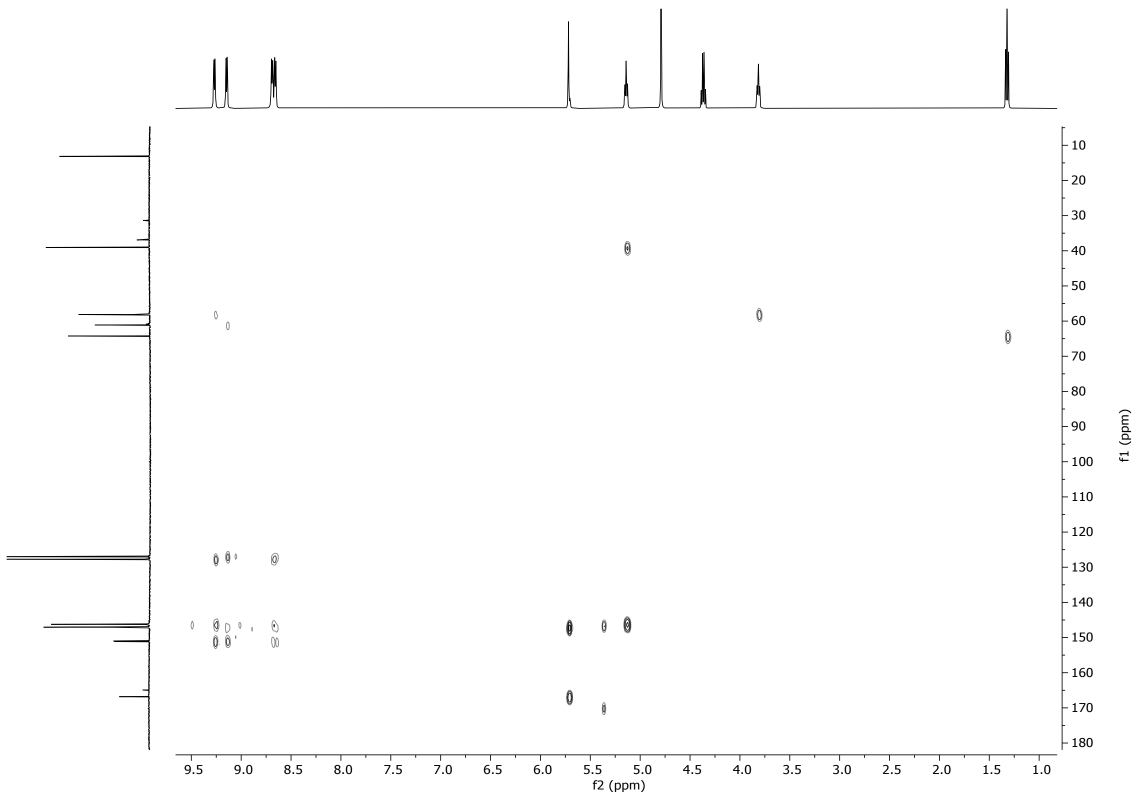

Figure S12: HMBC (500 MHz, D_2O) spectrum of E·3Br.

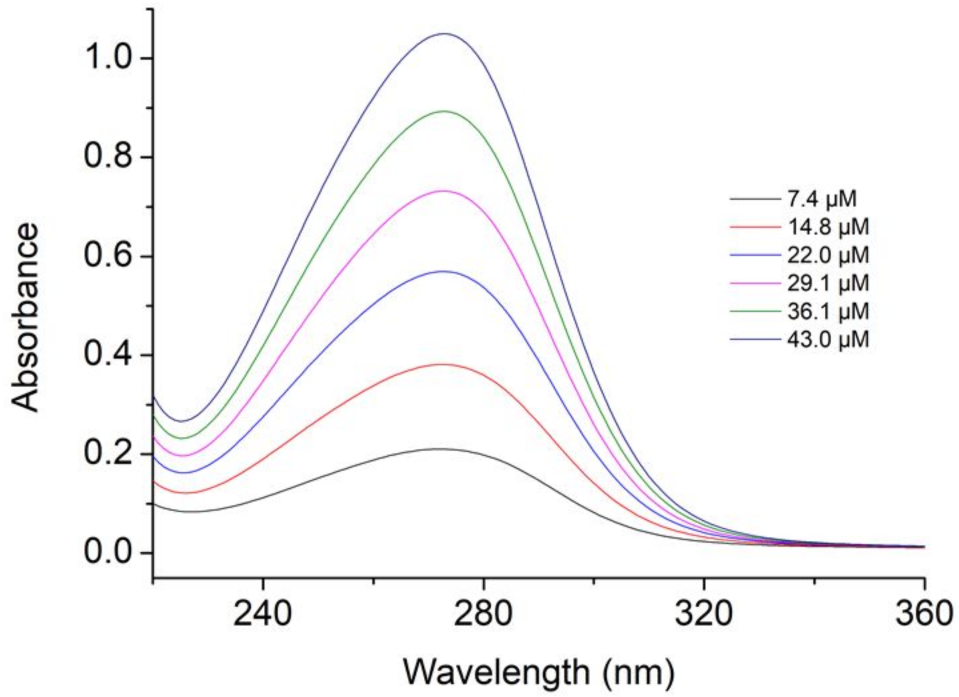

Figure S13: UV-Vis spectra of E·3Br in 50mM phosphate buffer solution, pH=7 from 7.4 μM to 43.0 μM.

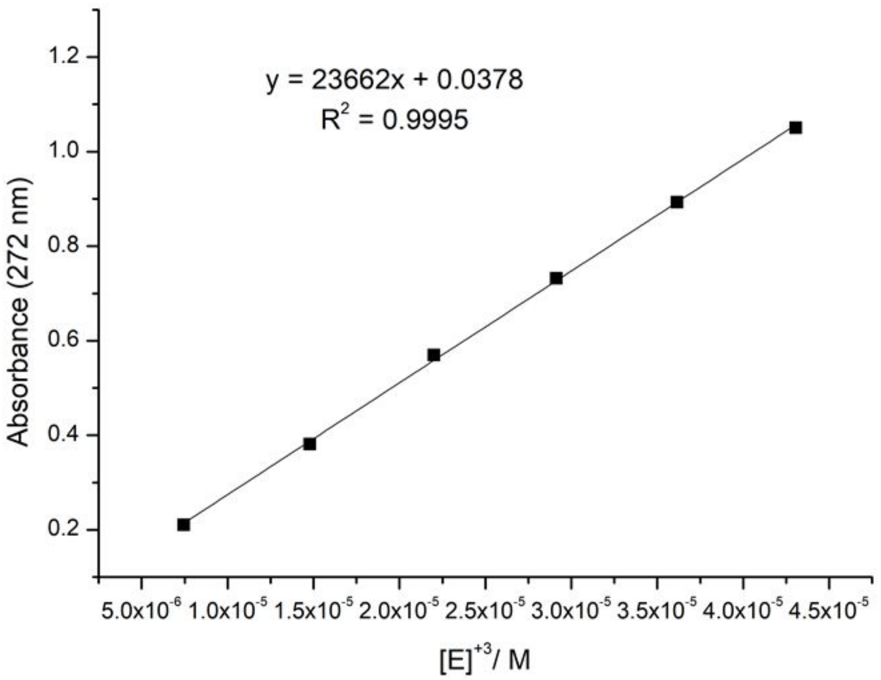

Figure S14: Linear relationship between the absorbance at 272 nm and the concentration of E^{+3} where ε=23662 $Lmol^{-1}cm^{-1}$.

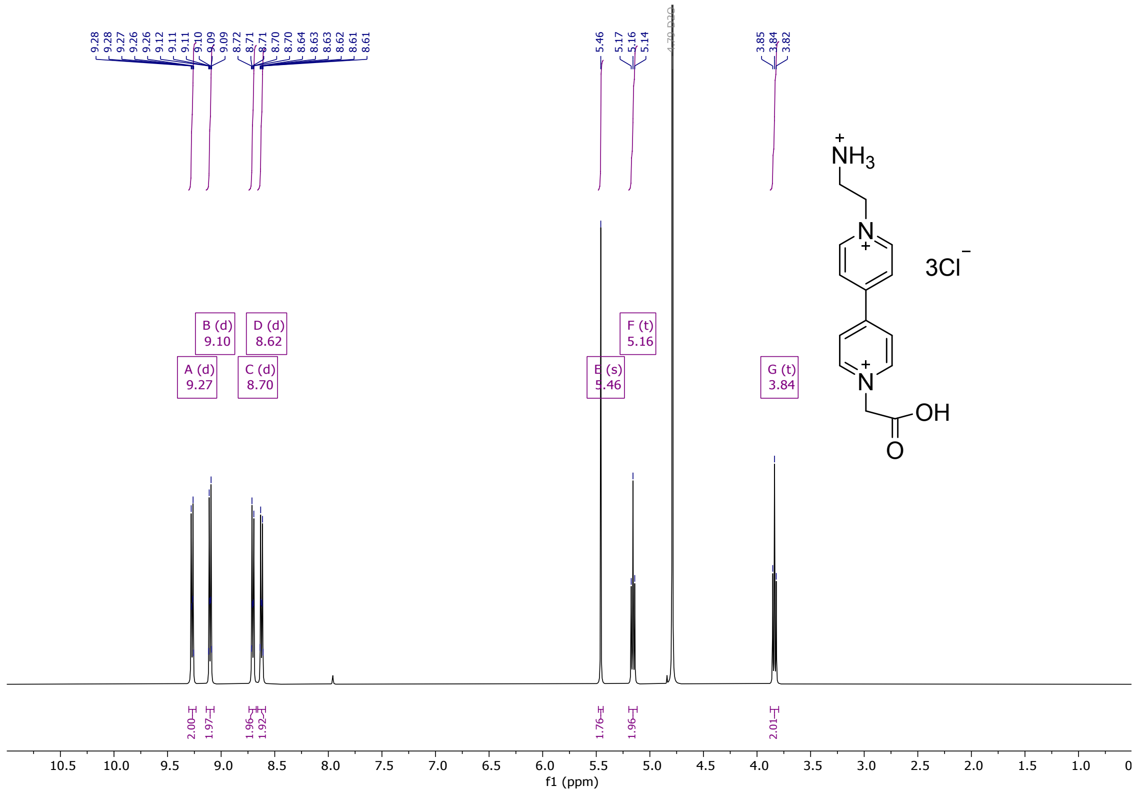

Figure S15: ^{1}H NMR (500 MHz, D_2O) spectrum of 2H·3Cl.

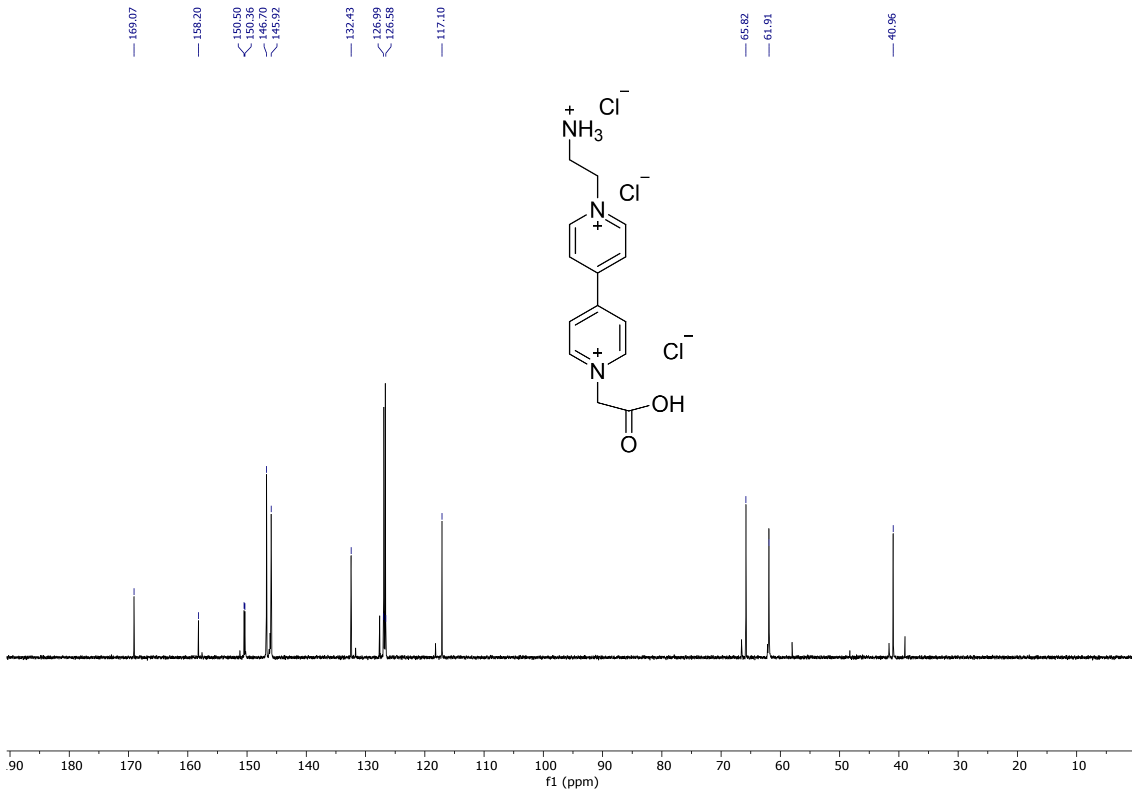

Figure S16: $^{13}C\{^{1}H\}$ NMR (125 MHz, D_2O) spectrum of 2H·3Cl.

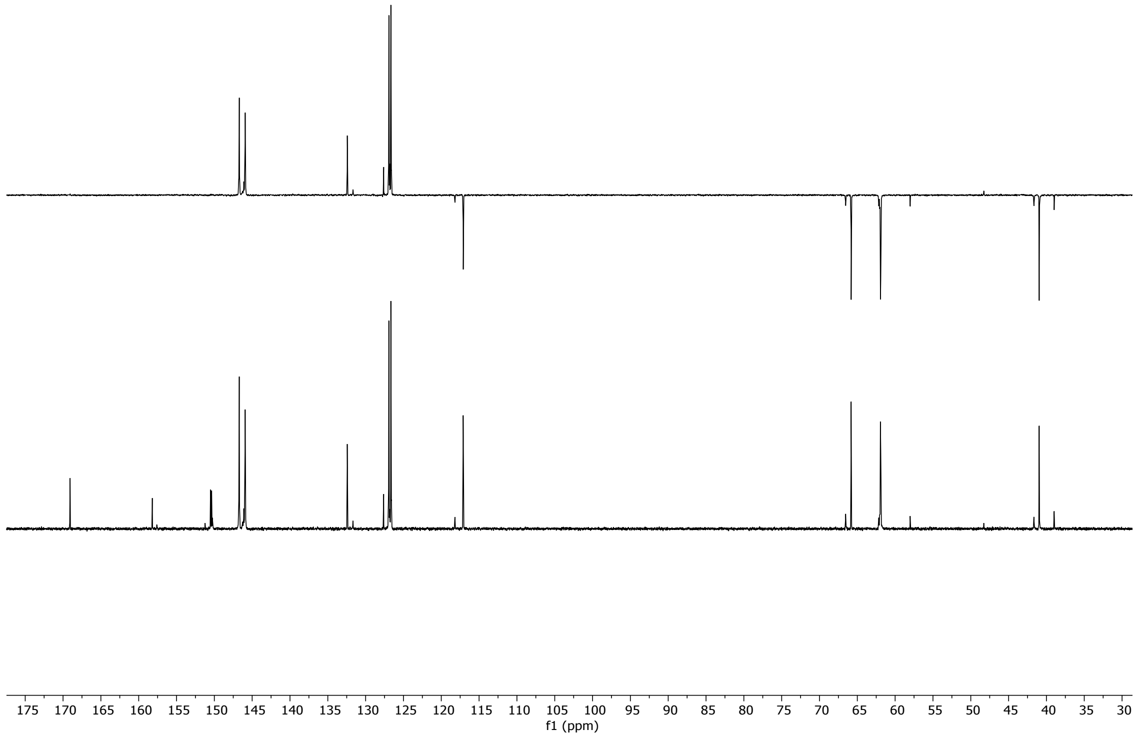

Figure S17: $^{13}C\{^1H\}$ NMR and DEPT (125 MHz, D_2O) spectrum of 2H·3Cl.

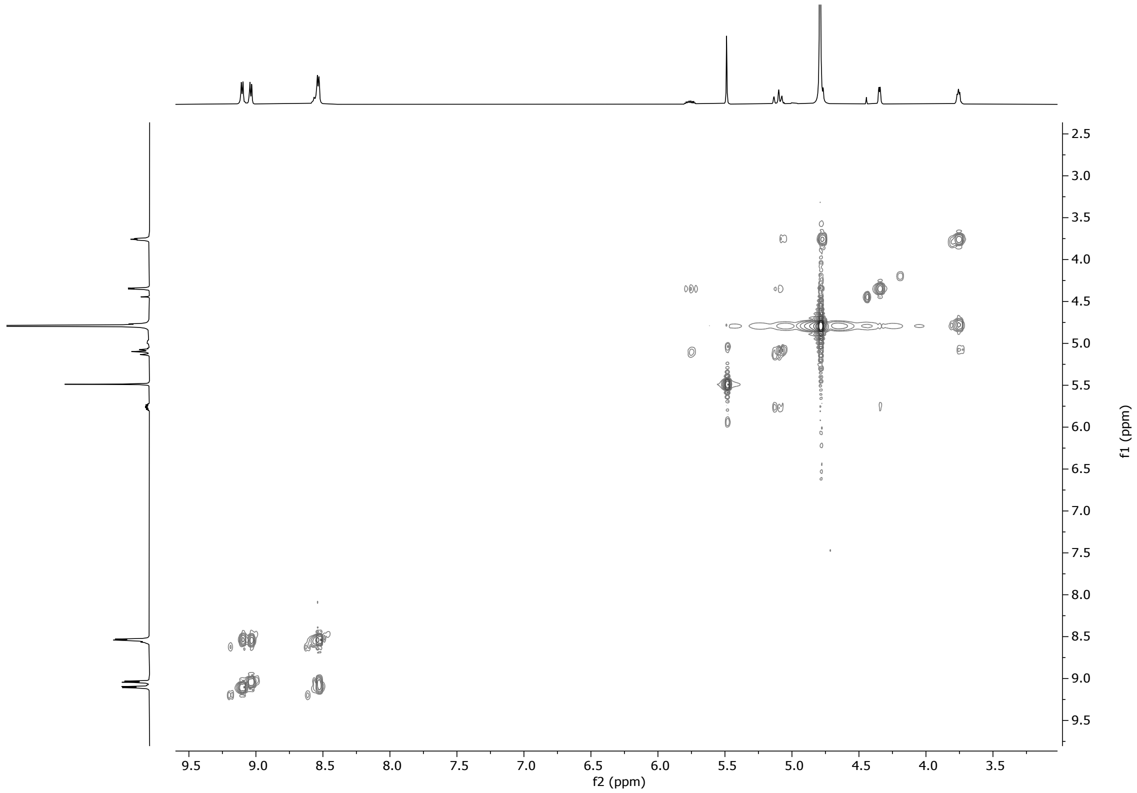

Figure S18: COSY (500 MHz, D_2O) spectrum of 2H·3Cl.

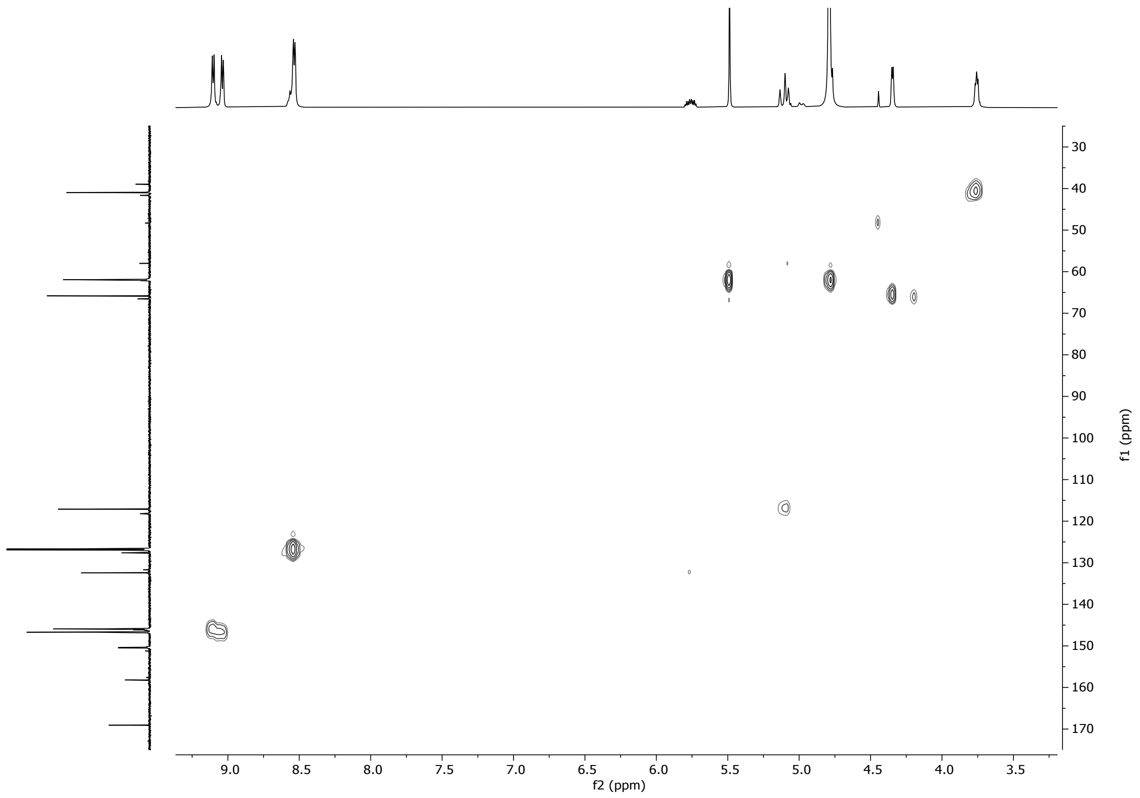

Figure S19: HSQC (500 MHz, D_2O) spectrum of 2H·3Cl.

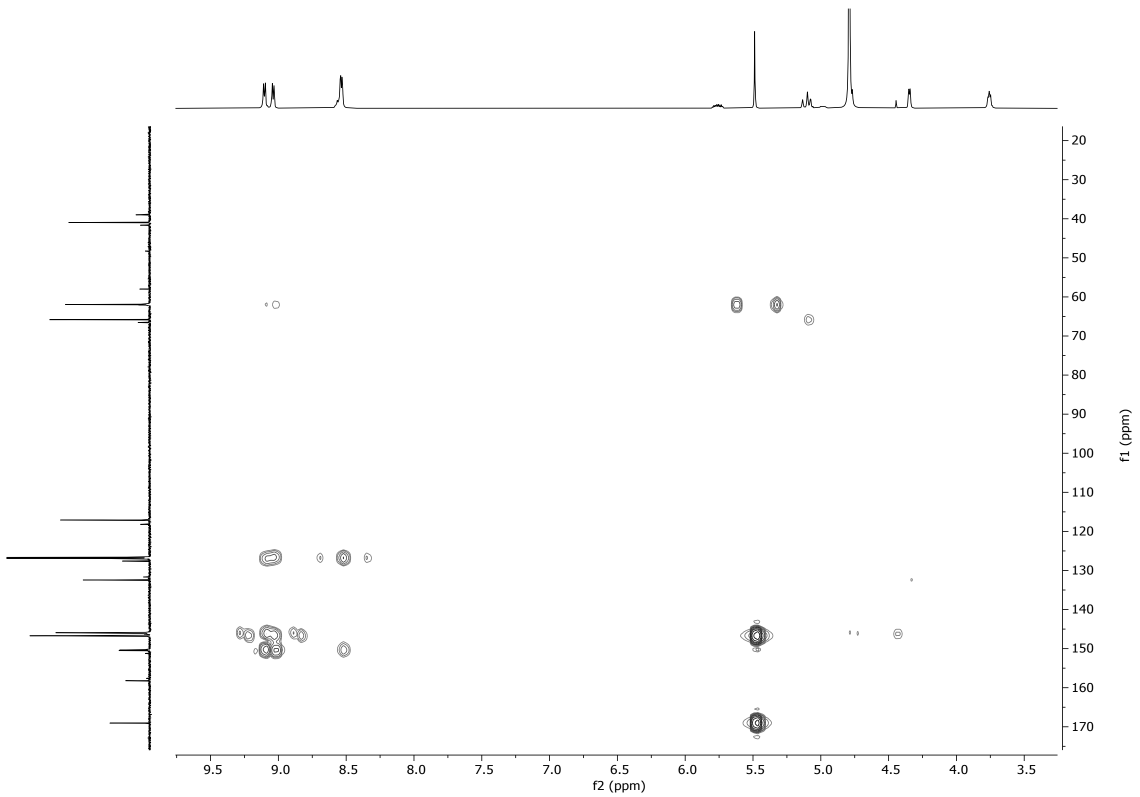

Figure S20: HMBC (500 MHz, D_2O) spectrum of 2H·3Cl.

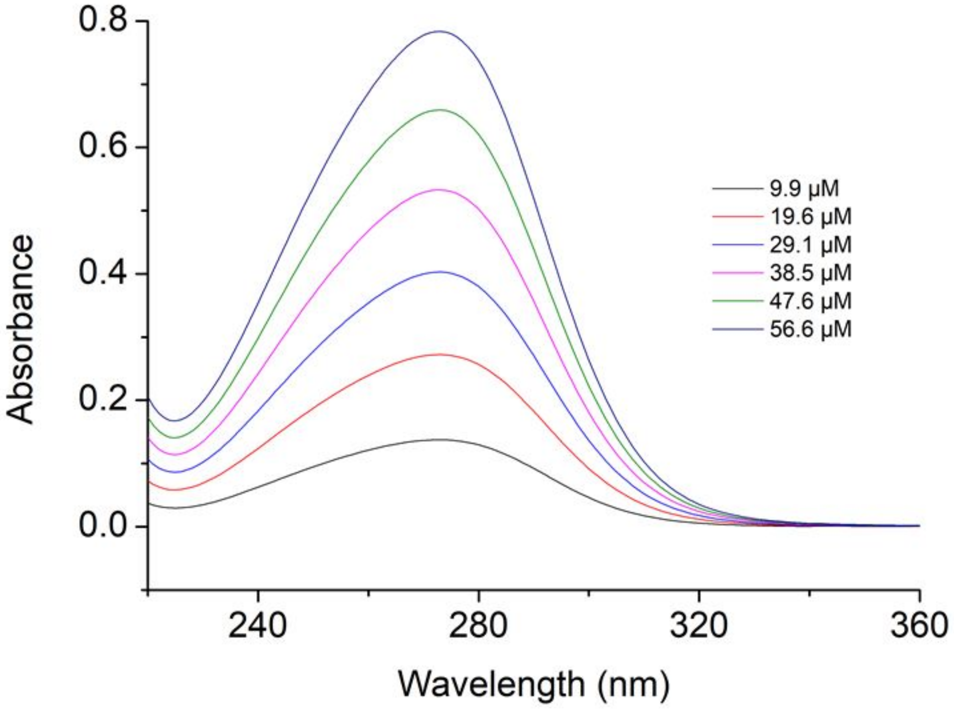

Figure S21: UV-Vis spectra of 2H·3Br in 50 mM phosphate buffer solution, pH 7 from 9.9 µM to 56.6 µM.

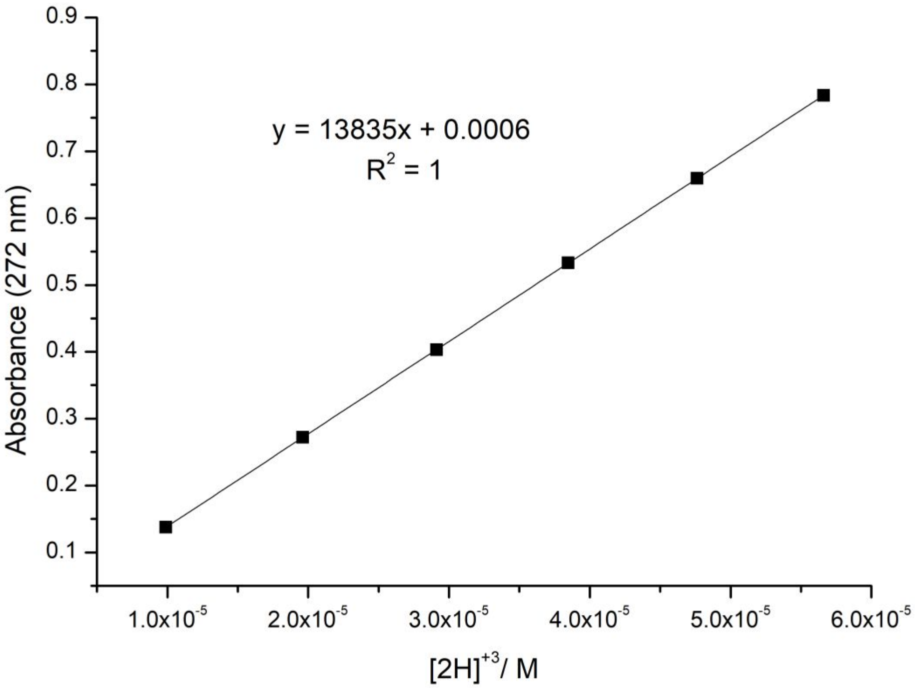

Figure S22: Linear relationship between the absorbance at 272 nm and the concentration of $2H^{+3}$ where ε=13835 $Lmol^{-1}cm^{-1}$.

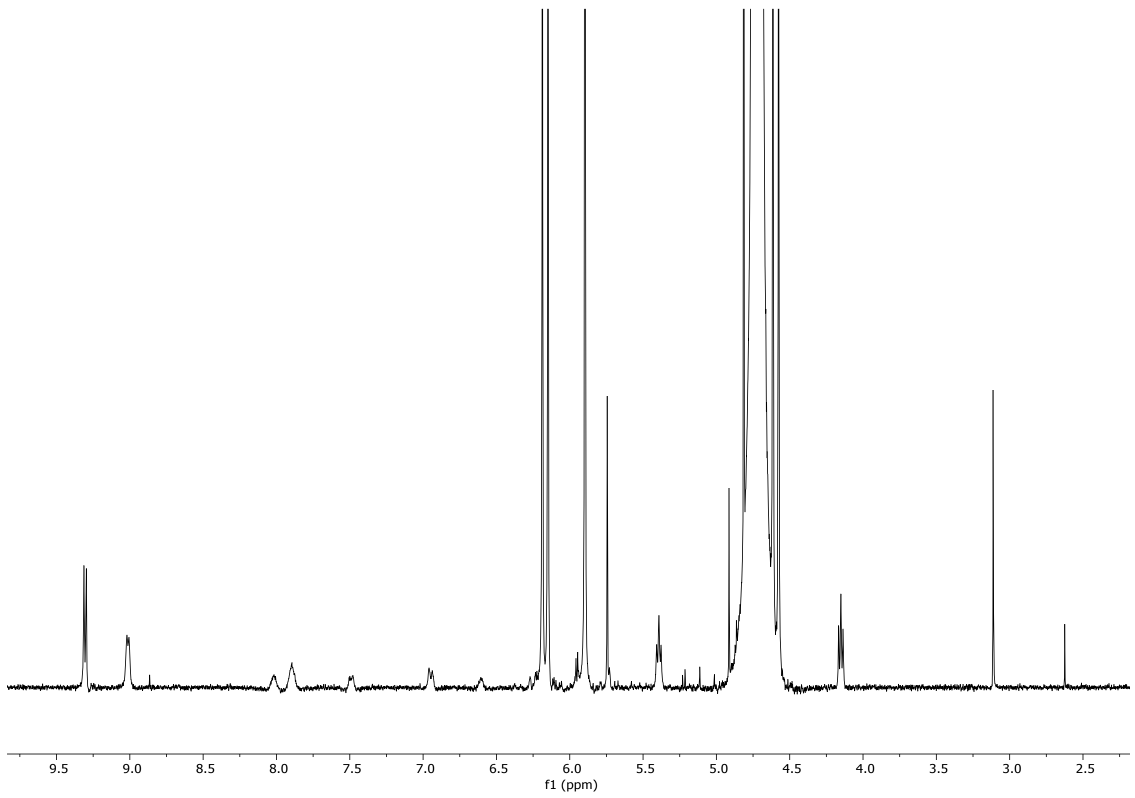

Figure S23: ^1H NMR (400 MHz, D_2O, pD=7) spectrum of a 1mM 2H·3Cl, CB8, and 2,7-DHN mixture.

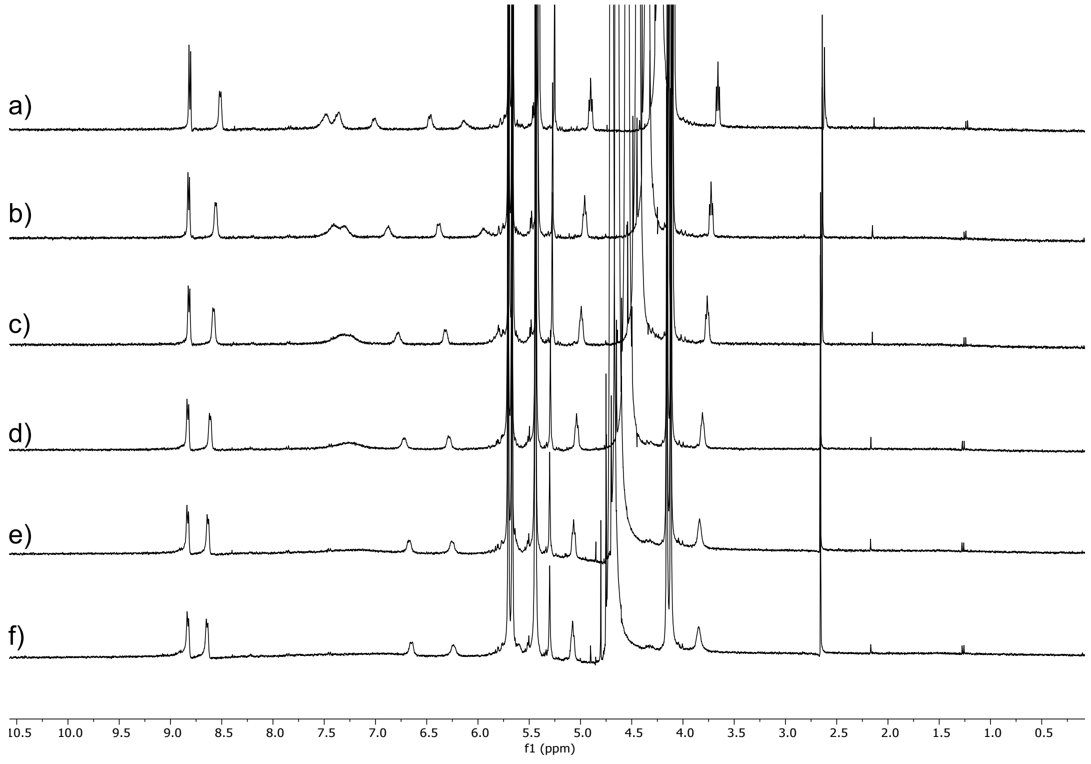

Figure S24: T. Var. (400 MHz, D_2O, pD=7) spectrum of a 1mM 2H·3Cl, CB8, and 2,7-DHN mixture at: a) 348.15 K b) 345.15 K c) 338.15 K d) 328.15 K e) 318.15 K f) 308.15 K g) 298.15 K.

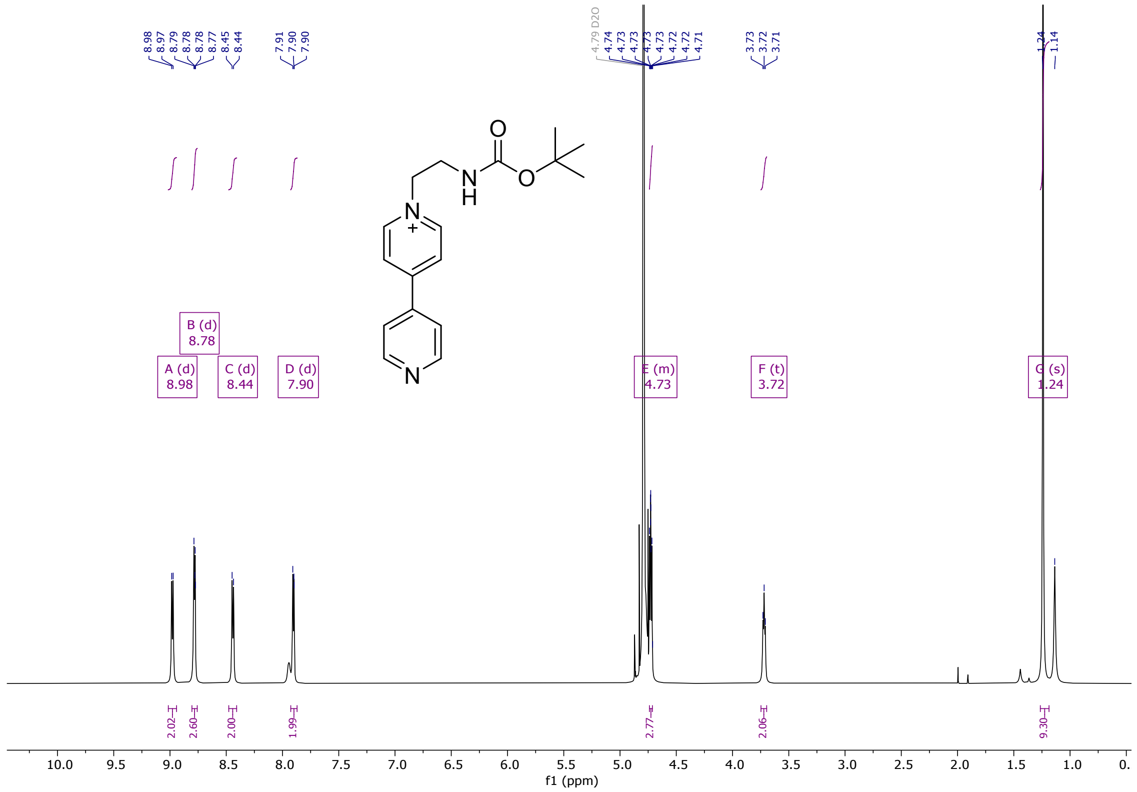

Figure S25: ^{1}H NMR (500 MHz, D_2O) spectrum of 3·Cl.

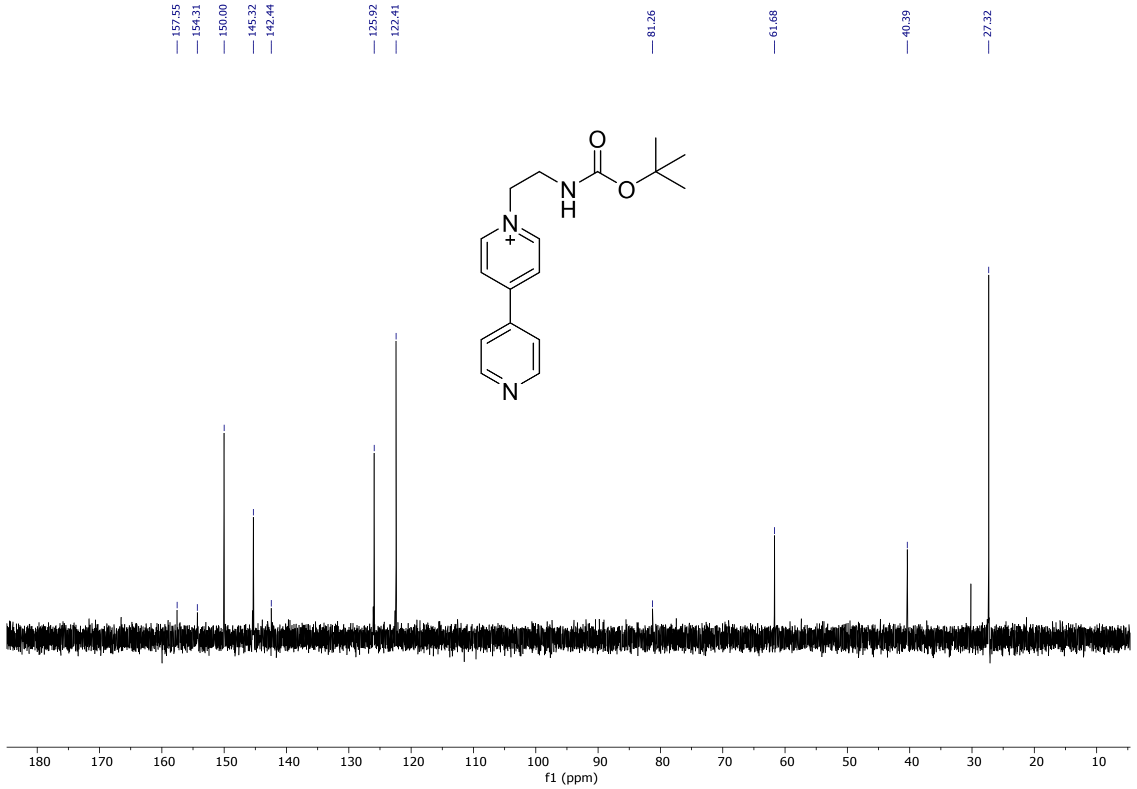

Figure S26: $^{13}C\{^{1}H\}$ NMR (125 MHz, D_2O) spectrum of 3·Cl.

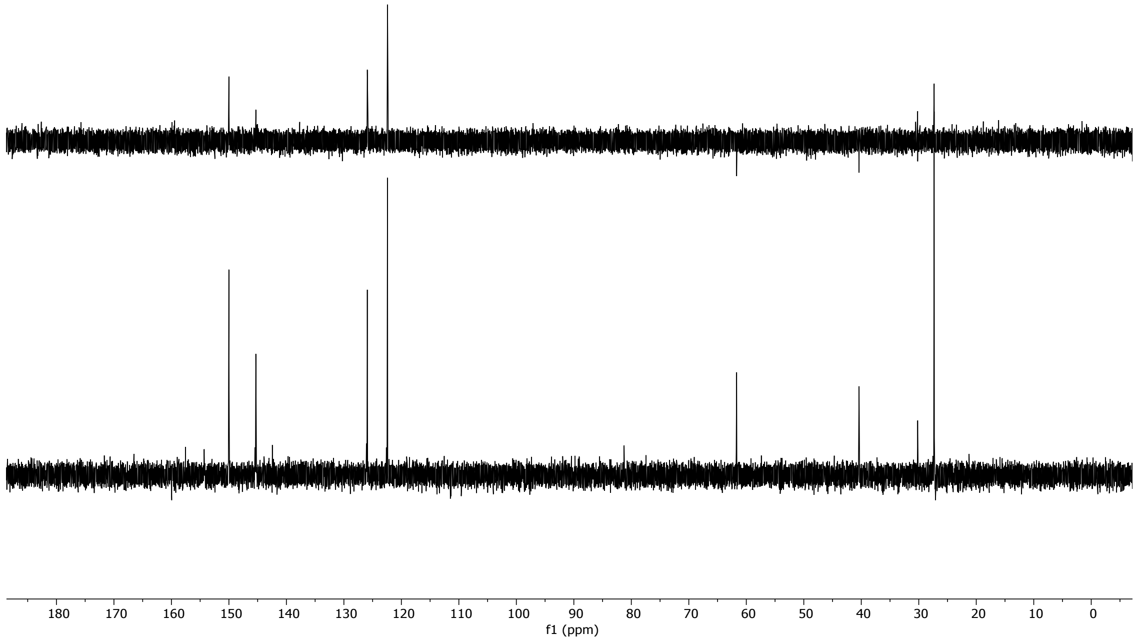

Figure S27: $^{13}C\{^1H\}$ **NMR and DEPT (125 MHz,** D_2O**) spectrum of 3·Cl.**

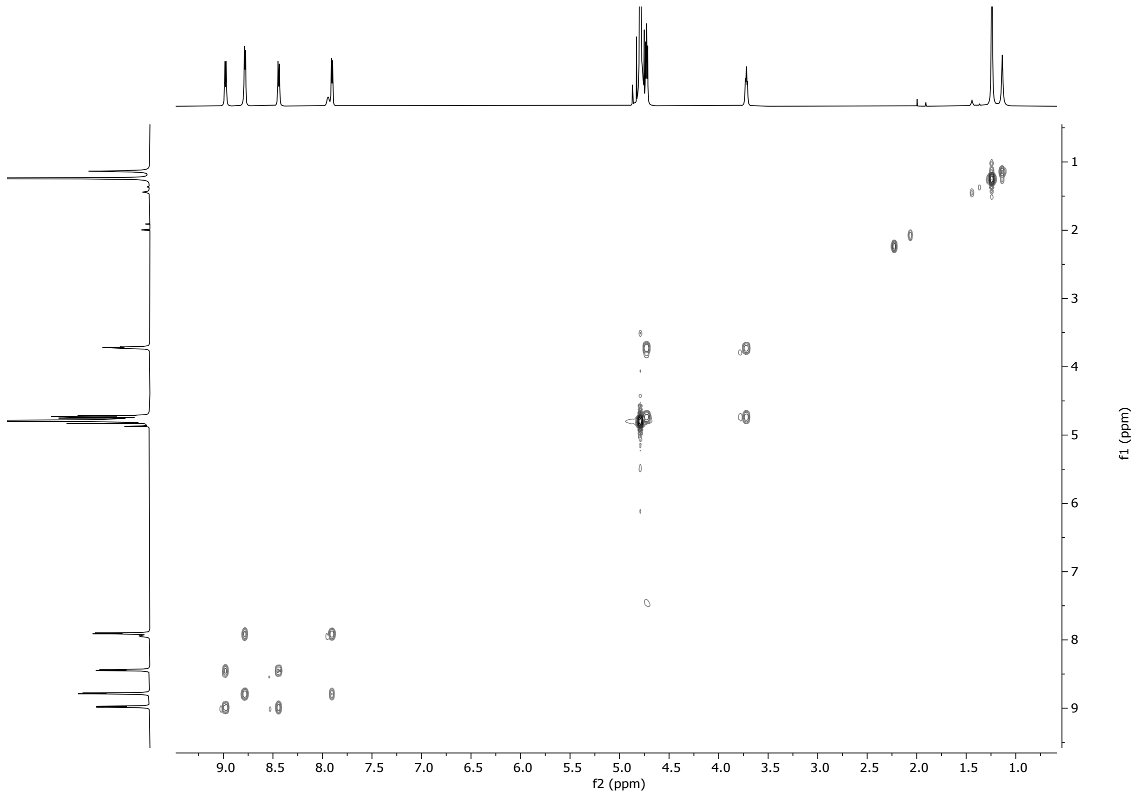

Figure S28: COSY (500 MHz, D_2O**) spectrum of 3·Cl.**

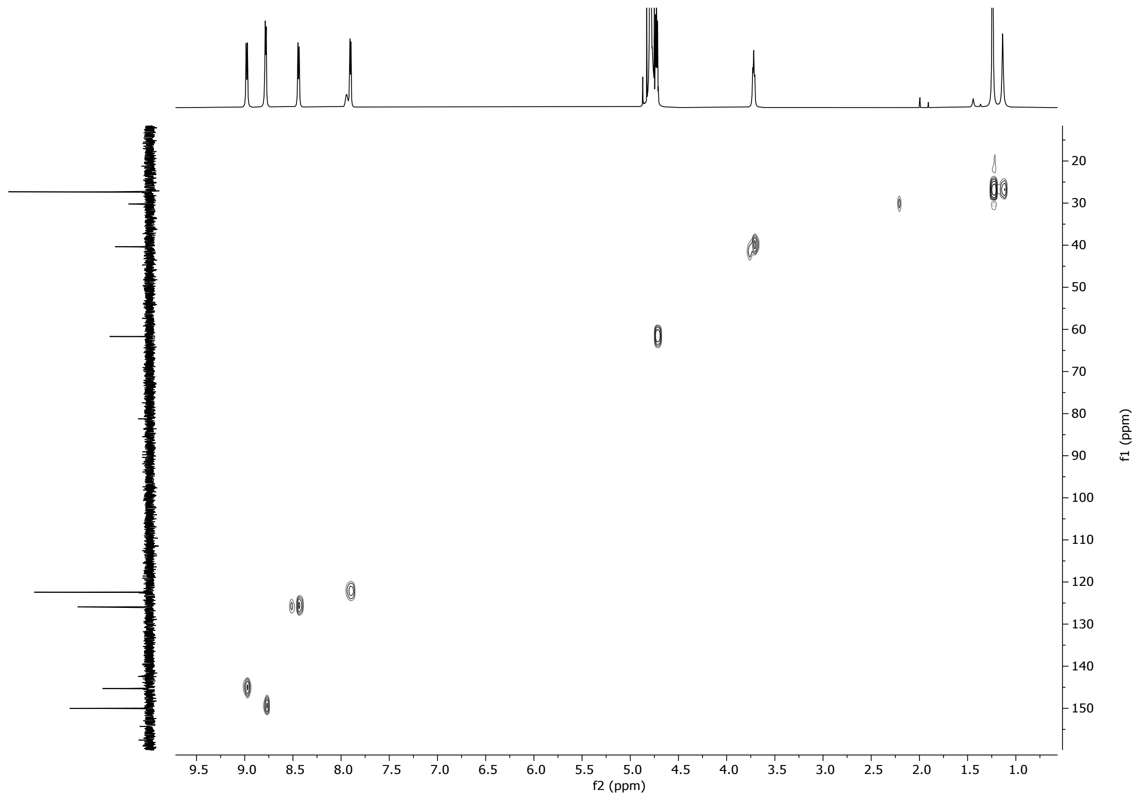

Figure S29: HSQC (500 MHz, D_2O) spectrum of 3·Cl.

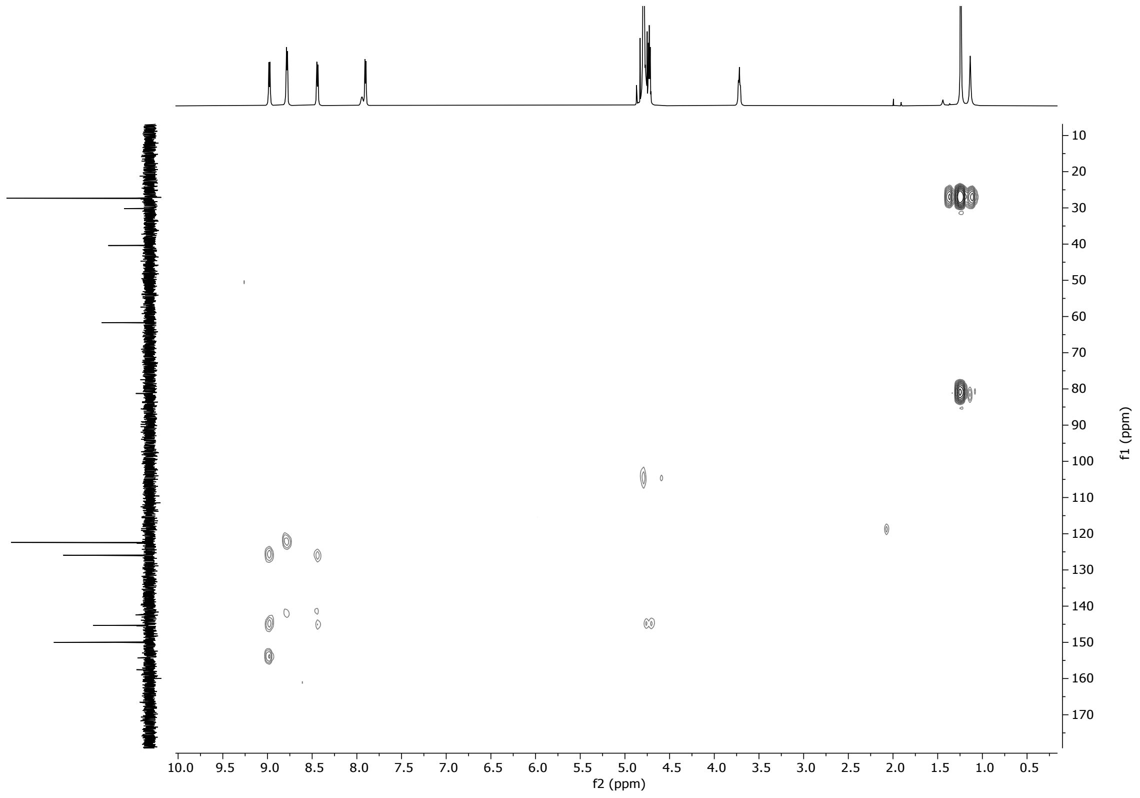

Figure S30: HMBC (500 MHz, D_2O) spectrum of 3·Cl.

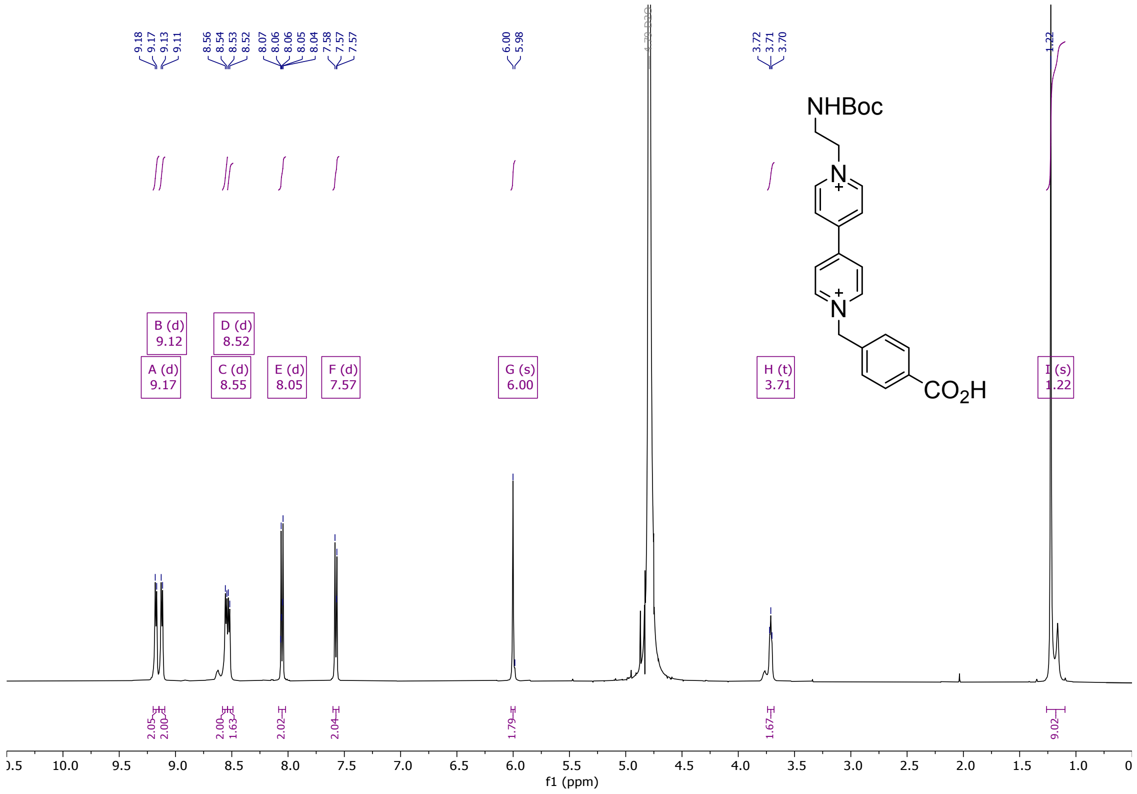

Figure S31: ^{1}H NMR (500 MHz, D_2O) spectrum of Boc-4·2Cl.

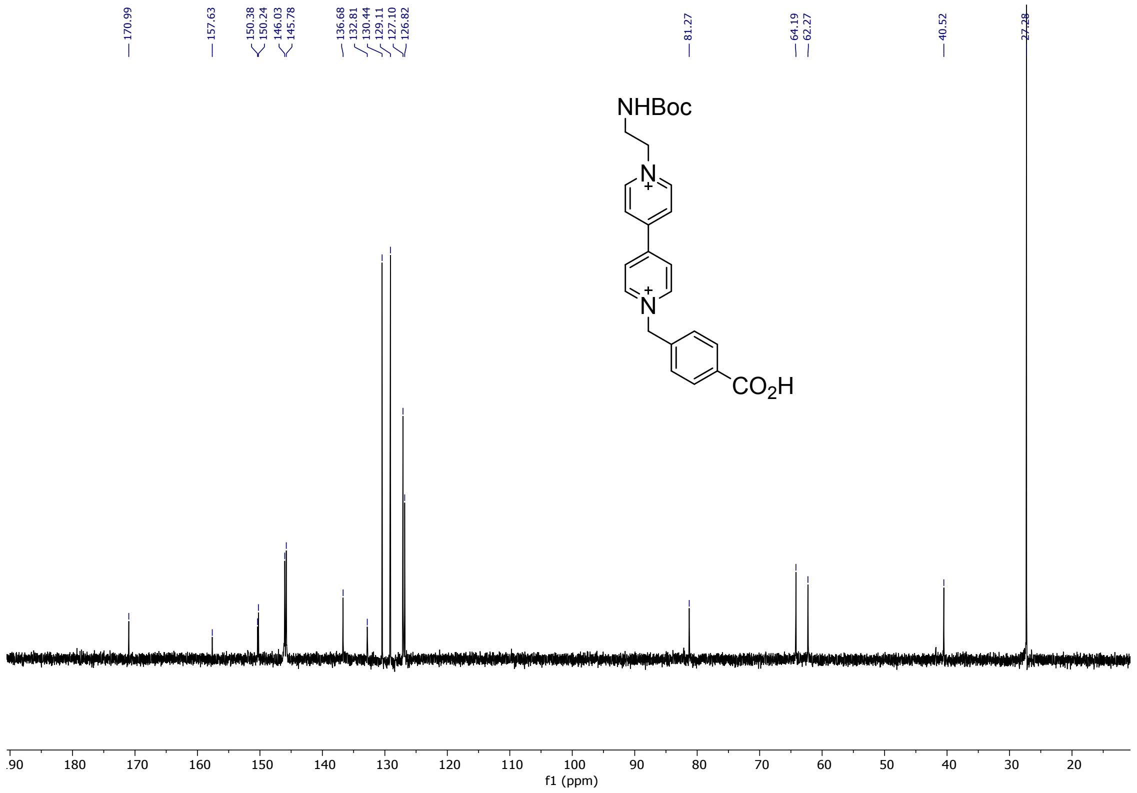

Figure S32: $^{13}C\{^{1}H\}$ NMR (125 MHz, D_2O) spectrum of Boc-4·2Cl.

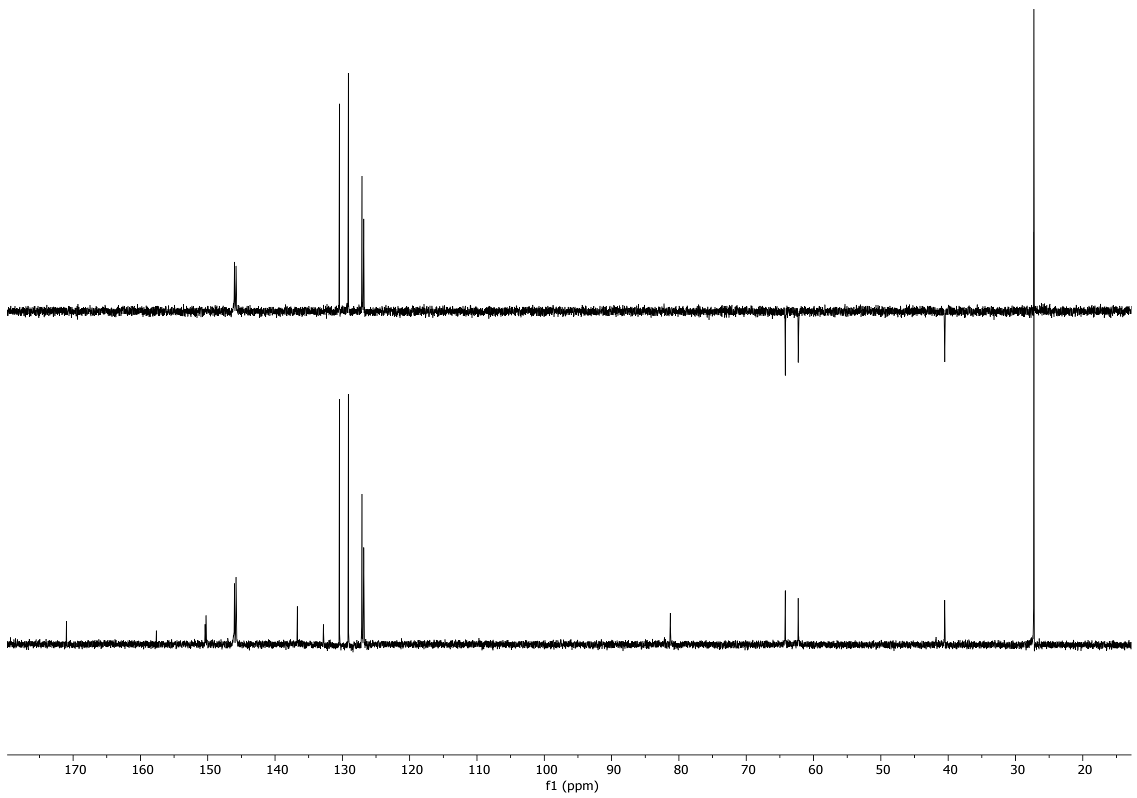

Figure S33: $^{13}C\{^1H\}$ NMR and DEPT (125 MHz, D_2O) spectrum of Boc-4·2Cl.

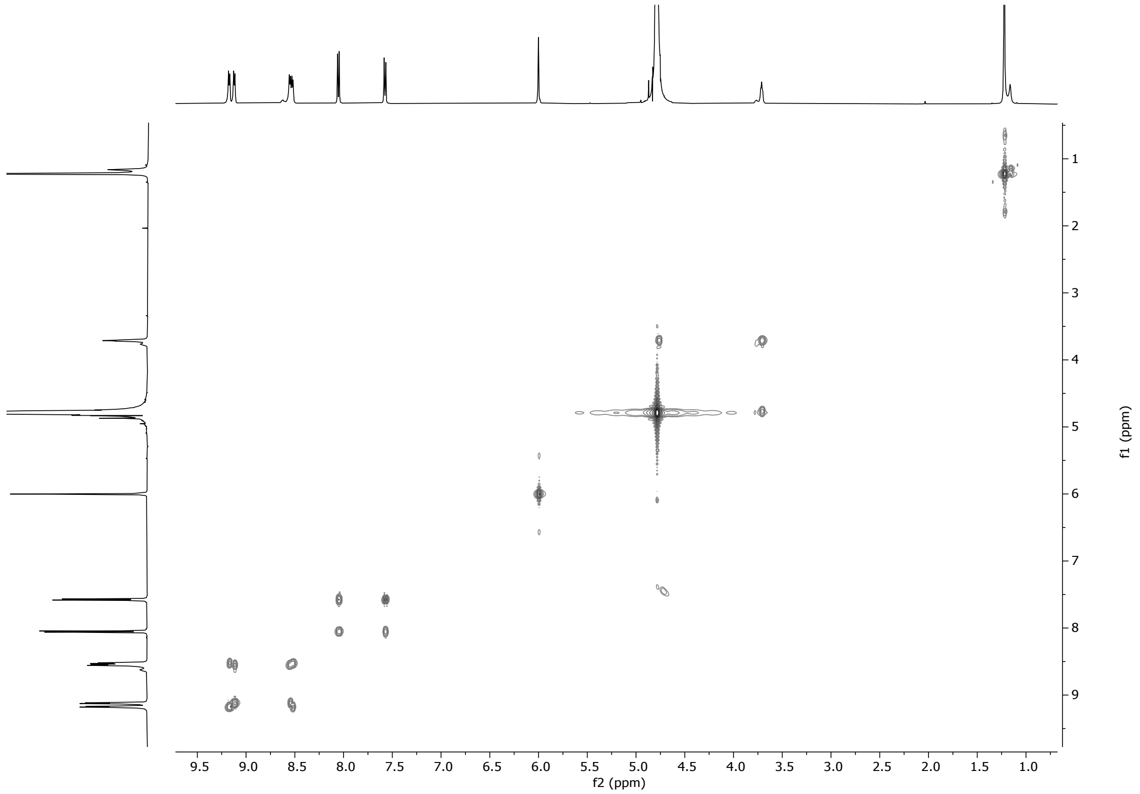

Figure S34: COSY (500 MHz, D_2O) spectrum of Boc-4·2Cl.

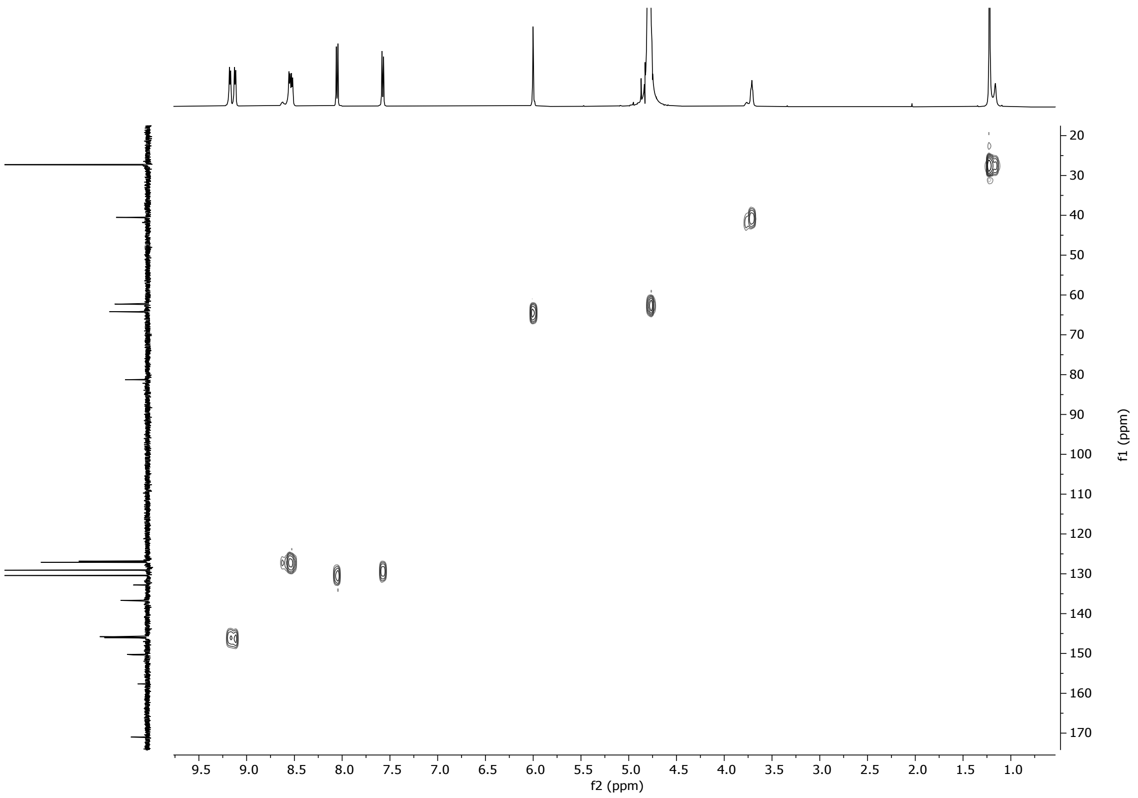

Figure S35: HSQC (500 MHz, D_2O) spectrum of Boc-4·2Cl.

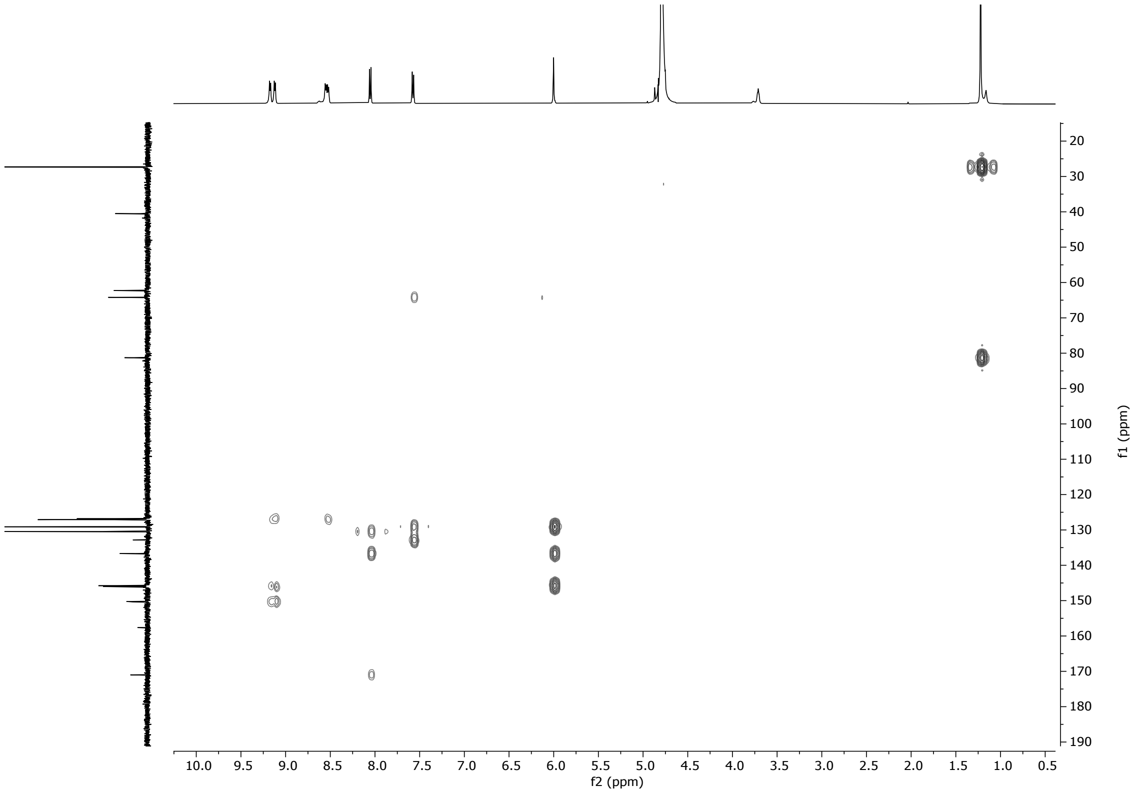

Figure S36: HMBC (500 MHz, D_2O) spectrum of Boc-4·2Cl.

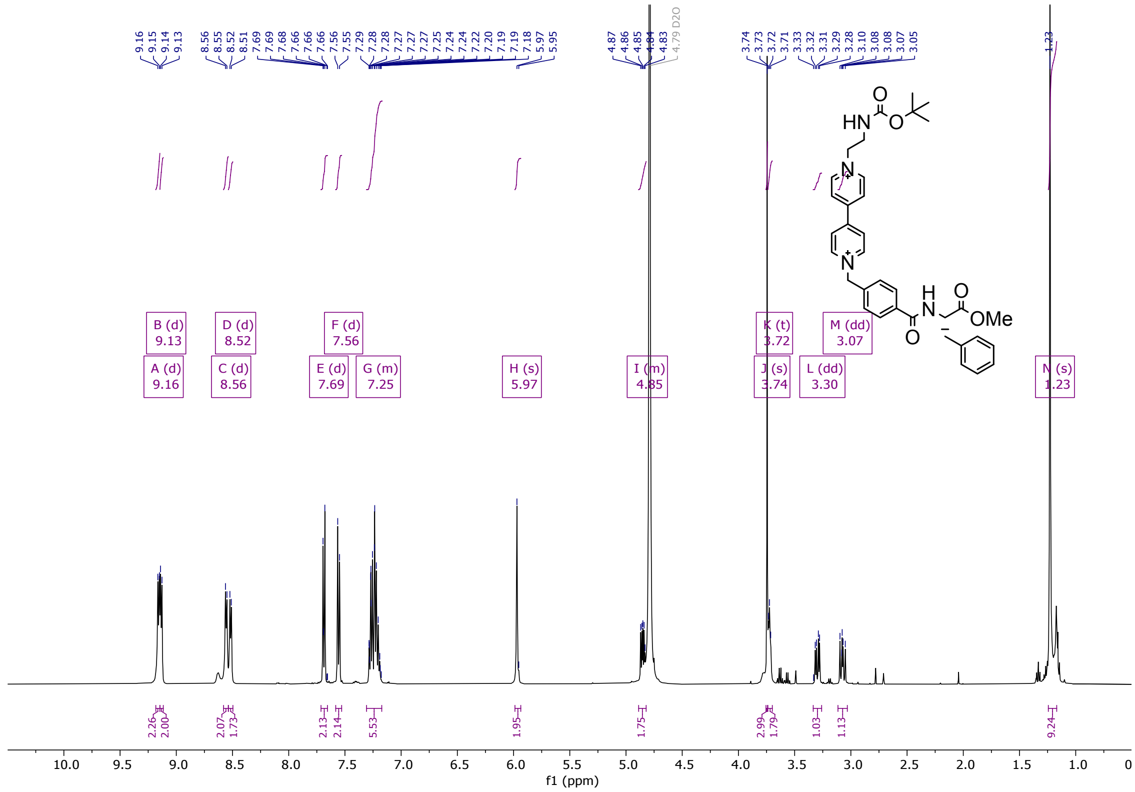

Figure S37: ^{1}H NMR (500 MHz, D_2O) spectrum of 5·2Cl.

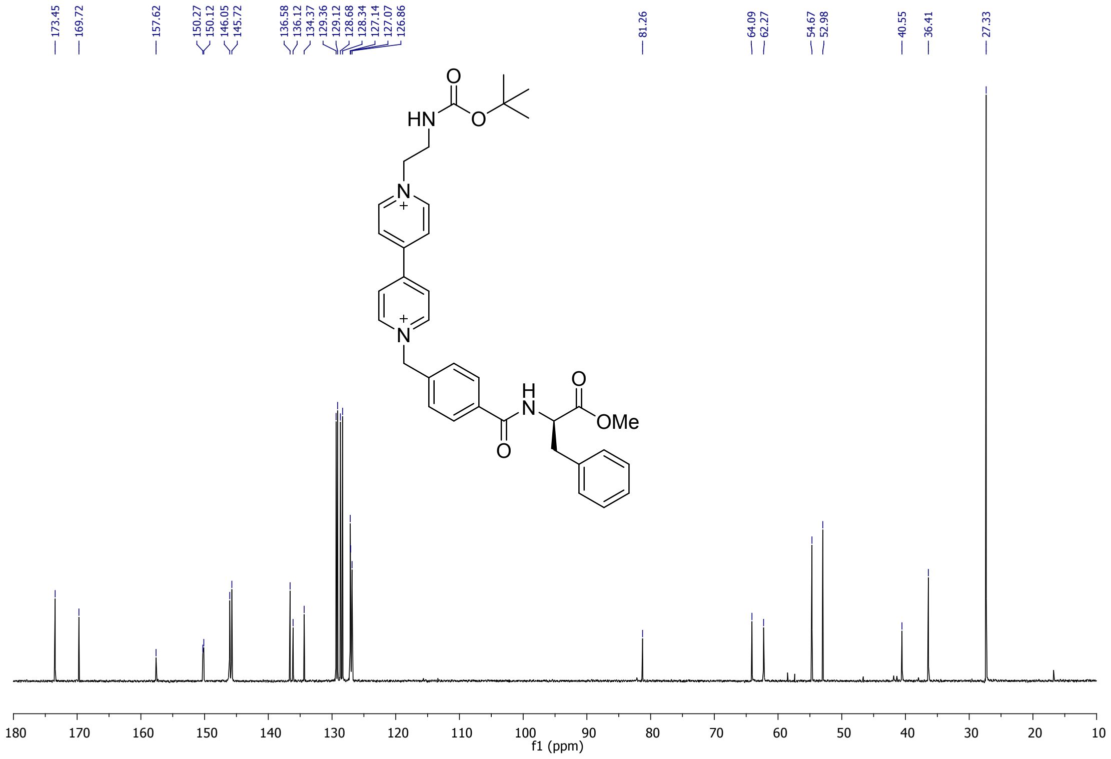

Figure S38: $^{13}C\{^{1}H\}$ NMR (125 MHz, D_2O) spectrum of 5·2Cl.

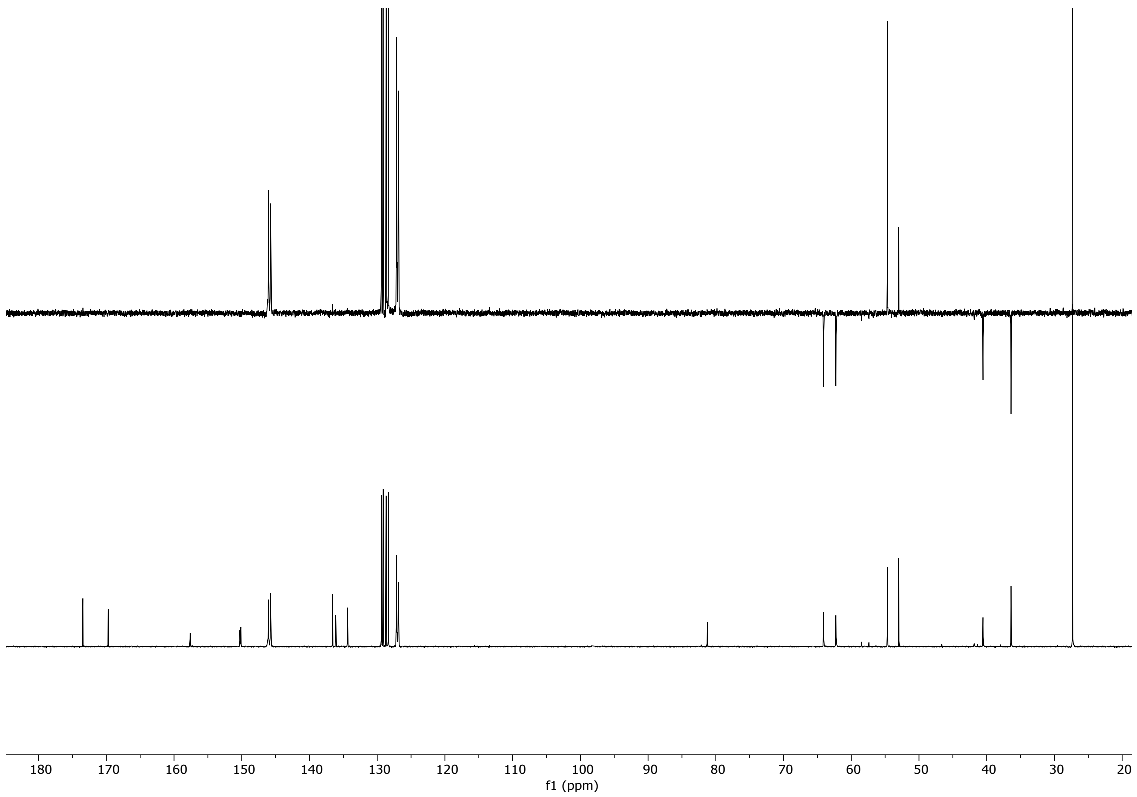

Figure S39: $^{13}C\{^1H\}$ NMR and DEPT (125 MHz, D_2O) spectrum of 5·2Cl.

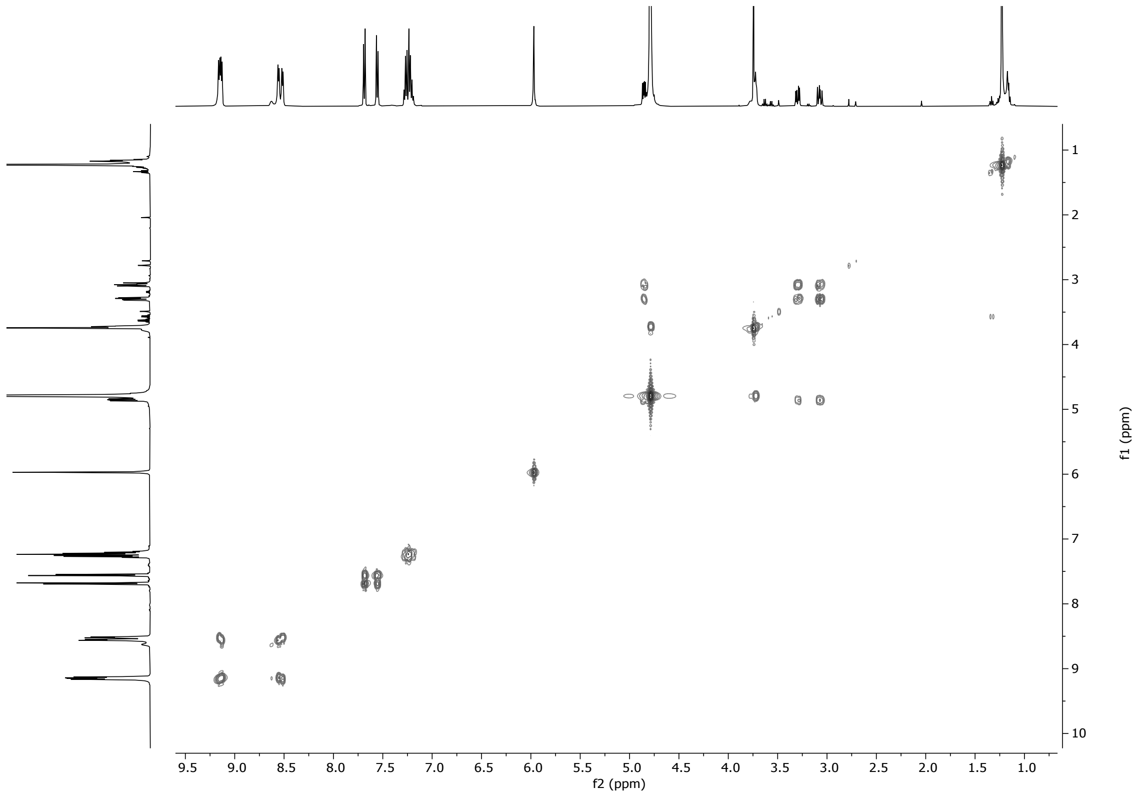

Figure S40: COSY (500 MHz, D_2O) spectrum of 5·2Cl.

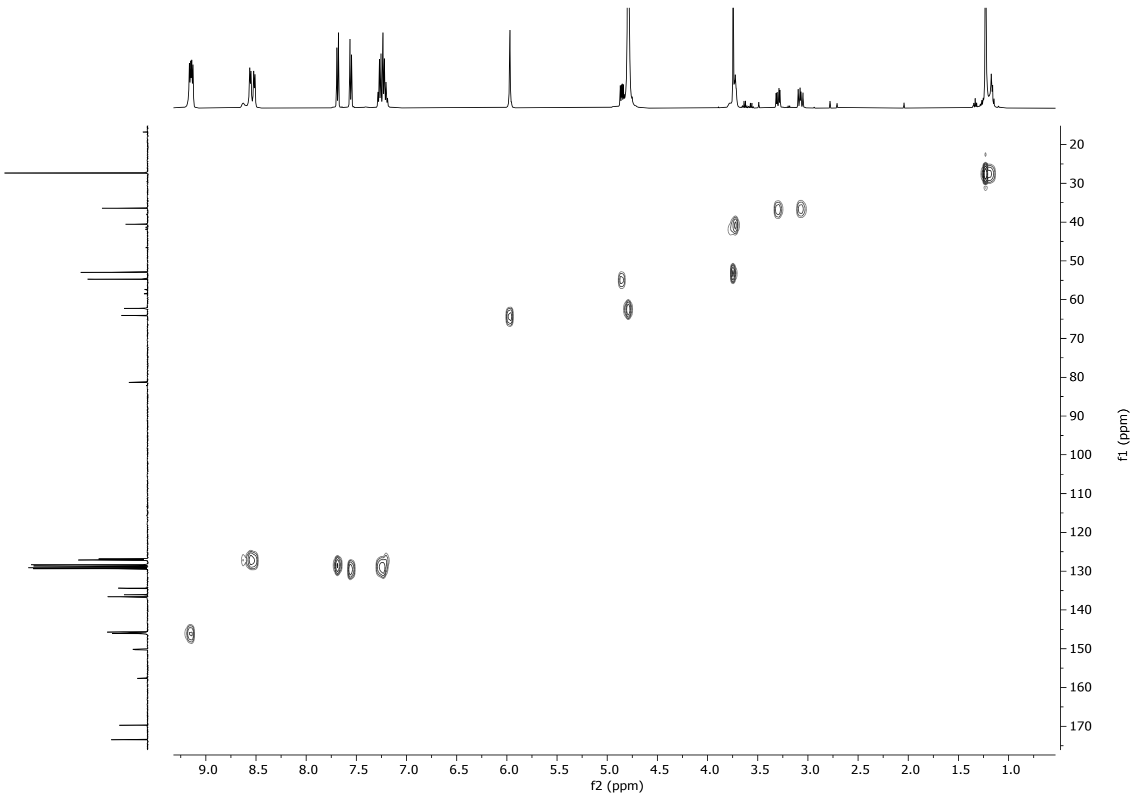

Figure S41: HSQC (500 MHz, D_2O) spectrum of 5·2Cl.

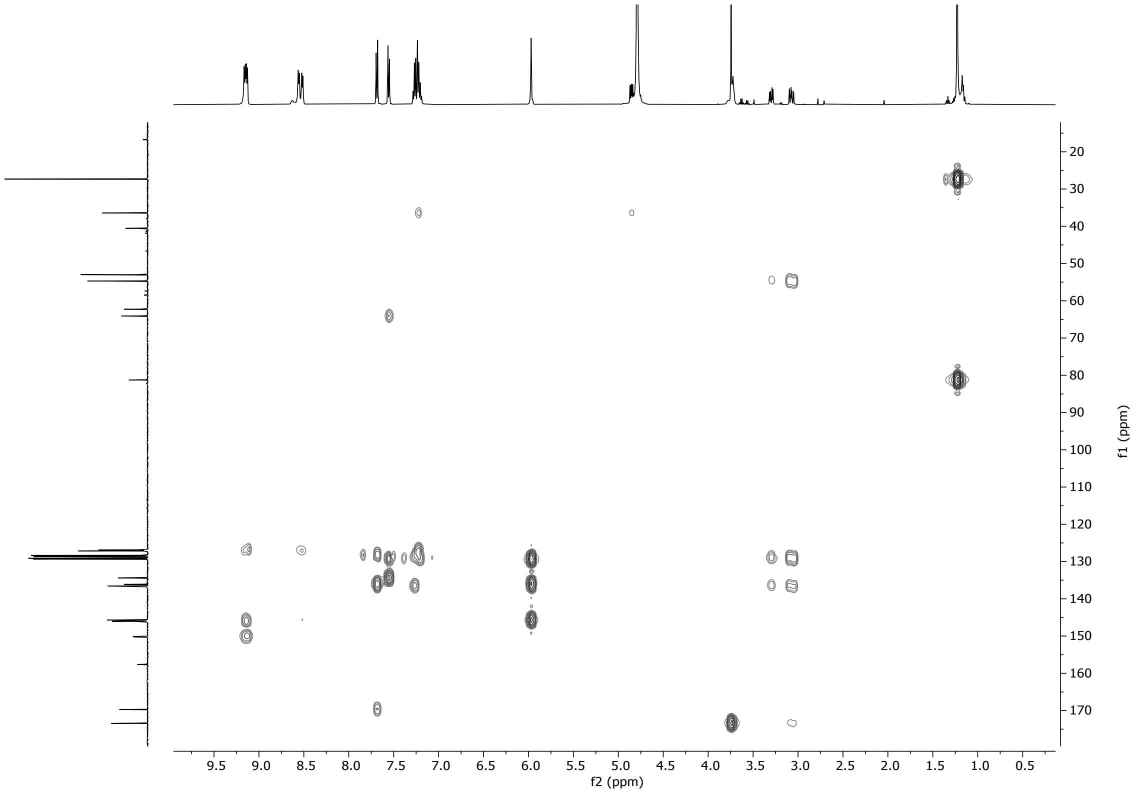

Figure S42: HMBC (500 MHz, D_2O) spectrum of 5·2Cl.

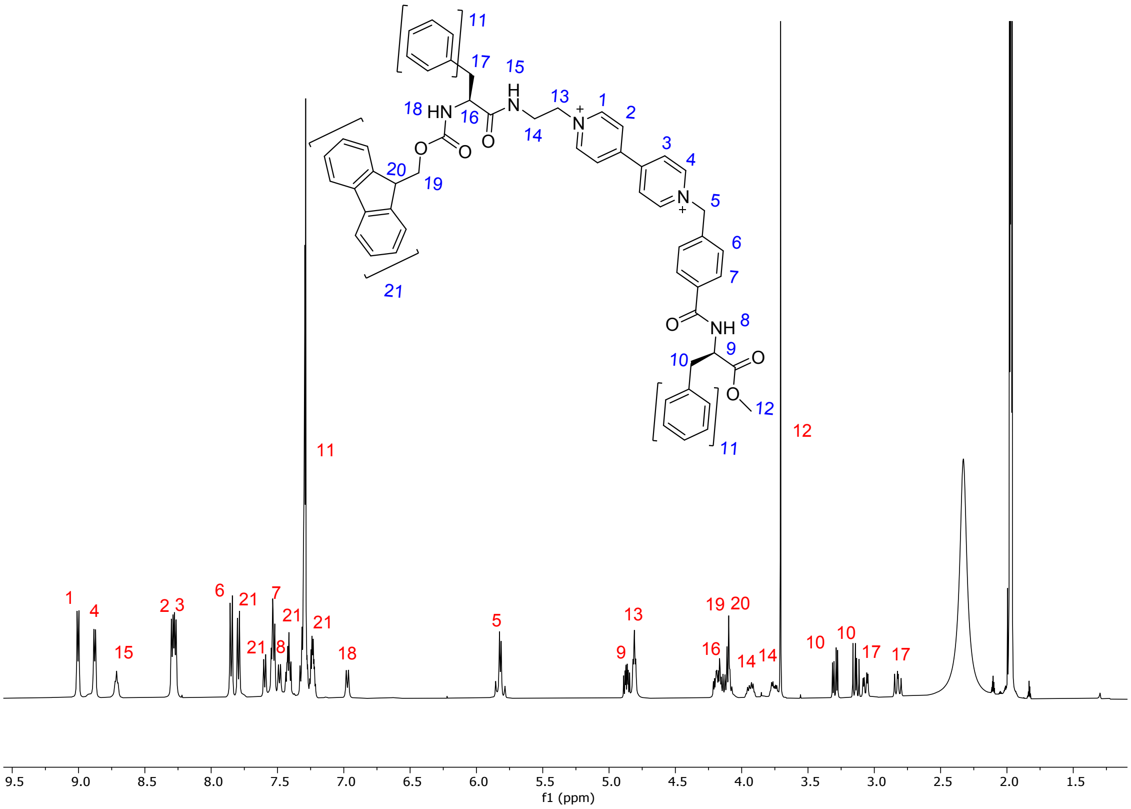

Figure S43: ^{1}H-NMR (500 MHz, CD_3CN) assignment of 7·2TFA.

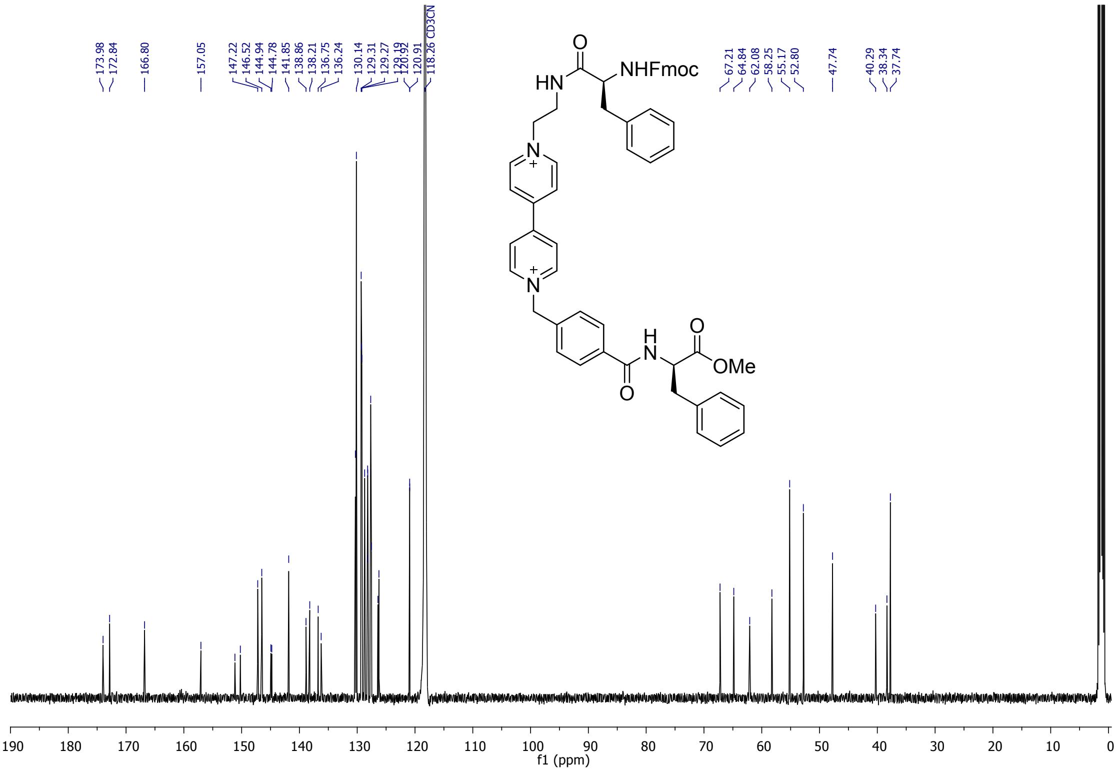

Figure S44: $^{13}C\{^{1}H\}$ NMR (125 MHz, CD_3CN) spectrum of 7·2TFA.

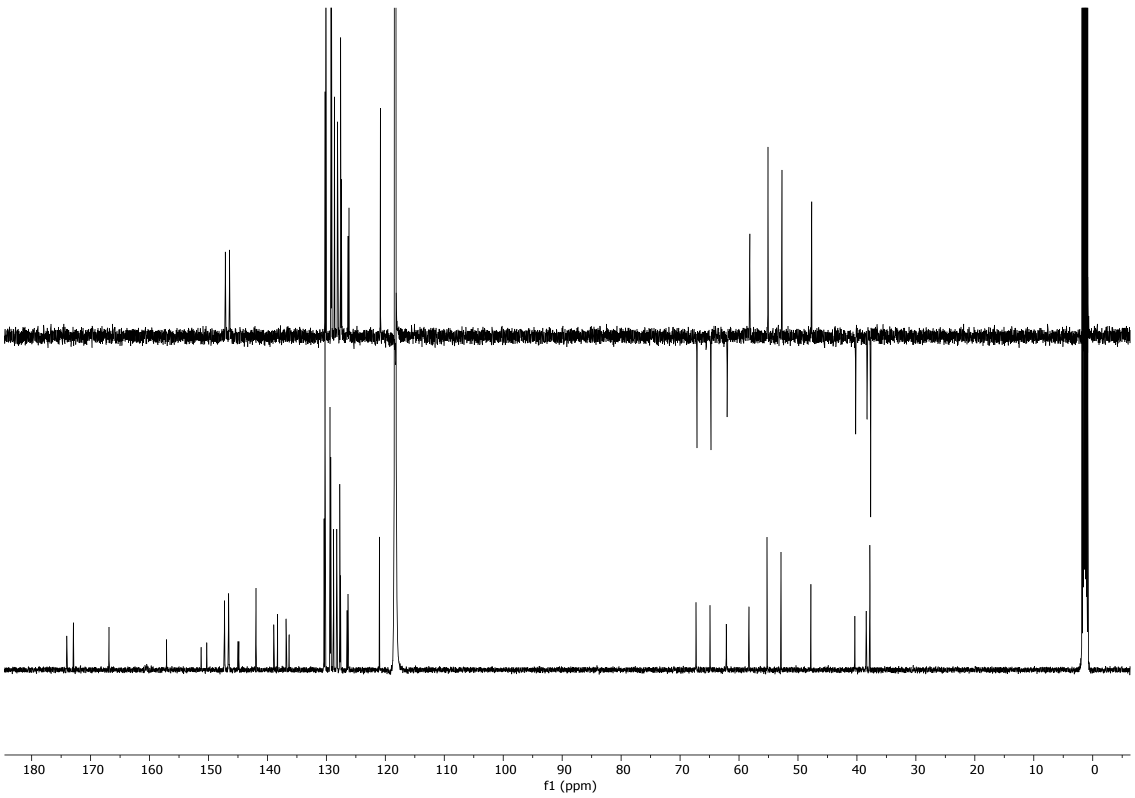

Figure S45: **$^{13}C\{^1H\}$ NMR and DEPT (125 MHz, CD_3CN) spectrum of 7·2TFA.**

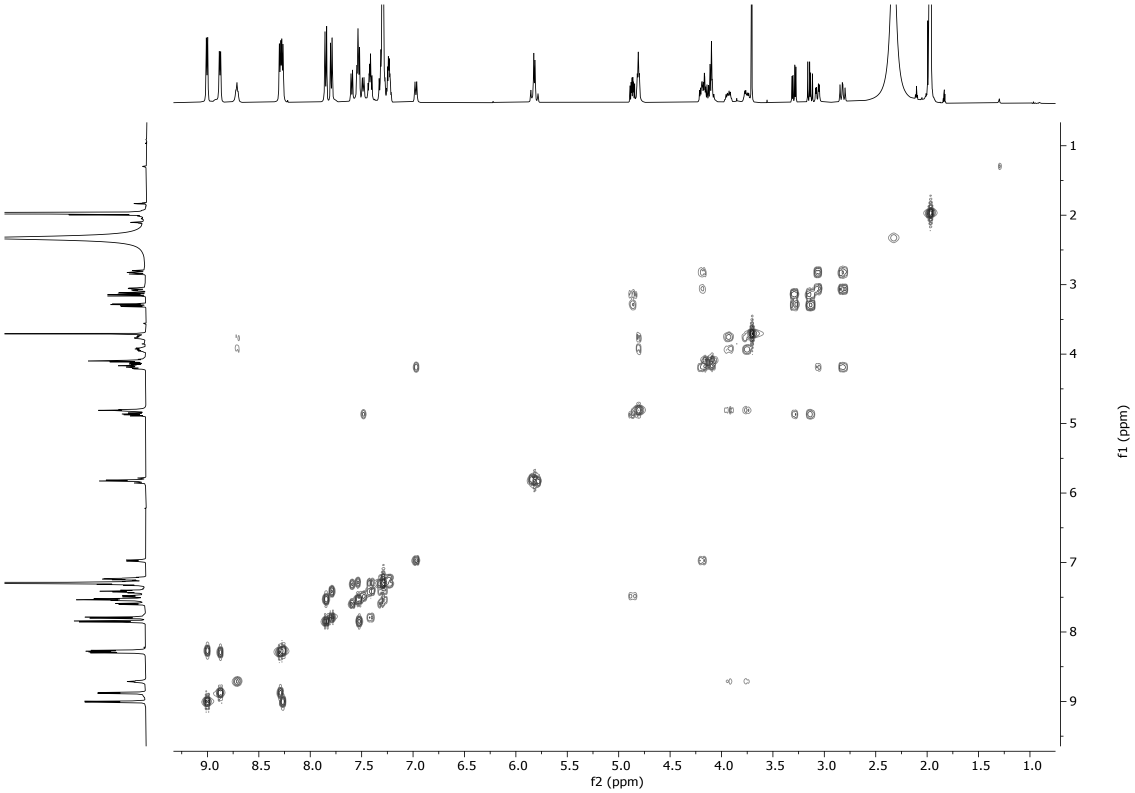

Figure S46: COSY (500 MHz, CD_3CN) spectrum of 7·2TFA.

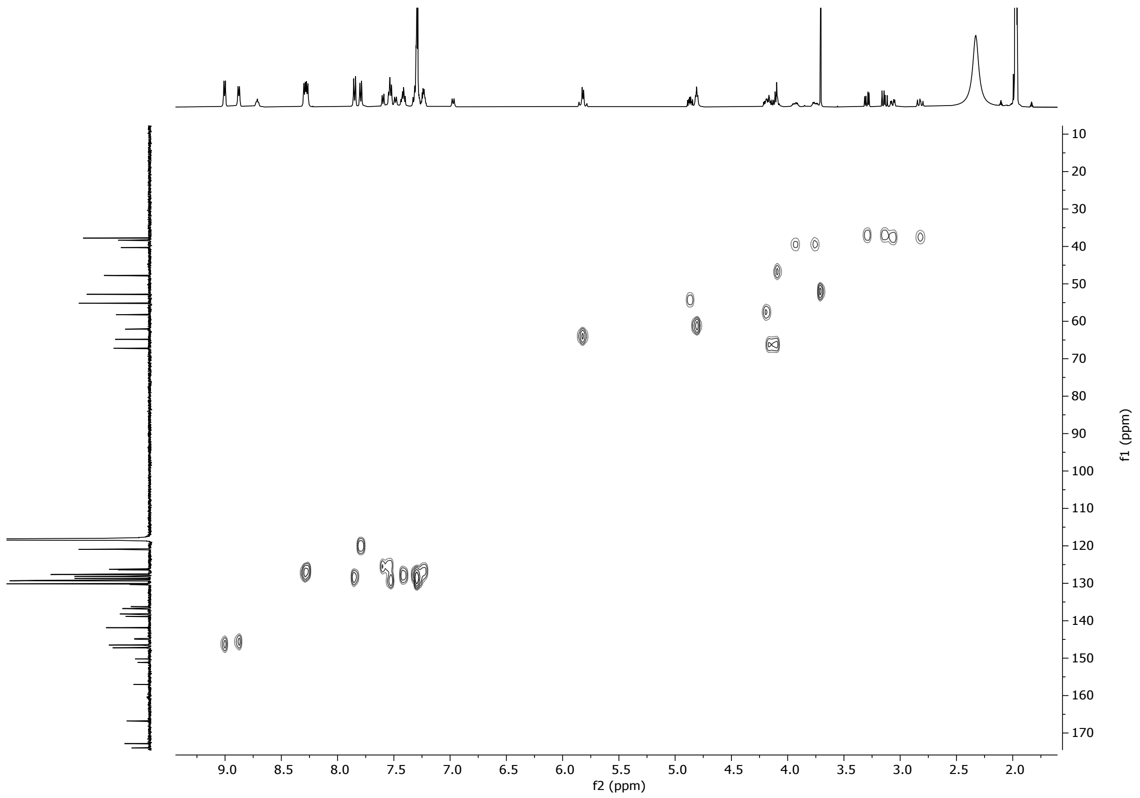

Figure S47: HSQC (500 MHz, CD_3CN) spectrum of 7·2TFA.

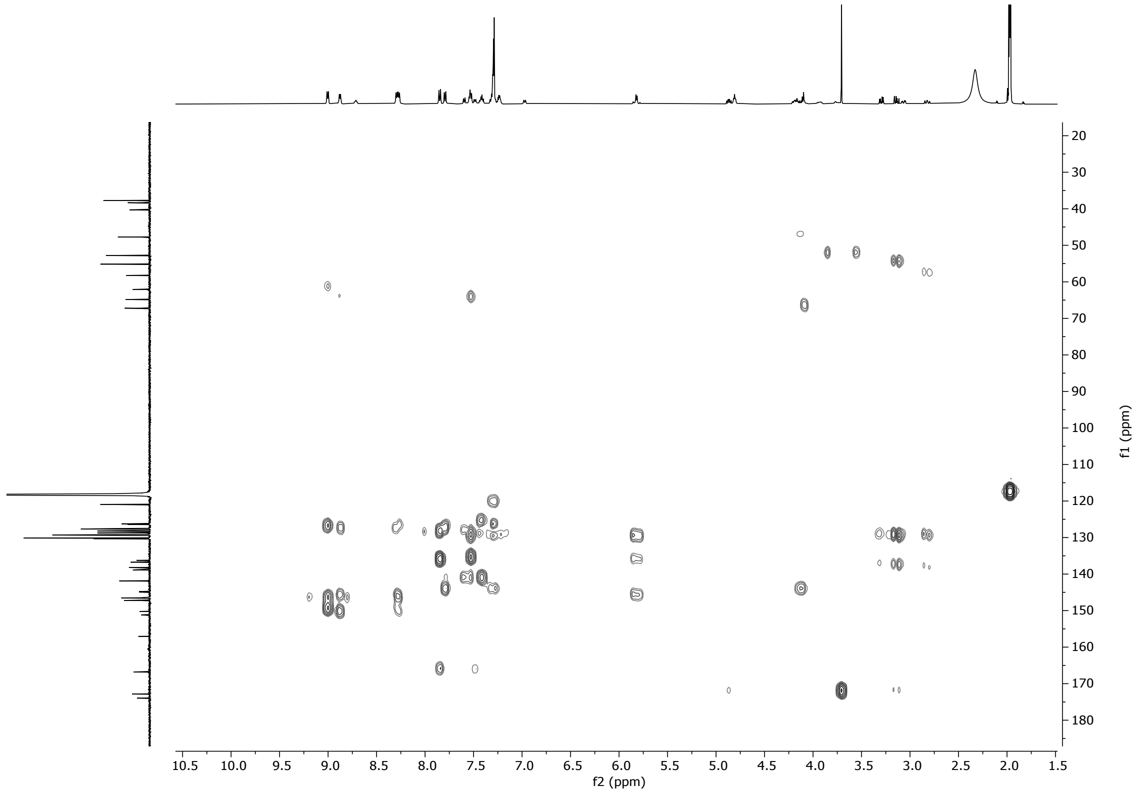

Figure S48: HMBC (500 MHz, CD_3CN) spectrum of 7·2TFA.

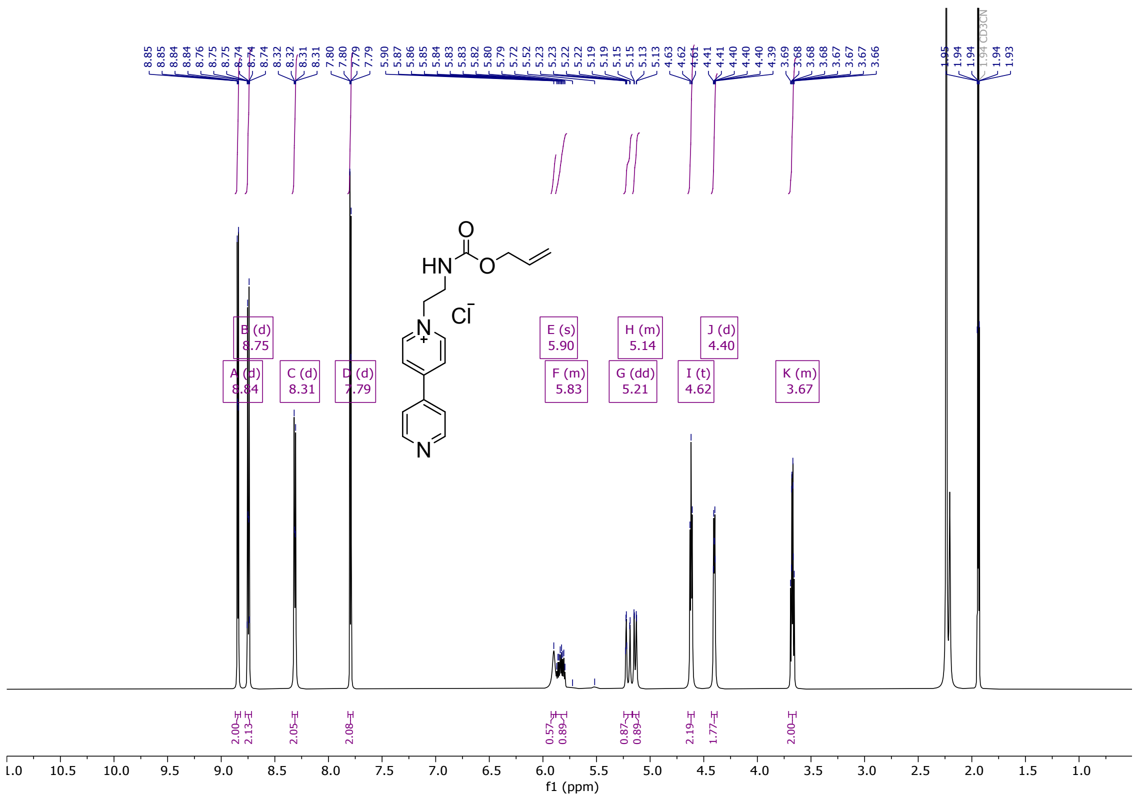

Figure S49: ^{1}H NMR (500 MHz, CD_3CN) spectrum of 8·Cl.

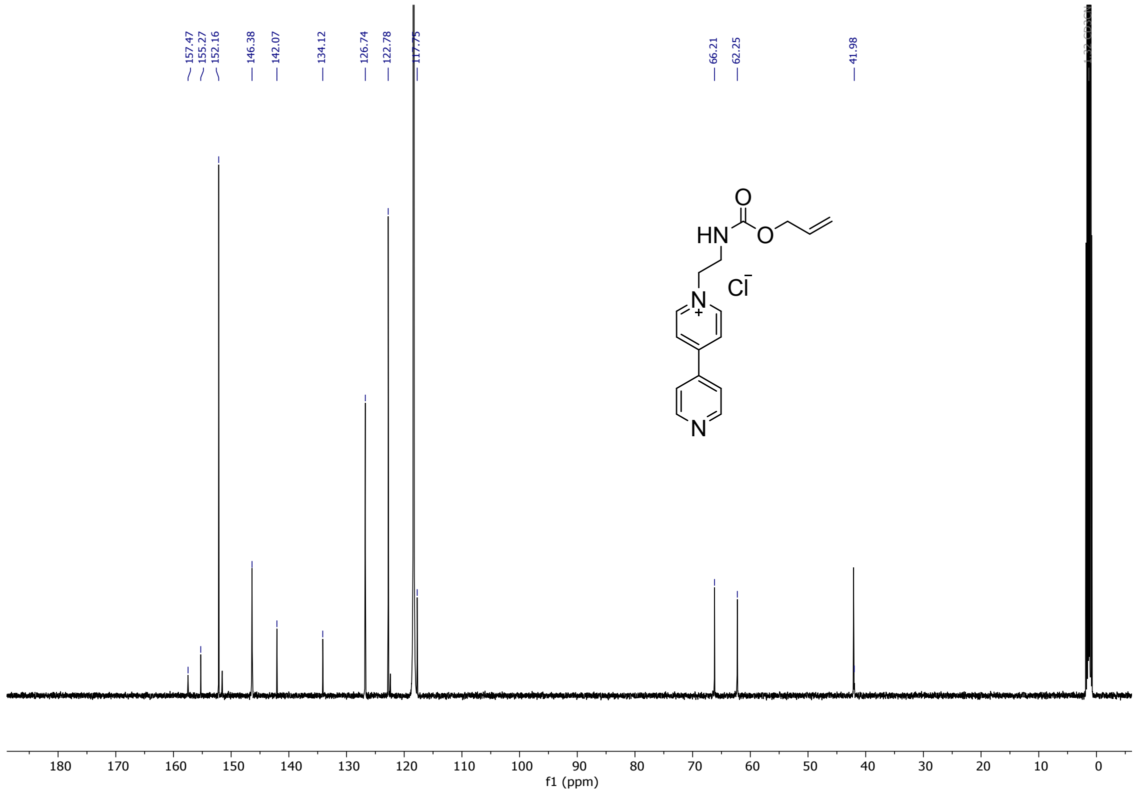

Figure S50: $^{13}C\{^{1}H\}$ NMR (125 MHz, CD_3CN) spectrum of 8·Cl.

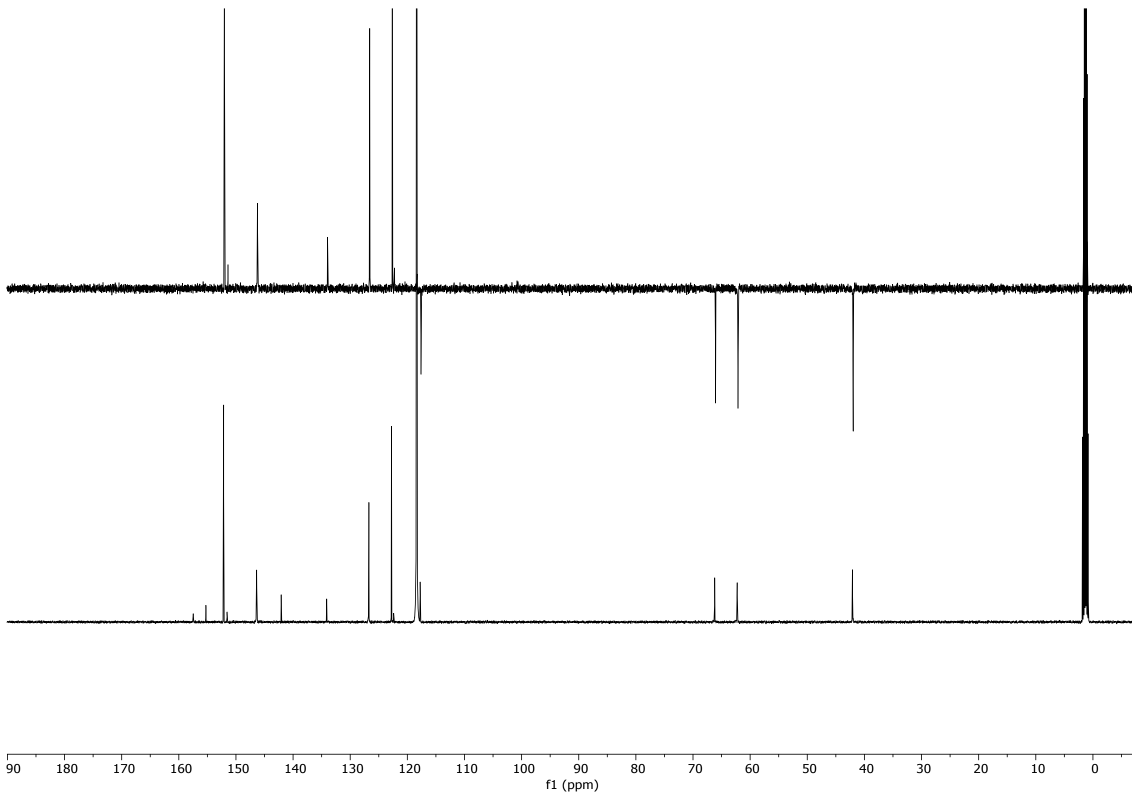

Figure S51: $^{13}C\{^{1}H\}$ NMR and DEPT (125 MHz, CD_3CN) spectrum of 8·Cl.

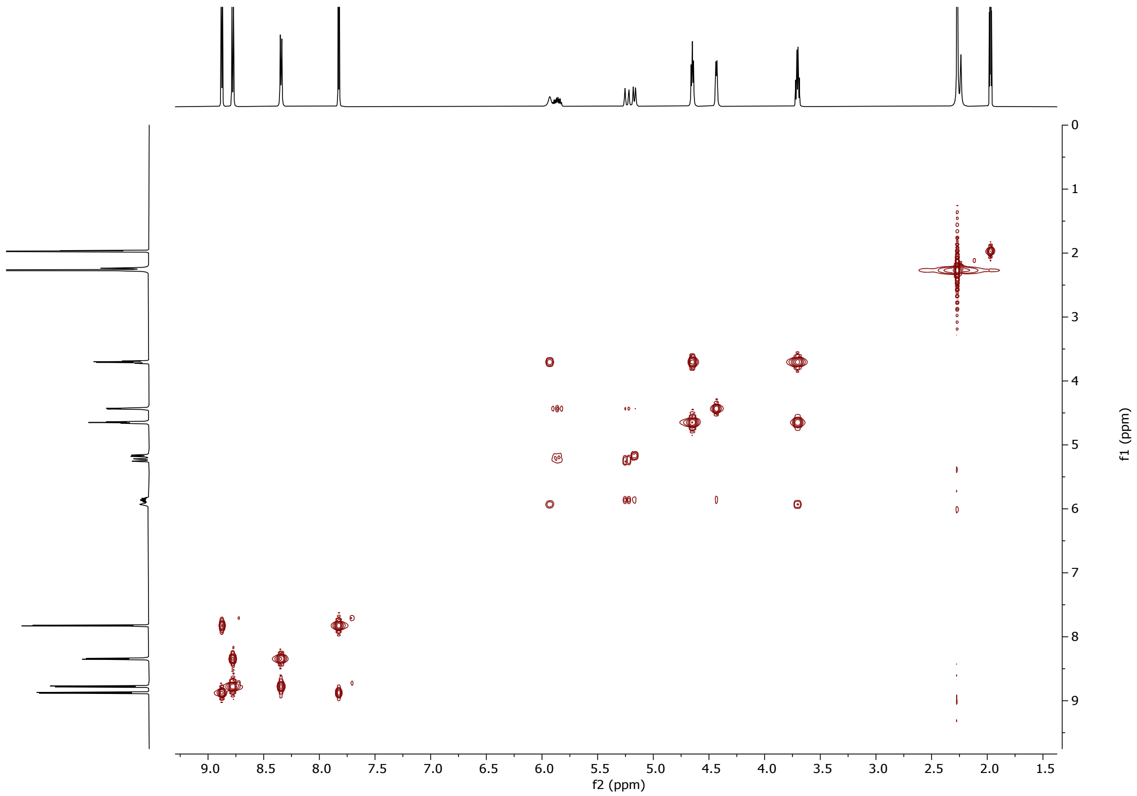

Figure S52: COSY (500 MHz, CD_3CN) spectrum of 8·Cl.

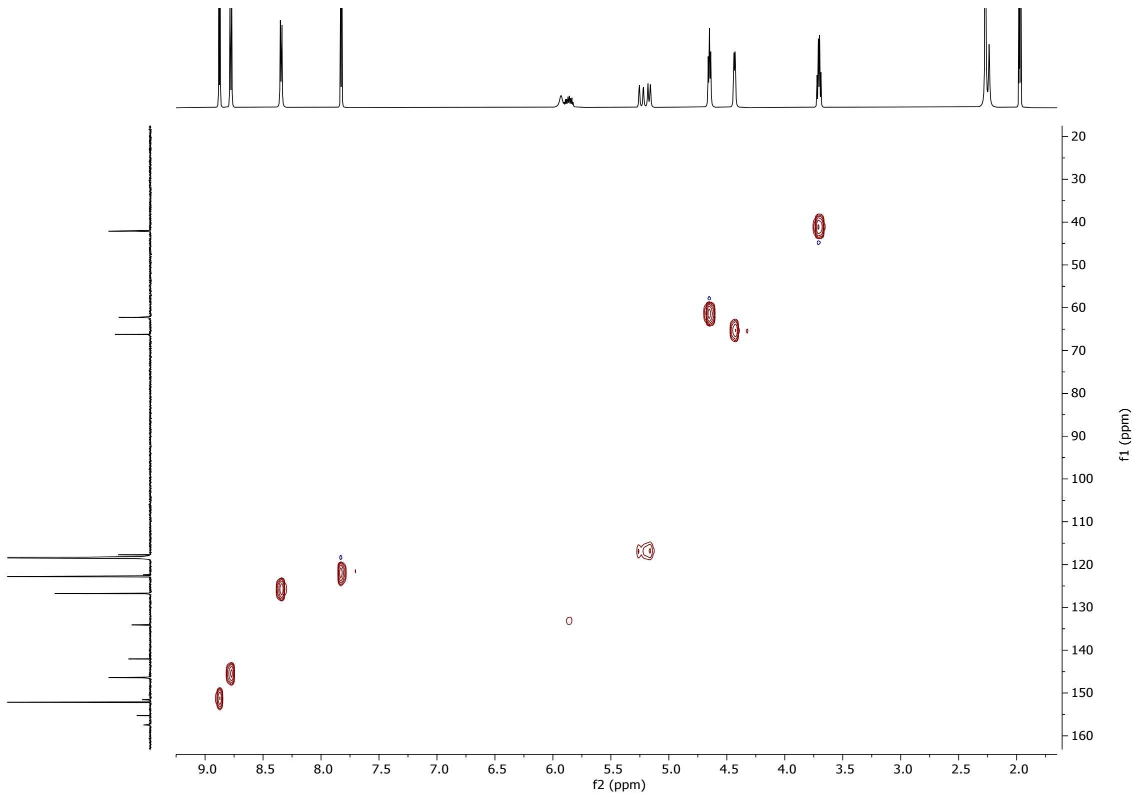

Figure S53: HSQC (500 MHz, CD_3CN) spectrum of 8·Cl.

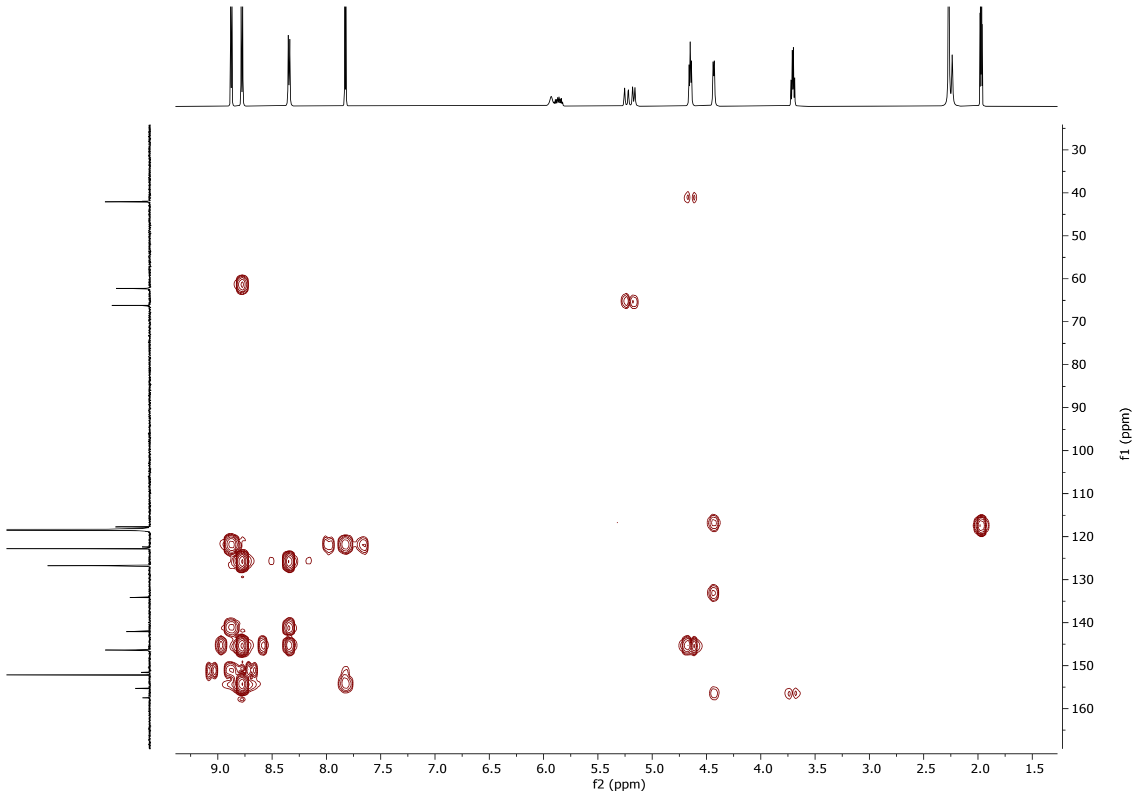

Figure S54: HMBC (500 MHz, CD_3CN) spectrum of 8·Cl.

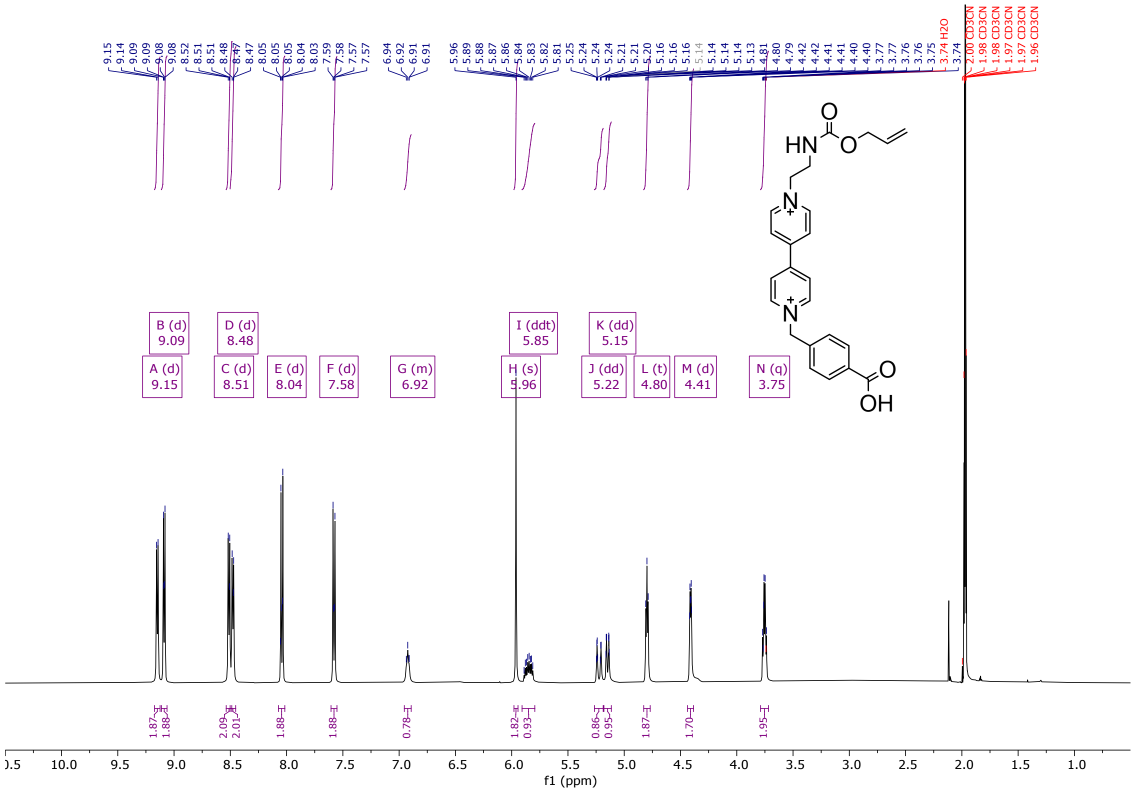

Figure S55: ^{1}H NMR (500 MHz, D_2O) spectrum of Alloc-4·2TFA.

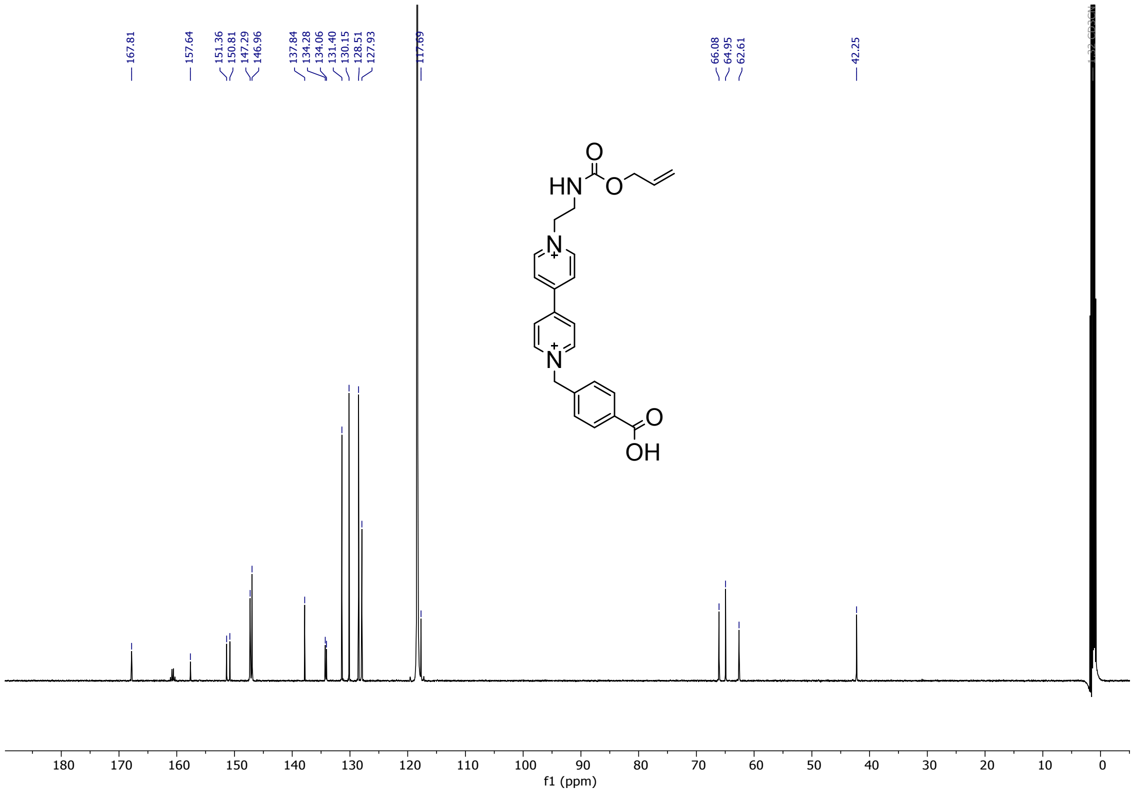

Figure S56: $^{13}C\{^{1}H\}$ NMR (125 MHz, D_2O) spectrum of Alloc-4·2TFA.

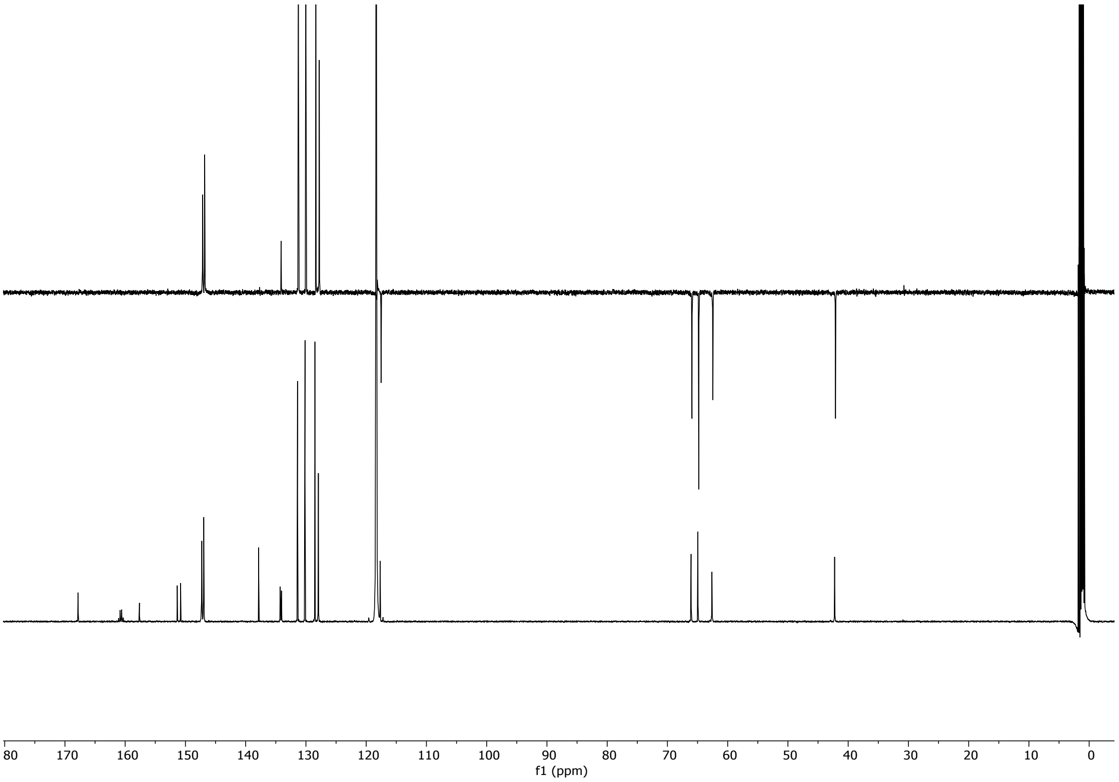

Figure S57: $^{13}C\{^{1}H\}$ NMR and DEPT (125 MHz, D_2O) spectrum of Alloc-4·2TFA.

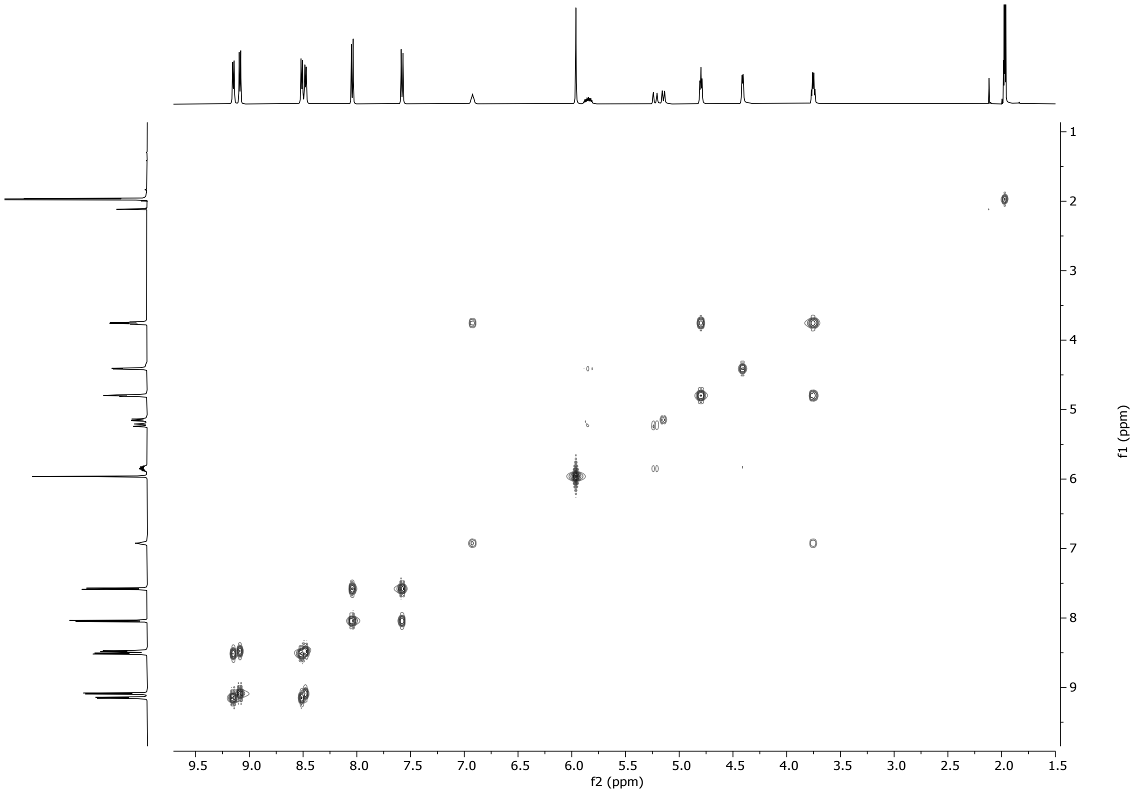

Figure S58: COSY (500 MHz, D_2O) spectrum of Alloc-4·2TFA.

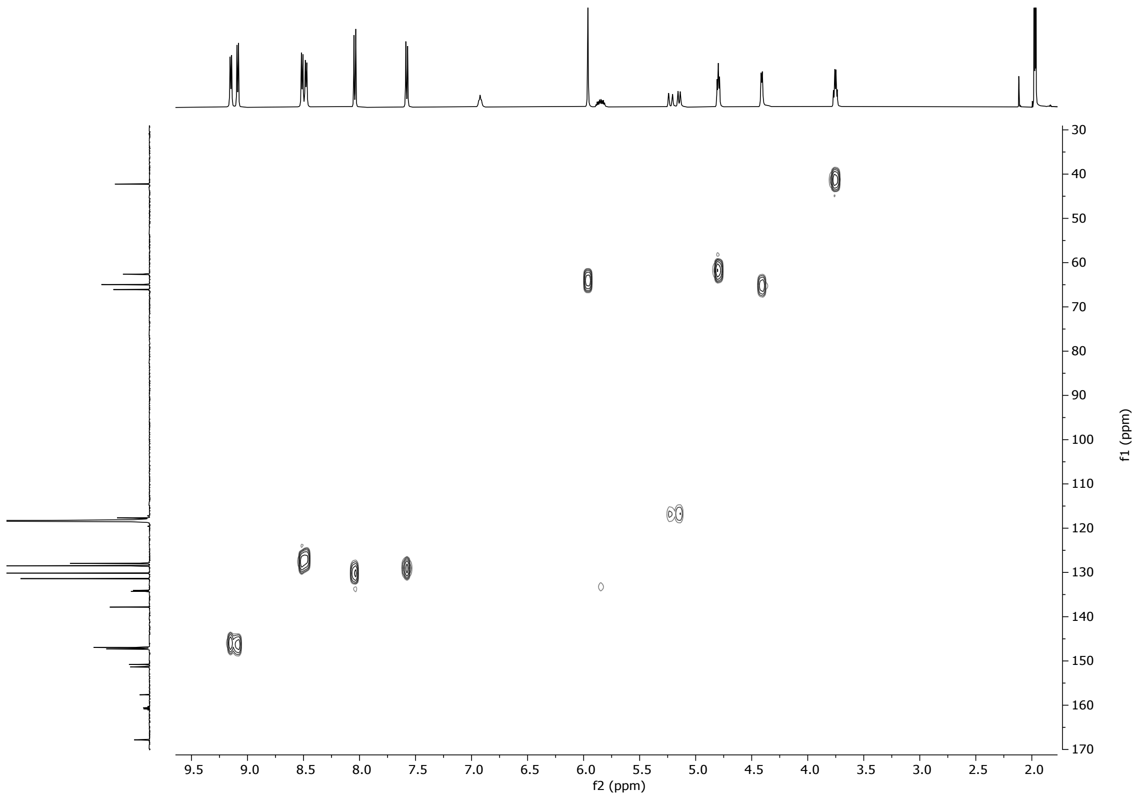

Figure S59: HSQC (500 MHz, D_2O) spectrum of Alloc-4·2TFA.

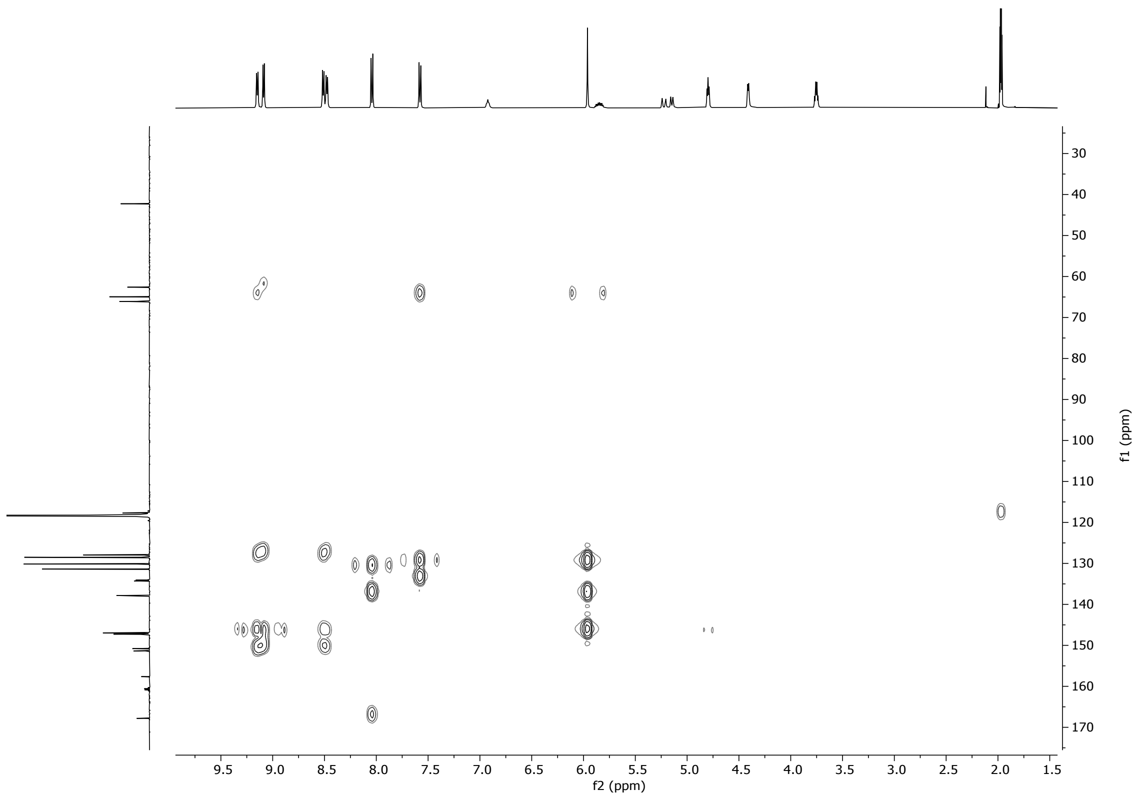

Figure S60: HMBC (500 MHz, D_2O) spectrum of Alloc-4·2TFA.

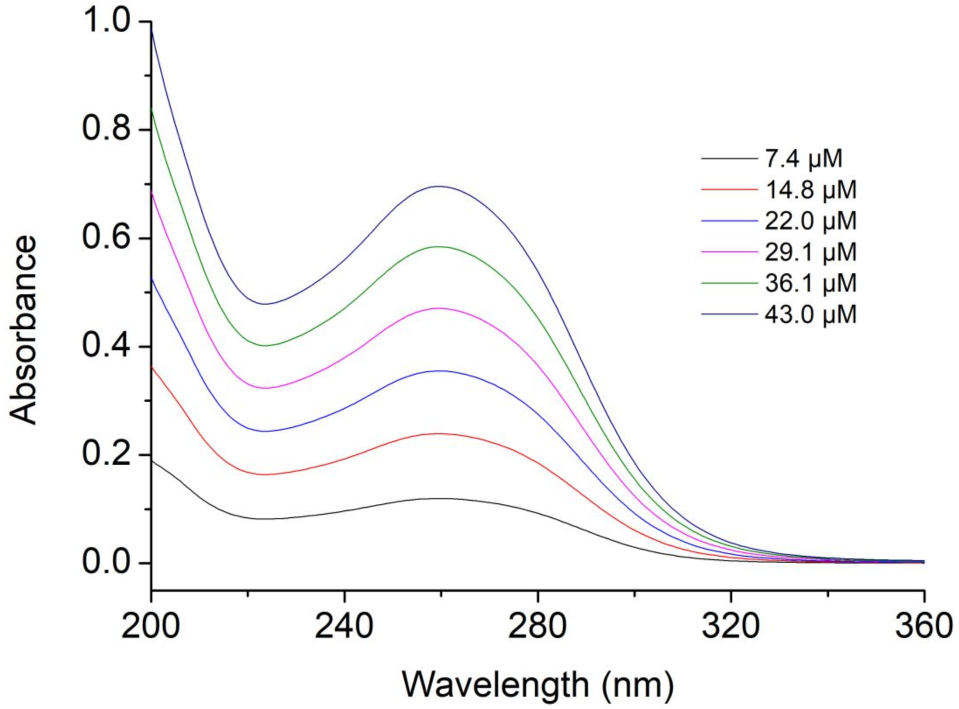

Figure S61: UV-Vis spectra of Alloc-4·2Cl in 50mM buffer solution, pH 7 from 7.4 μM to 43.0 μM.

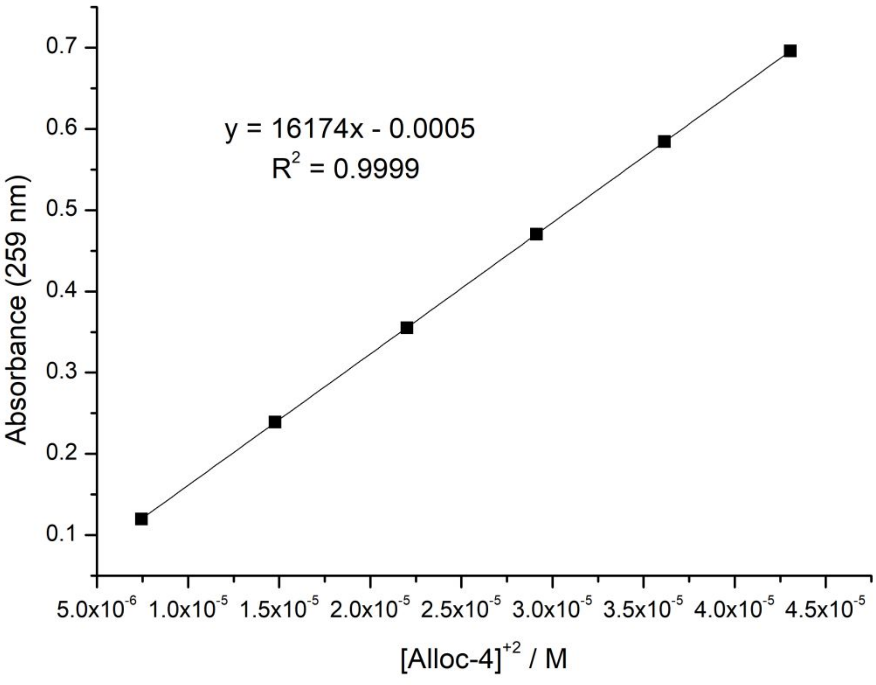

Figure S62: Linear relationship between the absorbance at 259 nm and the concentration of Alloc-4^{+2} where ε=16174 Lmol^{-1}cm^{-1}.

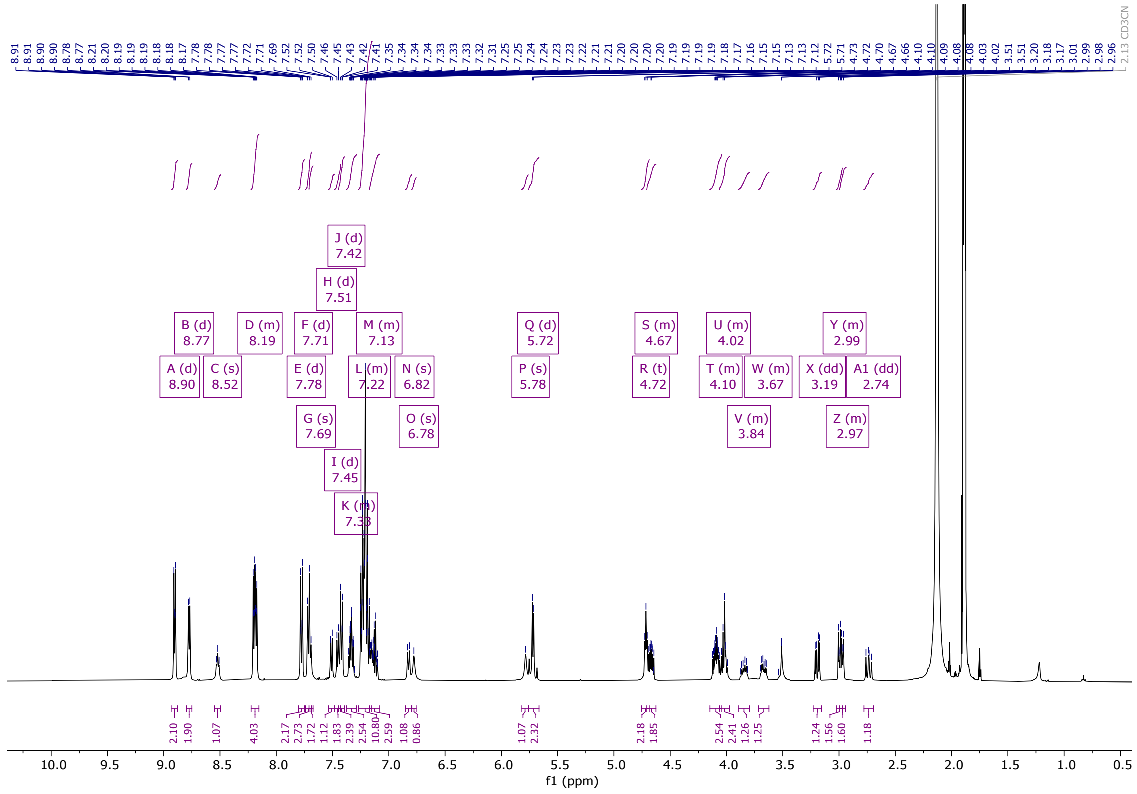

Figure S63: ^{1}H NMR (500 MHz, CD_3CN) spectrum of 9·2TFA.

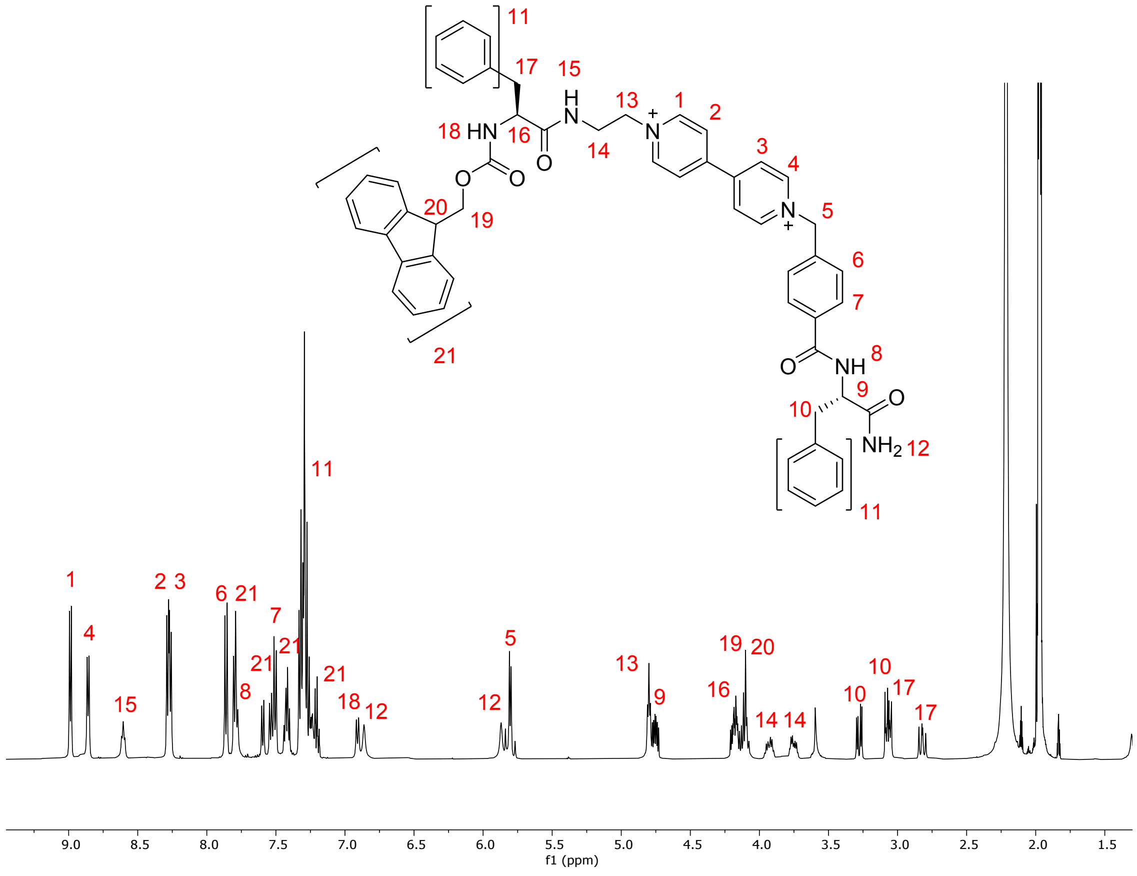

Figure S64: ^{1}H-NMR (500 MHz, CD_3CN) assignment of 9·2TFA.

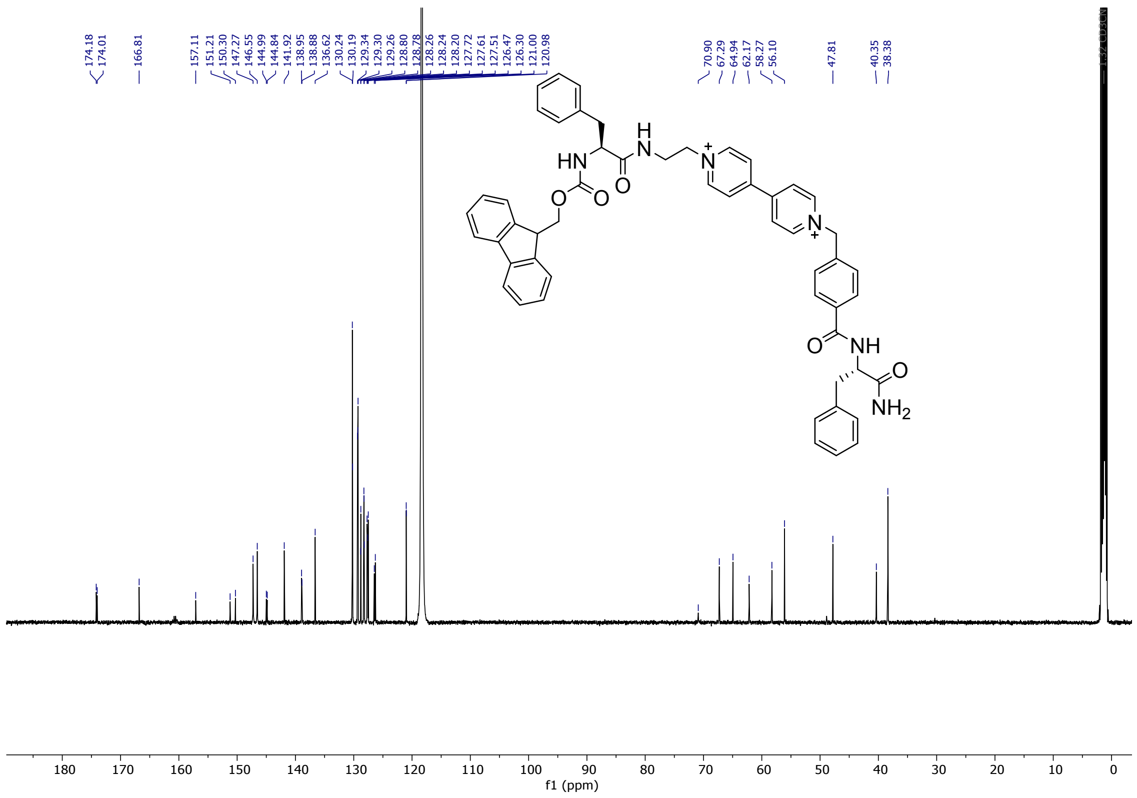

Figure S65: $^{13}C\{^{1}H\}$ NMR (125 MHz, CD_3CN) spectrum of 9·2TFA.

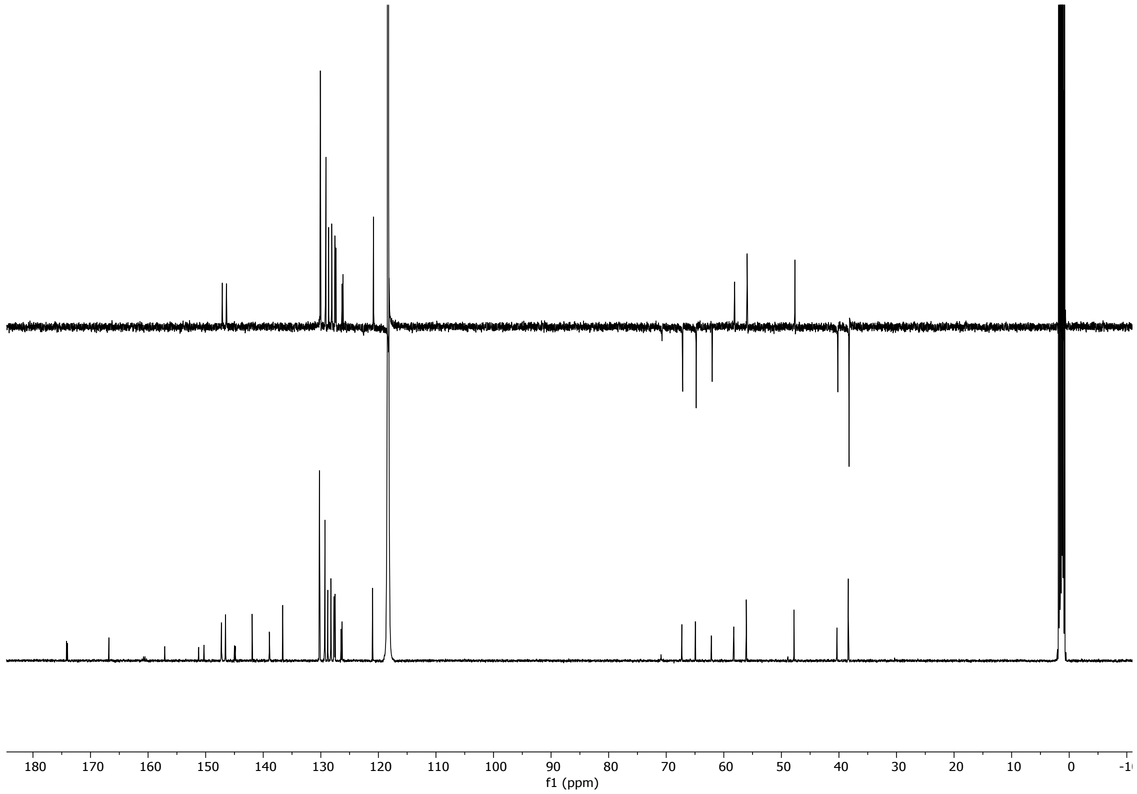

Figure S66: $^{13}C\{^{1}H\}$ NMR and DEPT (125 MHz, CD_3CN) spectrum of 9·2TFA.

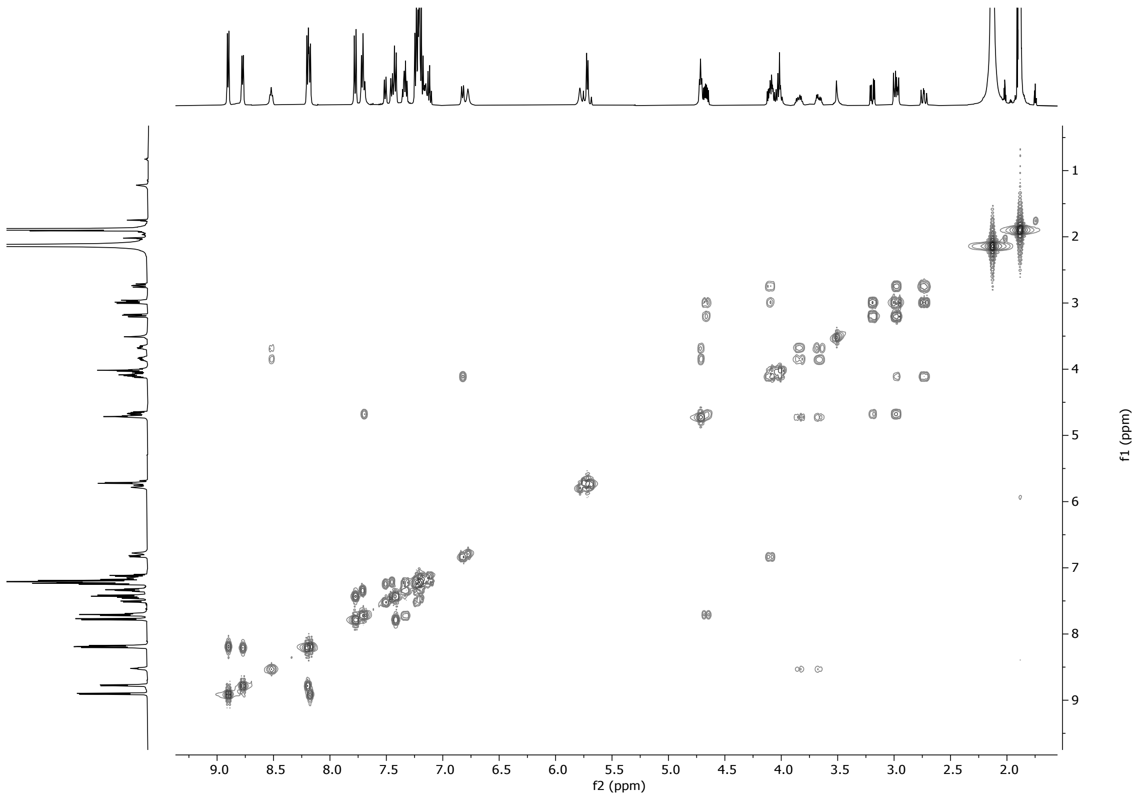

Figure S67: COSY (500 MHz, CD_3CN) spectrum of 9·2TFA.

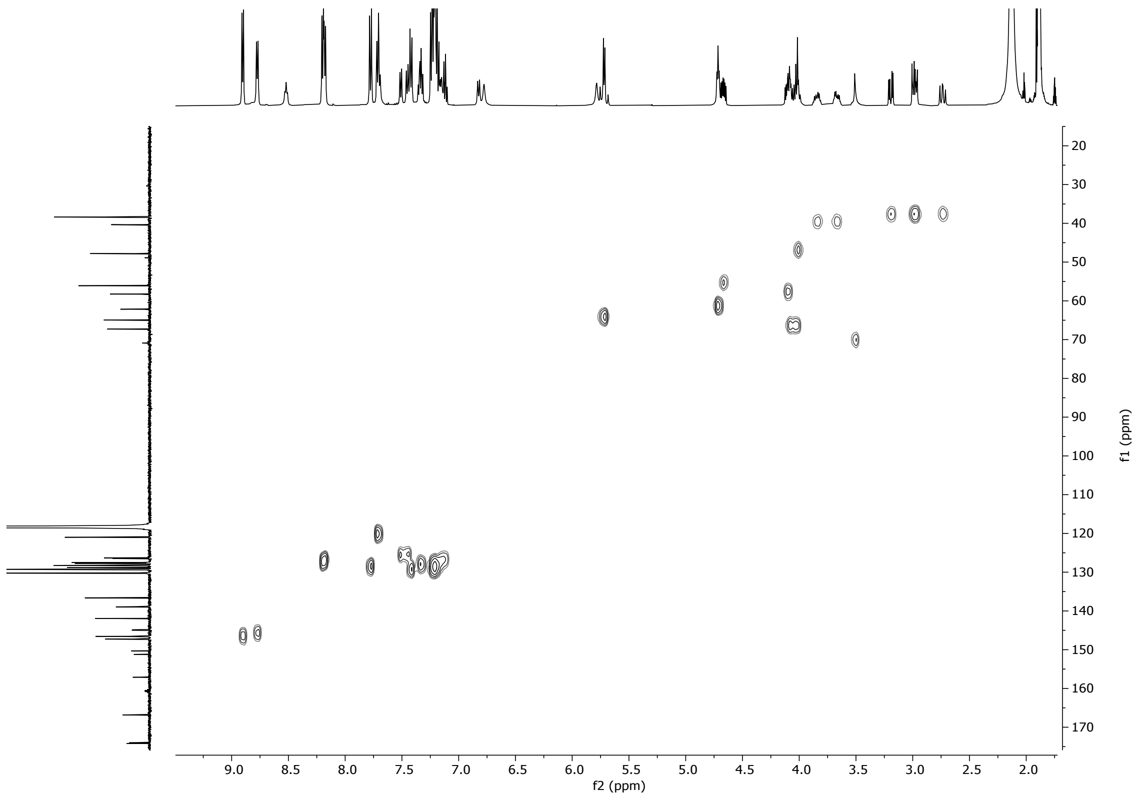

Figure S68: HSQC (500 MHz, CD_3CN) spectrum of 9·2TFA.

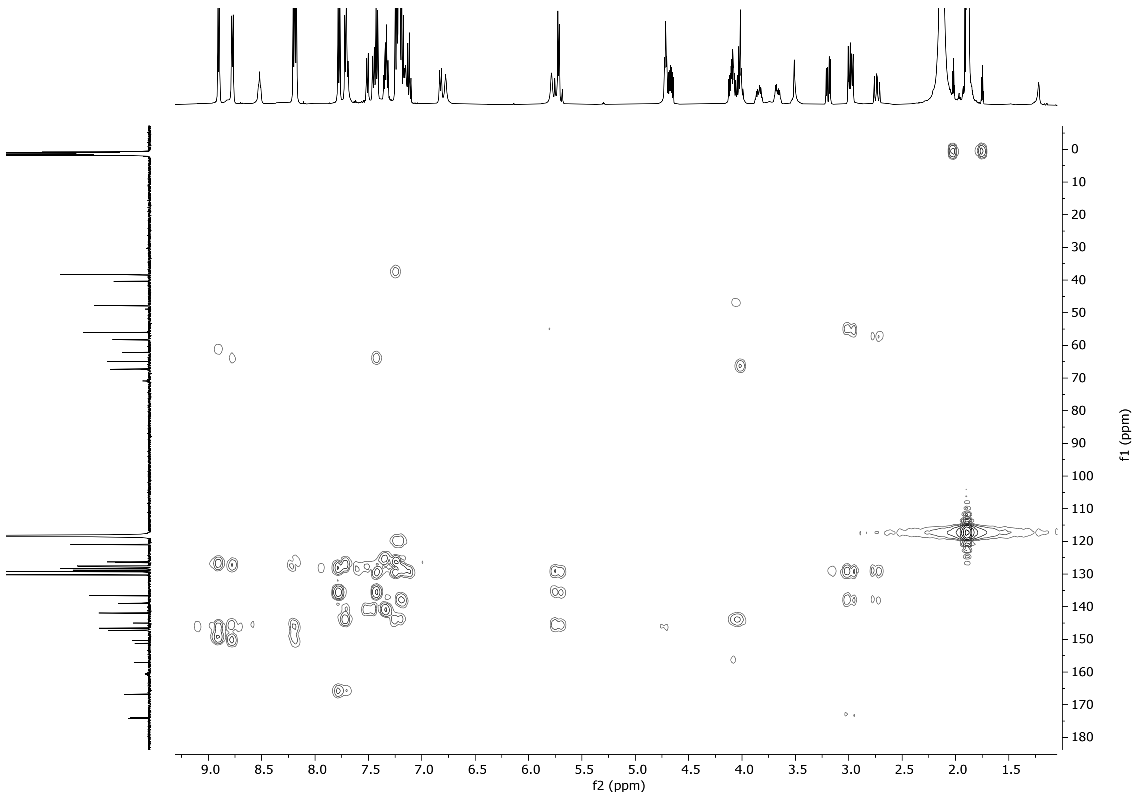

Figure S69: HMBC (500 MHz, CD_3CN) spectrum of 9·2TFA.

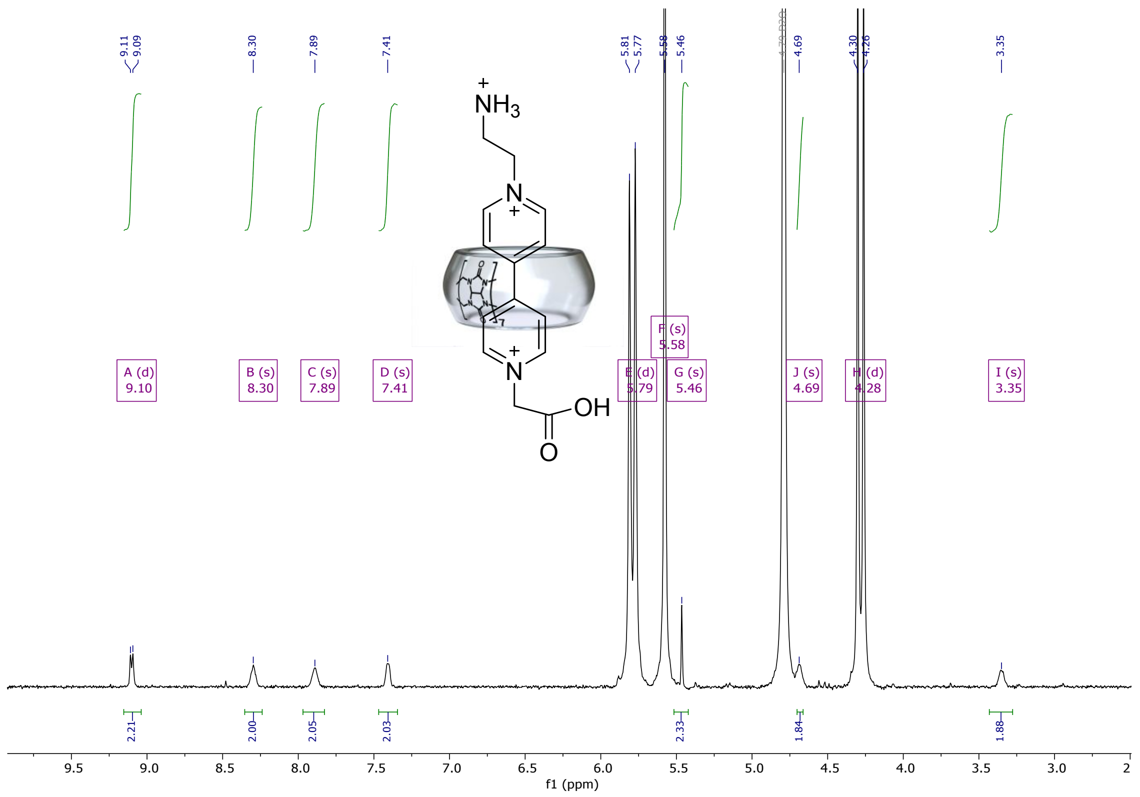

Figure S70: ^{1}H NMR (500 MHz, D_2O) spectrum of 2H⊂CB[7].

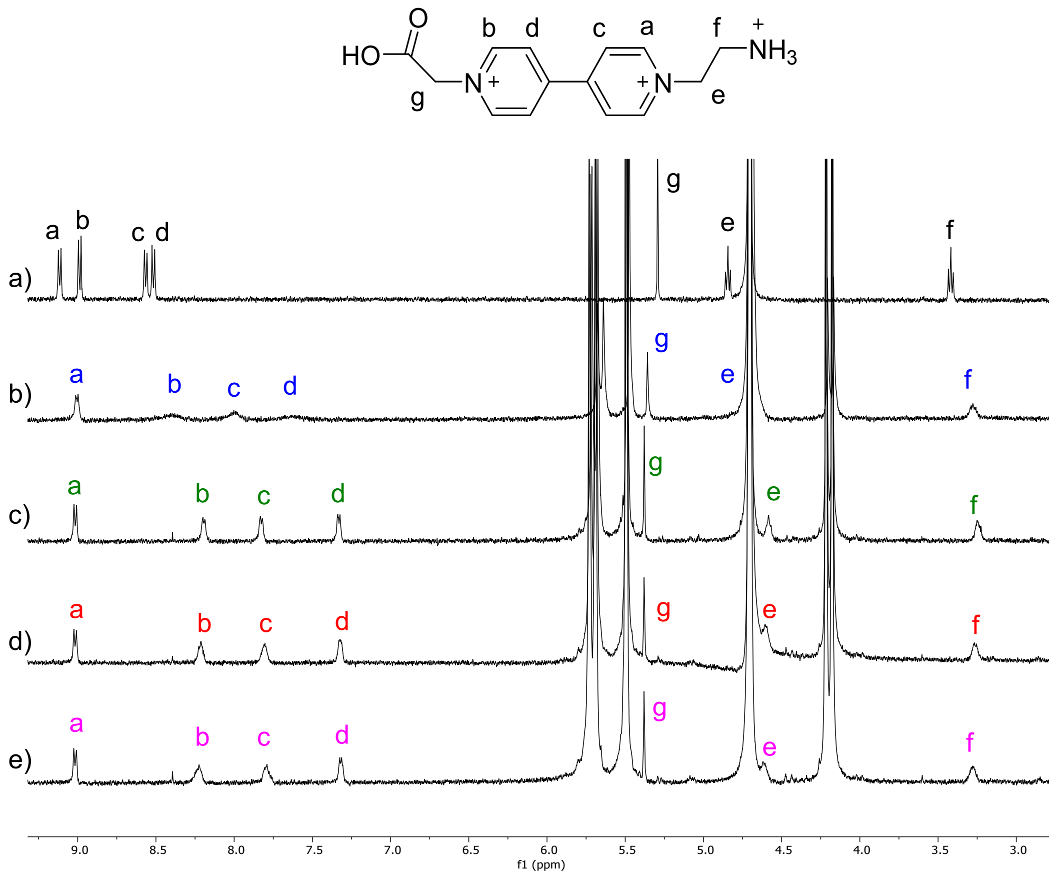

Figure S71: Partial NMR ^1H (500 MHZ, D_2O, pD=7) of: a) 2H·3Cl b) 2H·3Cl + 0.5 eq CB[7] c) 2H·3Cl + 1 eq CB[7] d) 2H·3Cl + 2 eq CB[7] e) 2H·3Cl + 3 eq CB[7].

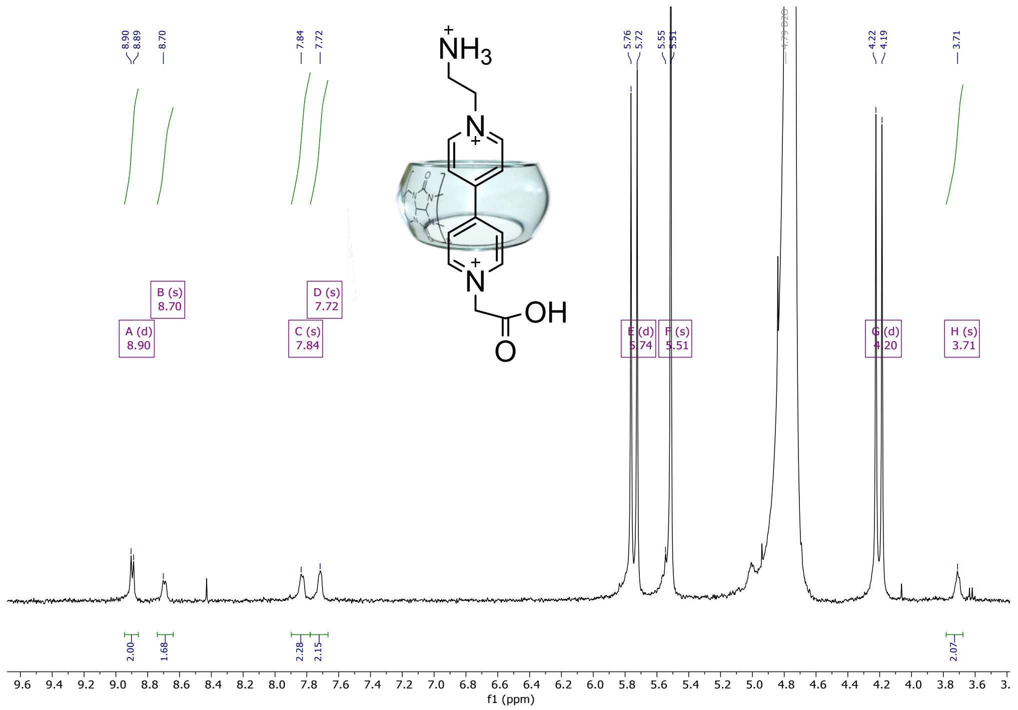

Figure S72: ^1H NMR (500 MHz, D_2O) spectrum of 2H⊂CB[8].

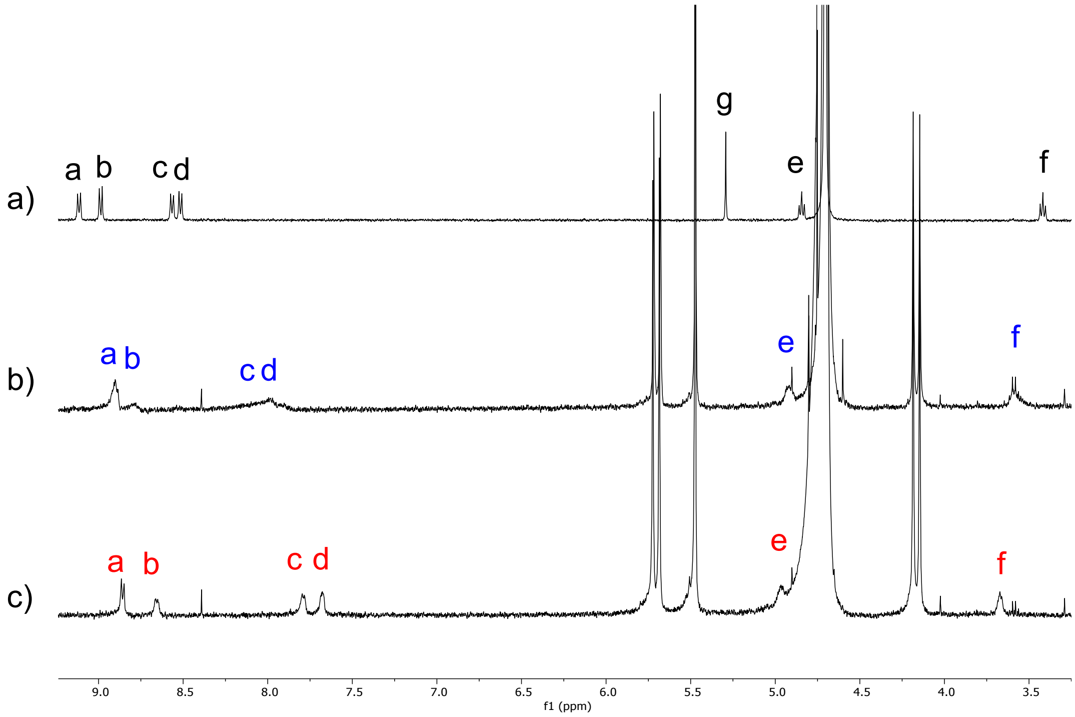

Figure S73: Partial NMR ^{1}H (500 MHZ, D_2O, pD=7) of: a) 2H·3Cl b) 2H·3Cl + excess of CB[8].

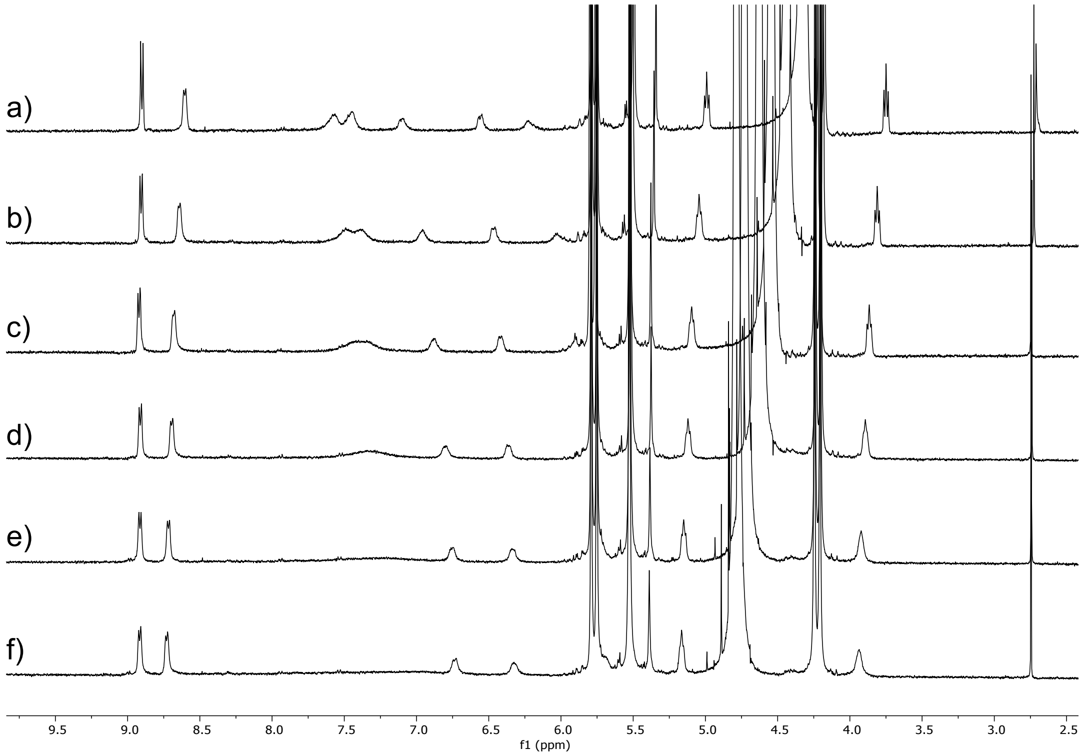

Figure S74: T. Var. (400 MHz, D_2O, pD=7) spectrum of a mixture of 2H·3Cl, CB[8], and 2,7-DHN at: a) 343.15 K b) 333.15 K c) 323.15 K d) 313.15 K e) 303.15 K f) 298.15 K.

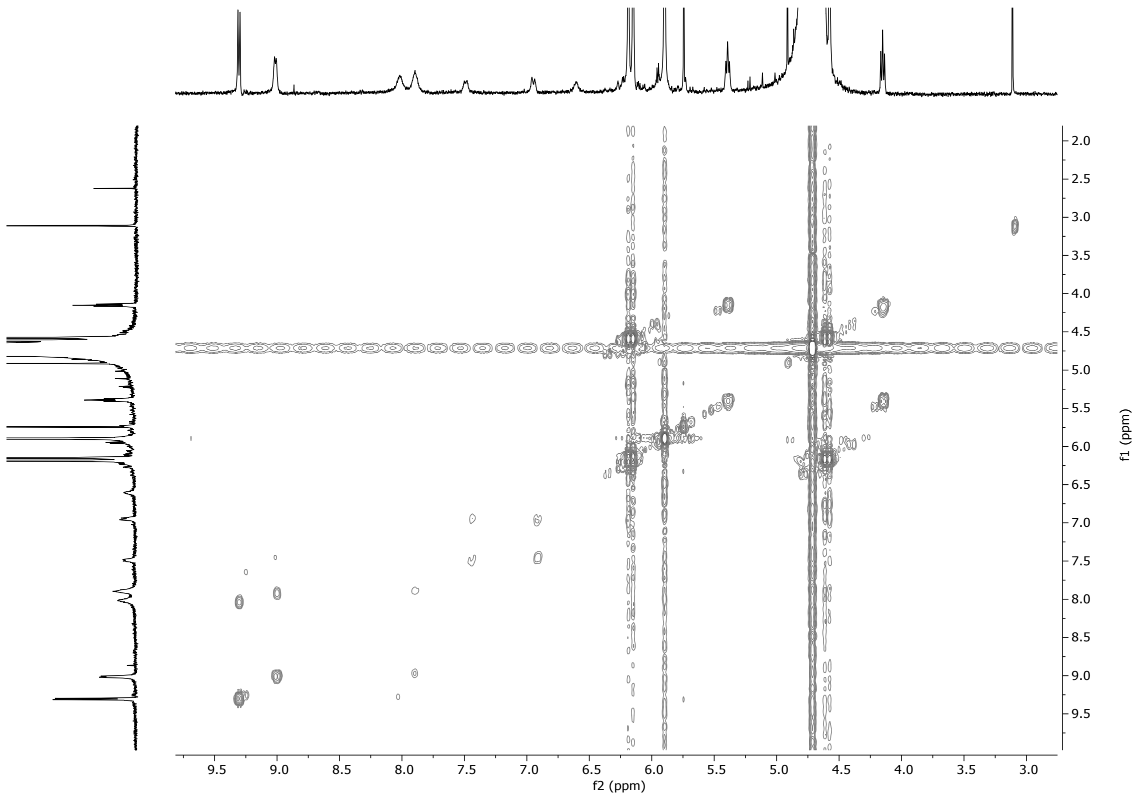

Figure S75: COSY (400 MHz, D_2O, T=343.25 K) spectrum of a mixture of 2H·3Cl, CB[8], and 2,7-DHN.

SP14: Self-assembly of **7**·2TFA with cucurbit[8]uril:

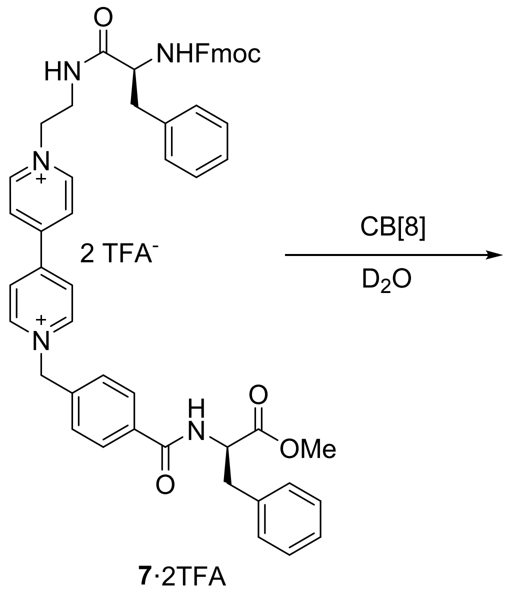

7·2TFA

To a 1mM solution of guest **7·**2TFA in D_2O with 50 mM phosphate buffer solution at pD=7 different amounts of CB[8] (as solid) were added until the solution reached saturation. The resulting mixture was heated for 24 hours at 60 °C and filtered off.

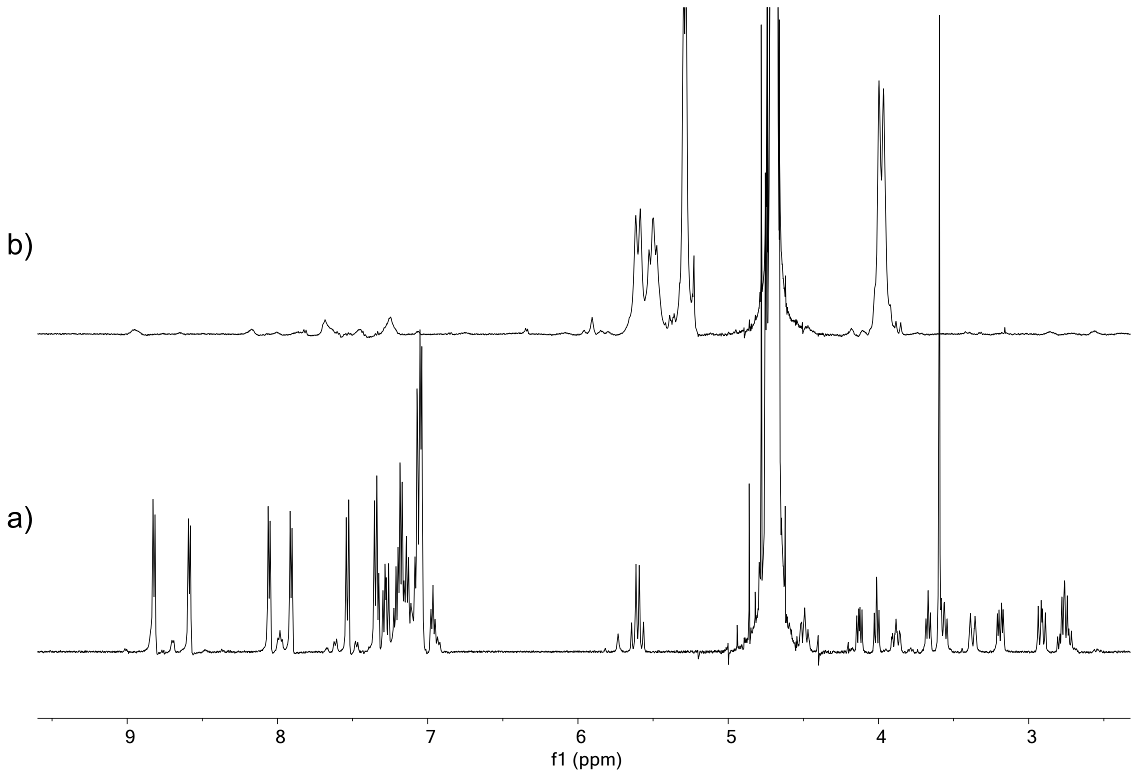

Figure S76. Partial NMR ^1H (500 MHZ, D_2O, pD=7) of: a) 7·2TFA b) 7·2TFA + excess of CB[8].
